# Overcoming times of crisis: unveiling coping strategies and mental health in a transnational general population sample during and after the COVID-19 pandemic

**DOI:** 10.1186/s40359-024-02001-3

**Published:** 2024-09-19

**Authors:** Timo Schurr, Beatrice Frajo-Apor, Silvia Pardeller, Barbara Plattner, Franziska Tutzer, Anna Schmit, Andreas Conca, Martin Fronthaler, Christian Haring, Bernhard Holzner, Markus Huber, Josef Marksteiner, Carl Miller, Verena Perwanger, Roger Pycha, Martin Schmidt, Barbara Sperner-Unterweger, Alex Hofer

**Affiliations:** 1 Department of Psychiatry, Psychotherapy, Psychosomatics and Medical Psychology, Division of Psychiatry I, Medical University Innsbruck, Anichstraße 35, Innsbruck, 6020 Austria; 2 Department of Psychiatry, Sanitary Agency of South Tyrol, General Hospital of Bolzano, Bolzano, Italy; 3 Sanitary Agency of South Tyrol, Therapy Center Bad Bachgart, Rodengo, Italy; 4 Department of Psychiatry and Psychotherapy B, State Hospital Hall in Tyrol, Hall in Tyrol, Austria; 5 Department of Psychiatry, Sanitary Agency of South Tyrol, General Hospital of Brunico, Brunico, Italy; 6 Department of Psychiatry and Psychotherapy A, State Hospital Hall in Tyrol, Hall in Tyrol, Austria; 7 Department of Psychiatry, County Hospital Kufstein, Kufstein, Austria; 8 Department of Psychiatry, Sanitary Agency of South Tyrol, General Hospital of Merano, Merano, Italy; 9 Department of Psychiatry, Sanitary Agency of South Tyrol, General Hospital of Bressanone, Bressanone, Italy; 10 Department of Psychiatry, County Hospital Lienz, Lienz, Austria; 11 Department of Psychiatry, Psychosomatics and Medical Psychology, Division of Psychiatry II, Medical University Innsbruck, PsychotherapyInnsbruck, Austria

**Keywords:** COVID-19, Crisis, Coping, Mental health, Psychological distress, Austria, Italy

## Abstract

**Background:**

The COVID-19 pandemic has had an unparalleled impact, precipitating not only direct threats to physical health but also widespread economic and psychological challenges. This study aims to explore the dynamics of coping behaviour and psychological distress (PD) across different phases of the pandemic within an adult general population sample, spanning Austria and Italy.

**Methods:**

An online questionnaire-based panel study was conducted between 2020 and 2023 including three measurements. We collected data on sociodemographic variables, coping responses (Brief COPE), and PD (Brief-Symptom-Checklist). Statistical analyses were conducted within a linear-mixed-model framework. Multiple imputation and sensitivity analysis were applied to validate the results obtained by complete case analysis.

**Results:**

The study follows 824 participants and reveals a marginal decrease in overall PD from the first to the second follow-up, particularly in clinically relevant phobic anxiety (35.6% and 34.5% to 25.4%). Most coping behaviours exhibited stable mean-levels with intra-individual variability across the study period. Maladaptive coping strategies were consistently linked to increased PD, whereas adaptive strategies were associated with decreased PD.

**Conclusion:**

Our findings underscore the complex nature of coping behaviours and PD during and after the pandemic, suggesting that while mean-levels of PD and coping responses remained relatively stable, most coping strategies were subject to intra-individual change. Maladaptive strategies were associated with increased PD, pinpointing to the need for interventions that establish the foundation for adaptive coping mechanisms and promote their application. Further research should explore the reciprocal influences of mental health on coping behaviour, incorporating interventional designs to unravel the nuances of these relationships.

**Supplementary Information:**

The online version contains supplementary material available at 10.1186/s40359-024-02001-3.

## Background

The COVID-19 pandemic was unprecedented in modern history in terms of its global impact, speed of progression, and the extraordinary measures taken to mitigate its effects. This event took the world off guard—not only posing a direct threat to physical health [1] but ushered in economic uncertainties [2] and psychological challenges like social isolation, lifestyle disruptions, and health fears.

The mental health of many individuals suffered and studies suggest that at the pandemic’s onset anxiety, depressive symptoms, and psychological distress (PD) were on the rise with people affected to varying degrees, depending on e.g. gender, financial status, or pre-existing medical conditions [3–6]. For example research has shown that certain populations, particularly healthcare workers, faced heightened levels of anxiety and depression due to the demands of their roles during the pandemic [7]. Yet, mental health deterioration associated with the pandemic was not permanently present for the majority, including those with pre-existing mental health disorders (MHD; [8–11].

The ability to adapt to these new circumstances and to alleviate mental burden can broadly be explained by several factors: by those that are linked to external factors altering the perception of e.g. health risks, financial risks, or impairments due to measures taken to minimize the propagation of the virus [12–14], and by individual psychological factors serving as protective or risk factors for mental health [15]. Concerning the latter, Miglani and colleagues demonstrated that resilience or healthy coping strategies might be a valuable mitigation strategy to reduce psychological distress [16]. Lazarus and Folkman’s Transactional Model of Stress and Coping posits that coping is a dynamic process involving continuous appraisal and reappraisal of stressors [17]. According to this framework, individuals first engage in primary appraisal, evaluating whether a situation is irrelevant, benign-positive, or stressful. If deemed stressful, they then assess their available resources and options in the secondary appraisal phase, determining how to cope with the stressor. The response to the stressor is mainly determined by the individuals’ resources, capabilities, personal preferences, or experiences. According to the model by Lazarus and Folkman, emotion-oriented coping (i.e. regulating or modifying the reaction towards the stressor) is often used when a situation is perceived as uncontrollable. On the other hand, problem-oriented coping (i.e. attempting to change the situation) is used when the situation is perceived as controllable. This theoretical framework provides a basis for understanding how pandemic-related stressors, such as health risks and financial uncertainties, may influence individuals’ selection of coping strategies and the associations with PD.

Austria and Italy were impacted particularly badly at the beginning of the COVID-19 pandemic, creating a breeding ground for various stressors. In early 2020, the Italian healthcare system was stretched to its limits, unable to care effectively for the numerous patients [18] and making COVID-19 the leading cause of death during that time [19, 20]. Within that same period, a large SARS-CoV-2 cluster formed in the Austrian municipality of Ischgl in Tyrol, marking the beginning of the virus spreading in Austria and Northern Europe [21]. However, early implementation of a strict control policy (e.g. lockdown) proved effective, leading to fewer deaths compared to Italy [22] but those precautions also had negative consequences [23].

In that first phase, coping behaviour was studied around the globe among individuals from the general population [24–28]. Götmann and Bechtoldt explored how individuals from Germany did cope with COVID-19 associated restrictions of personal freedom. Their analysis revealed that problem-focused coping (e.g. active coping, positive reframing) was positively associated with well-being, whereas avoidant-oriented coping (e.g. distraction, denial) had the opposite relationship [29]. Additional evidence was provided by Rogowska and colleagues, showing that frequent usage of emotion-focused coping strategies (e.g. self-blame, venting) was associated with higher stress levels in Polish students, whereas individuals engaging in problem-focused coping reported lower perceived stress [30]. Although some studies also included Austria and Italy [31–33], they primarily focused on healthcare personnel in the latter [7, 34–36].

Firstly, according to most recent studies, we hypothesized that PD would decrease as pandemic restrictions were lifted. Secondly, previous research left an equivocal picture of people’s coping utilization, showing that it can be characterized by both malleable and stable components. Recent studies suggest that normative stability (mean-level change) and differential variability (intra-individual change) of coping responses are predominate in the context of extreme or life-threatening circumstances and the context of the COVID-19 pandemic [37, 38]. Hence, we assumed that the extent to which people utilized coping would not vary between the dates of measurements (indicated by mean-score stability) for most strategies. Moreover, we supposed high flexibility in intra-individual changes for most coping responses. Thirdly, we hypothesized that coping responses’ impact on PD would not differ between measurements.

## Materials and methods

### Design, participants and setting

**Fig. 1:**
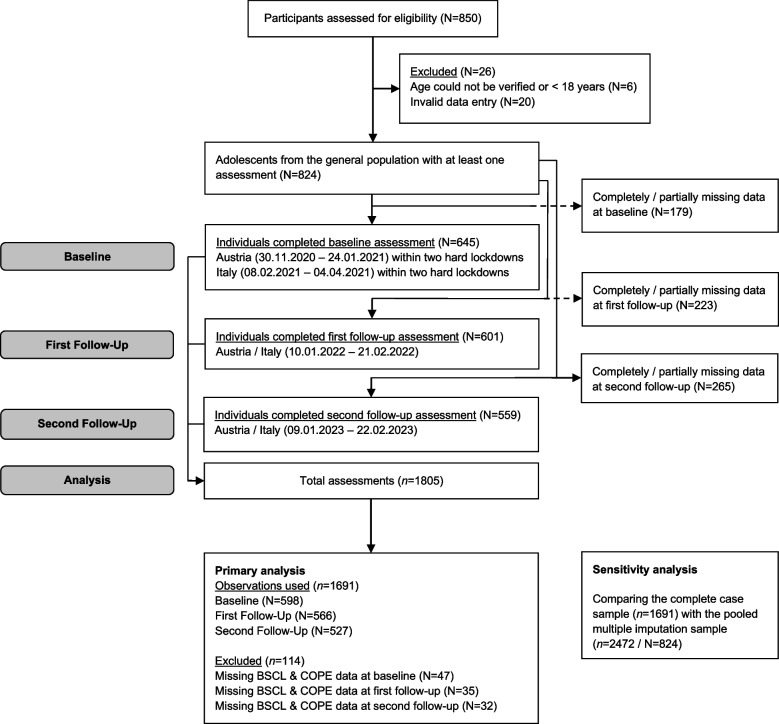
Study procedure flow chart

This online questionnaire-based panel study was conducted over a period of three years involving three annual measurements. The study was set up in Tyrol (Austria) and South Tyrol (Italy), characterized by a mix of urban and rural areas, allowing for a diverse representation of participants in terms of geographic location and lifestyle. This study utilized a non-probability sampling approach, specifically convenience sampling. The sample consists of individuals of legal age (18 +) from the general population with valid data entries. Participants were recruited via multiple channels, including social media platforms, flyers distributed in public places, and print media advertisements in local newspapers. These recruitment methods were chosen to maximize reach and participation across various demographic groups, although the sample may not fully represent the entire population. Figure 1 depicts the participants flow through the study. In December 2020, Tyrol experienced a two-week light lockdown between two hard lockdowns expanding over the entire baseline assessment. These hard lockdowns were characterized by rules that applied during the day (e.g. curfew, closures of most shops, leisure facilities, and service providers for body-related services; [39]. In South Tyrol, comparable hard lockdown measures were taken during baseline assessment, i.e. from 5 February 2021 (three weeks) and from 15 March 2021 until mid-April 2021. The first COVID-19 vaccinations in Austria and Italy were rolled out from 27 December 2020. At the beginning of 2022, while the second survey was enrolled, measures changed in both countries with an increased focus on vaccinations and general hygiene measures such as face masks. By the third survey in 2023, COVID-19 measures had largely been lifted in both countries. Special precautions remained in facilities treating/housing vulnerable people, such as hospitals.

### Participants characteristics and flow through the study

Figure 1 depicts the study procedure chart. In total, *N* = 850 were assessed for eligibility and signed informed consent. 26 participants had to be excluded due to invalid data, unverifiable or low age and eventually, data of 824 people with at least one measurement during the study period was available. This yielded a dataset of *n* = 1805 assessments that contained (partially) complete information regarding demographic and clinical variables. However, another 114 participants had to be excluded from the primary analysis due to missing BSCL and Brief COPE data. In total, *n* = 1691 observations with complete data were used for the primary analysis.

### Variables and measures

#### Sociodemographic and clinical data

Sociodemographic data collected various factors, including gender, age, place of residence, relationship status, educational background, current employment status, and living arrangements. Participants were also asked if they had been exposed to or experienced more violence since the outbreak of the pandemic. In addition, they were requested to share information on their COVID-19 vaccination status and if they were tested positive for COVID-19, as well as any severe physical health issues and whether, and if so, what type of treatment they were currently receiving due to MHD.

#### Coping responses

Coping responses were measured with the German and Italian version of the Brief COPE [40–42]. This short version of the multidimensional coping inventory (COPE; [43] consists of 28 items evaluating 14 coping responses derived from theoretical frameworks [44–46]: *acceptance*, *active coping*, *behavioural disengagement*, *denial*, *emotional support*, *humour*, *informational support*, *planning*, *positive reframing*, *religion*, *self-blame*, *self-distraction*, *substance use*, and *venting*. Participants were instructed to assess the alignment of each statement with their thoughts and actions during challenging or unpleasant situations. Responses were provided on a 4-point scale, with 1 indicating ‘not at all’, 2 representing ‘a little bit’, 3 signifying ‘medium amount’, and 4 implying ‘a lot’. Mean scores for each coping response were calculated resulting in a possible scale range of 1–4.

#### Psychological distress

PD was assessed using the German and Italian version of the Brief-Symptom-Checklist (BSCL; [47, 48]. This self-report instrument consists of 53 items that participants rate on a 5-point Likert-type scale ranging from 0 ‘not at all’, 1 ‘a little bit’, 2 ‘moderately’, 3 ‘quite a bit’ to 4 ‘extremely’. Participants were asked to indicate complaints they had had in the last seven days. The BSCL measures PD across nine sub-domains, each evaluated by specific subscales: *anger-hostility*, *anxiety*, *depression*, *paranoid ideation*, *phobic anxiety*, *psychoticism*, *somatization*, *interpersonal sensitivity*, and *obsessive-compulsiveness*.

The sub-domain scores were calculated by adding up the respective items’ values. Additionally, a composite score called the Global Summary (GS) was calculated by combining the sub-domains scores resulting in a possible scale range of 0–212. To allow meaningful interpretation, these raw scores were transformed into age- and gender-specific normative T-scores by using a standardization reference table. The average T-score lies within a mean of 50 ± one standard deviation (10 points). T-scores ≥ 63 indicate clinically significant PD.

### Data collection

Data collection was realised through the Computer-based Health Evaluation System (CHES; [49]. For organizational reasons, the baseline assessment period (t0) differed between Austria (30 November 2020 to 24 January 2021) and Italy (8 February 2021 to 4 April 2021). The first follow-up assessment (t1) was conducted from 10 January 2022 to 21 February 2022, the second follow-up assessment (t2) took place between 9 January 2023 and 22 February 2023. During the assessments, participants were instructed to reflect specifically on their current circumstances.

### Statistical methods

#### Statistical analysis

Statistical analysis was conducted with *SPSS,* version 29.0 [50] and *R,* version 4.2.3 [51] using the package *nlme,* version 3.1–163 [52] and *ggplot2,* version 3.4.4 [53]. The significance level was set to α = 0.05, conducted statistical tests were two-tailed. To adjust for multiple testing, Benjamini–Hochberg correction was applied with a false discovery control level of 5%. Participants without missing data regarding the Brief COPE and BSCL at least in one assessment were included in the primary analysis.

Sociodemographic data is described by mean, standard deviation, median, interquartile range, and counts. Sample properties in terms of coping responses (Brief COPE) and PD (BSCL) are given by means of scale’s reliability (McDonald’s ω; [54], proportions of response categories, and age- and gender-specific normative T-scores ≥ 63. Comparisons between men and women as well as between Tyrol (Austria) and South-Tyrol (Italy) regarding coping and psychological distress were provided in the Supplementary Material. By means of McNemar test marginal homogeneity regarding exposure to violence, increased propensity for violence, physical health problems and treatment due to MHD, and clinically significant PD T-Scores were analysed. Reported Cohen’s *g* and *d* effect sizes can be interpreted as *g* = 0.05 < 0.15; *d* = 0.20 < 0.50 small; g = 0.15 < 0.25; *d* = 0.50 < 0.80, medium; and large for *g* ≥ 0.25; *d* ≥ 0.80 [55].

First, potential within-group mean-level changes in PD sub-domain scores and GS as well as coping responses were analysed within a linear mixed model framework. Regarding coping responses, intraclass correlation coefficients (ICC) were calculated to assess differences in intra-individual change (i.e. differential stability). In case of non-normally distributed standardized residuals (assessed through skewness, kurtosis, QQ-plot and histogram), the BSCL variable was transformed appropriately (logarithmic) to provide robust standard errors and *p*-values. Since all statistical outliers fell within the range of the used scales, they were net excluded from analyses.

To test the third hypothesis a null-model including PD as dependent variable was initially specified. The unconditional ICC was then calculated, where τ^2^ is the between-subject variance and σ^2^ is the within-subject variance with values ranging from 0 to 1:

$$\text{ICC}=\frac{{\uptau }_{0}^{2}}{{\upsigma }^{2}+ {\uptau }_{0}^{2}}$$

Next, a series of univariate models (Model 1.1 – 1.14) with random intercept and fixed slope including each COPE sub-domain separately were established. Then the interaction term between coping responses × time was included (Model 2.1—2.14). Following univariate modelling we proceeded to a multivariable model including all COPE sub-domains attaining a *p*-value below 0.05 in the univariate model after Benjamini–Hochberg correction was applied. To account for possible differences both, the univariate and multivariable models additionally included age, gender and place of residence as covariate. Similarly to the univariate model building approach, the interaction between coping response × time was not included at first (Model 3), followed by a model including the interaction term (Model 4). In case the interaction term was not statistically significant, the model with parsimonious properties (i.e. fewer parameters) was chosen.

#### Multiple imputation and sensitivity analysis

A detailed description of the multiple imputation procedure and the sensitivity analysis can be found in the supplementary materials.

## Results

Women accounted for almost three quarters (~ 72%) of the total sample. Participants’ mean age was in the mid-forties (baseline: Mean = 44.4, SD = 13.2), and nearly two thirds were from Austria (~ 62%). Over 76% reported having a stable relationship and living with another person in the same household. Children resided in 43% of households, of whom 47% were taught by their parents. More information regarding sociodemographic variables can be found in Table 1. At baseline 12.1% reported on having tested positive for COVID-19, increasing to 27.2% at first and 73.0% at second follow-up. 91.0% of participants were vaccinated at t1, 91.8% at second follow-up measurement.

**Table 1:** Sociodemographic data of participants at baseline (t0) and follow-up (t1 & t2)

Variable	Measurement	Mean (SD) [range] / Median [IQR] or N (%)	Missing N (%)
**Gender**			
Male	t_0_	169/ 598 (28.3%)	t_0_: 0/598 (0%)
	t_1_	158/ 566 (27.9%)	t_1_: 0/566 (0%)
	t_2_	144/ 527 (27.3%)	t_2_: 0/527 (0%)
Female	t_0_	429/ 598 (71.7%)	
	t_1_	408/ 566 (72.1%)	
	t_2_	383/ 527 (72.7%)	
**Age** (Years)	t_0_	44.4 (13.2) [18–96] / 44 [35–54]	t_0_: 0/598 (0%)
	t_1_	45.8 (13.5) [19–97] / 45 [36–55]	t_1_: 0/566 (0%)
	t_2_	47.4 (13.3) [20–98] / 47 [37–57]	t_2_: 0/527 (0%)
**Residence**			
Tyrol (Austria)	t_0_	351/ 598 (58.7%)	t_0_: 0/598 (0%)
	t_1_	359/ 566 (63.4%)	t_1_: 0/566 (0%)
	t_2_	337/ 527 (63.9%)	t_2_: 0/527 (0%)
South Tyrol (Italy)	t_0_	247/ 598 (41.3%)	
	t_1_	207/ 566 (36.6%)	
	t_2_	190/ 527 (36.1%)	
**Relationship**			
Single	t_0_	140/ 598 (23.4%)	t_0_: 3/598 (0.5%)
	t_1_	130/ 566 (23.0%)	t_1_: 1/566 (0.2%)
	t_2_	122/ 527 (23.1%)	t_2_: 0/527 (0%)
Fixed partnership	t_0_	455/ 598 (76.1%)	
	t_1_	435/ 566 (76.9%)	
	t_2_	405/ 527 (76.9%)	
**Pregnant**	t_0_	8 / 429 (1.9%)	t_0_: 4/598 (0.7%)
	t_1_	11 / 408 (2.7%)	t_1_: 0/566 (0%)
	t_2_	4 / 383 (1.0%)	t_2_: 3/527 (0.6%)
**People in household**	t_0_	**one**: 132 / 598 (22.1%)	t_0_: 19/598 (3.2%)
		**two**: 170 / 598 (28.4%)	
		**three**: 108 / 598 (18.1%)	
		**four—eight**: 168 / 598 (28.1%)	
**Children in household**	t_0_	**none**: 340/ 598 (56.8%)	t_0_: 0/598 (0%)
		**one**: 90 / 598 (15.1%)	
		**two**: 117 / 598 (19.6%)	
		** ≥ three**: 51 / 598 (8.5%)	
**Currently teaching or homeschooling children**	t_0_	122/258 (47.3%)	t_0_: 0/598 (0%)
**Flat size** (m^2^)	t_0_	103 (47) [20–450] / 100 [73–120]	t_0_: 29/598 (4.8%)
per person (Flat size/ Ppl. in household)	t_0_	46 (26) [8–180] / 40 [28–55]	t_0_: 39/598 (6.5%)
**Garden or balcony**	t_0_	561 (93.8%)	t_0_: 1/598 (0.2%)

*Abbreviations*: *SD* Standard deviation, *IQR* Inter quartile range

Clinically relevant PD was evident in almost 25% of the sample (Table 2). T-scores in the clinically relevant range were particularly found in terms of *phobic anxiety* at baseline (35.6%) and the first follow-up (34.5%) measurement. However, this prevalence decreased significantly at the third measurement (25.4%; *p* < 0.0001).

**Table 2:** T-scores, means, standard deviations, and reliability of BSCL scales at baseline (t0) and follow-up (t1 & t2)

**Scales**	**Measurement**	**Number of items**	T-score (≥ 63) **N** (%)	**Mean** (SD)	**Estimate**	**p**_**BH**_**-value**^a^	**Reliability** (ω)
Anger-hostility	t0	5	175/598 (29.3)	2.80 (3.10)	t_0_-t_1:_ 0.010	t_0_-t_1:_ 0.6469	.787
	t1		156/566 (27.6)	2.85 (3.05)	t_0_-t_2:_ -0.071	t_0_-t_2:_ 0.0033	.805
	t2		138/527 (26.2)	2.45 (2.95)	t_1_-t_2:_ -0.081	t_1_-t_2:_ 0.0012	.797
Anxiety	t_0_	6	121/598 (20.2)	3.12 (3.96)	t_0_-t_1:_ 0.011	t_0_-t_1:_ 0.6121	.873
	t_1_		119/566 (21.0)	3.24 (4.02)	t_0_-t_2:_ -0.039	t_0_-t_2:_ 0.1301	.862
	t_2_		100/527 (19.0)	2.94 (3.78)	t_1_-t_2:_ 0.022	t_1_-t_2:_ 0.0786	.865
Depression	t_0_	6	136/598 (22.7)	3.84 (4.74)	t_0_-t_1:_ 0.015	t_0_-t_1_: 0.5476	.902
	t_1_		136/566 (24.0)	3.96 (4.80)	t_0_-t_2:_ -0.053	t_0_-t_2_: 0.0698	.903
	t_2_		113/527 (21.4)	3.66 (4.68)	t_1_-t_2:_ -0.069	t_1_-t_2_: 0.0282	.908
Paranoid ideation	t_0_	5	144/598 (24.1)	3.20 (3.65)	t_0_-t_1_: 0.029	t_0_-t_1_: 0.5621	.810
	t_1_		152/566 (26.9)	3.40 (3.65)	t_0_-t_2_: 0.006	t_0_-t_2_: 0.7998	.815
	t_2_		134/527 (25.4)	3.30 (3.60)	t_1_-t_2_: -0.022	t_1_-t_2_: 0.5621	.821
Phobic anxiety	t_0_	5	213/598 (35.6)	2.50 (3.15)	t_0_-t_1_: 0.008	t_0_-t_1_: 0.7279	.754
	t_1_		195/566 (34.5)	2.60 (3.35)	t_0_-t_2_: -0.121	t_0_-t_2_: < 0.0001	.802
	t_2_		134/527 (25.4) ↓	2.00 (3.10)	t_1_-t_2_: -0.129	t_1_-t_2_: < 0.0001	.819
Psychoticism	t_0_	5	155/598 (25.9)	1.85 (2.80)	t_0_-t_1_: 0.018	t_0_-t_1_: 0.5411	.791
	t_1_		144/566 (25.4)	1.95 (3.00)	t_0_-t_2_: -0.003	t_0_-t_2_: 0.8904	.808
	t_2_		129/527 (24.5)	1.85 (2.85)	t_1_-t_2_: -0.021	t_1_-t_2_: 0.5411	.802
Somatization	t_0_	7	89/598 (14.9)	2.73 (4.13)	t_0_-t_1_: 0.047	t_0_-t_1_: 0.0474	.870
	t_1_		101/566 (17.8)	3.15 (4.27)	t_0_-t_2_: 0.011	t_0_-t_2_: 0.5805	.862
	t_2_		85/527 (16.1)	2.94 (3.99)	t_1_-t_2_: -0.036	t_1_-t_2_: 0.1086	.847
Interpersonal sensitivity	t_0_	4	141/598 (23.6)	2.72 (3.08)	t_0_-t_1_: 0.065	t_0_-t_1_: 0.0198	.817
	t_1_		145/566 (25.6)	3.00 (3.04)	t_0_-t_2_: 0.002	t_0_-t_2_: 0.9396	.797
	t_2_		123/527 (23.3)	2.80 (3.08)	t_1_-t_2_: -0.063	t_1_-t_2_: 0.0198	.823
Obsessive-compulsiveness	t_0_	6	116/598 (19.4)	4.26 (4.44)	t_0_-t_1_: 0.085	t_0_-t_1_: 0.0012	.877
	t_1_		125/566 (22.1)	4.68 (4.50)	t_0_-t_2_: 0.044	t_0_-t_2_: 0.0937	.865
	t_2_		106/527 (20.1)	4.56 (4.38)	t_1_-t_2_: -0.041	t_1_-t_2_: 0.0937	.875
Global severity	t_0_	53	139/598 (23.2)	29.08 (30.34)	t_0_-t_1:_ 1.644	t_0_-t_1:_ 0.0957	.973
	t_1_		144/566 (25.4)	31.01 (30.89)	t_0_-t_2:_ -1.212	t_0_-t_2:_ 0.1875	.974
	t_2_		123/527 (23.3)	28.70 (30.15)	t_1_-t_2:_ -2.856	t_1_-t_2:_ 0.0051	.975

*Abbreviations:* *SD* Standard deviation, *p*_*BH*_ Benjamini–Hochberg corrected *p*-value, ↓ According to McNemar test, significantly lower compared to t0 and t1 (*p* < 0.0001)

^a^Linear mixed model specifications: Independent variable = time; dependent variable = BSCL responses; Participants were considered subjects. Parameter estimates are based on the restricted maximum likelihood (REML) method. The variance–covariance structure of the random effects was specified as unstructured (UN), the variance–covariance structure of the within-group residuals (time) was specified as first-order autoregressive (AR1)

Supplementary Table 1 depicts participants’ exposure and propensity to violence, existing physical health problems, and treatment modalities due to MHD. According to McNemar’s test, the propensity for violence increased significantly between baseline (12.2%) and the second follow-up (18.6%). When analysing men and women (Supplementary Table 8) separately, this increase became not statistically significant anymore.

### Primary analysis

The calculated ICC was 0.796, indicating that 79.6% of the variance of PD could be accounted for by between-subject differences and 20.4% by within-subject changes between consecutive measurements. Next, a significant decrease in the BSCL total score from first to second follow-up (β = -2.856, d = 0.08; Table 2) was evident. Sub-domain analysis indicated decreased scores for *anger-hostility* between t0 and t2 (β = -0.071, d = 0.12) as well as between t1 and t2 (β = -0.081, d = 0.13), *depression* between t1 and t2 (β = -0.069, d = 0.06), and *phobic anxiety* between t0 and t2 (β = -0.121, d = 0.16) as well as between t1 and t2 (β = -0.129, d = 0.19). Increased scores were found regarding *somatization* between t0 and t1 (β = 0.047, d = 0.10) and in terms of *obsessive-compulsiveness* between t0 and t1 (β = 0.085, d = 0.09). *Interpersonal sensitivity* increased between t0 and t1 (β = 0.065, d = 0.09) and decreased again between t1 and t2 (β = -0.063, d = 0.07).

Regarding mean-level changes in coping responses, *behavioural disengagement* increased between baseline and both follow-up measurements (β_t0-t1_ = 0.113, d = 0.17; β_t0-t2_ = 0.144, d = 0.24, Table 3). *Positive reframing* marginally shrunk between baseline and second follow-up (β = -0.082, d = 0.11), whereas the *humour* mean value was slightly lower at first follow-up compared to baseline (β = -0.081, d = 0.10). Calculated ICC indicated moderate differential stability for most coping responses ranging from 0.419 (*behavioural disengagement*) to 0.571 (*self-blame*). Higher stability for coping responses within persons across measurements was found in terms of *humour* (ICC = 0.621), *substance use* (ICC = 0.721), and *religion* (ICC = 0.785). The score distributions of coping responses at baseline and follow-up measurements are depicted in Supplementary Fig. 1.

**Table 3:** Response portions, means, standard deviations and reliability of Brief COPE scales at baseline (t0) and follow-ups (t1 & t2)

**Scales**	**Measurement**	**Mean** (SD)	**Estimate**	**p**_**BH**_**-value**^**a**^	**ICC**	**Reliability** (*ω*)
Acceptance	t_0_	2.71 (0.76)	t_0_-t_1_: 0.032	t_0_-t_1:_ 0.4607	.505	.652
	t_1_	2.77 (0.78)	t_0_-t_2_: 0.058	t_0_-t_2:_ 0.2949		.722
	t_2_	2.77 (0.79)	t_1_-t_2_: 0.026	t_1_-t_2:_ 0.4607		.694
Active coping	t_0_	2.56 (0.72)	t_0_-t_1_: -0.032	t_0_-t_1:_ 0.3277	.448	.560
	t_1_	2.54 (0.71)	t_0_-t_2_: 0.037	t_0_-t_2:_ 0.3277		.581
	t_2_	2.60 (0.68)	t_1_-t_2_: 0.069	t_1_-t_2:_ 0.1194		.552
Behavioural disengagement	t_0_	1.56 (0.58)	t_0_-t_1_: 0.113	t_0_-t_1:_ < 0.0001	.419	.375
	t_1_	1.66 (0.62)	t_0_-t_2_: 0.144	t_0_-t_2:_ < 0.0001		.378
	t_2_	1.70 (0.61)	t_1_-t_2_: 0.031	t_1_-t_2:_ 0.2956		.296
Denial	t_0_	1.44 (0.59)	t_0_-t_1_: 0.013	t_0_-t_1:_ 0.6364	.454	.594
	t_1_	1.45 (0.59)	t_0_-t_2_: 0.030	t_0_-t_2:_ 0.6364		.500
	t_2_	1.47 (0.60)	t_1_-t_2_: 0.018	t_1_-t_2:_ 0.6364		.569
Emotional support	t_0_	2.54 (0.78)	t_0_-t_1_: -0.005	t_0_-t_1:_ 0.8904	.561	.687
	t_1_	2.52 (0.79)	t_0_-t_2_: -0.028	t_0_-t_2:_ 0.7247		.733
	t_2_	2.49 (0.77)	t_1_-t_2_: -0.024	t_1_-t_2:_ 0.7247		.729
Humour	t_0_	2.28 (0.80)	t_0_-t_1_: -0.081	t_0_-t_1:_ 0.0276	.621	.625
	t_1_	2.20 (0.77)	t_0_-t_2_: -0.020	t_0_-t_2:_ 0.5267		.576
	t_2_	2.24 (0.82)	t_1_-t_2_: 0.061	t_1_-t_2:_ 0.0860		.637
Informational support	t_0_	2.18 (0.80)	t_0_-t_1_: 0.002	t_0_-t_1:_ 0.9610	.534	.825
	t_1_	2.17 (0.80)	t_0_-t_2_: 0.026	t_0_-t_2:_ 0.6819		.831
	t_2_	2.19 (0.77)	t_1_-t_2_: 0.025	t_1_-t_2:_ 0.1971		.797
Planning	t_0_	2.69 (0.71)	t_0_-t_1_: 0.018	t_0_-t_1:_ 0.5822	.460	.535
	t_1_	2.73 (0.69)	t_0_-t_2_: -0.044	t_0_-t_2:_ 0.2876		.505
	t_2_	2.66 (0.72)	t_1_-t_2_: -0.061	t_1_-t_2:_ 0.1971		.590
Positive reframing	t_0_	2.69 (0.78)	t_0_-t_1_: -0.022	t_0_-t_1:_ 0.4972	.522	.700
	t_1_	2.68 (0.74)	t_0_-t_2_: -0.082	t_0_-t_2:_ 0.0444		.672
	t_2_	2.61 (0.75)	t_1_-t_2_: -0.060	t_1_-t_2:_ 0.1109		.681
Religion	t_0_	1.66 (0.82)	t_0_-t_1_: -0.042	t_0_-t_1:_ 0.2420	.785	.822
	t_1_	1.61 (0.79)	t_0_-t_2_: -0.035	t_0_-t_2:_ 0.2420		.832
	t_2_	1.65 (0.79)	t_1_-t_2_: 0.007	t_1_-t_2:_ 0.7797		.792
Self-blame	t_0_	1.60 (0.75)	t_0_-t_1_: 0.018	t_0_-t_1:_ 0.5657	.571	.704
	t_1_	1.64 (0.74)	t_0_-t_2_: 0.043	t_0_-t_2:_ 0.5550		.696
	t_2_	1.67 (0.79)	t_1_-t_2_: 0.025	t_1_-t_2:_ 0.5657		.757
Self-distraction	t_0_	2.53 (0.74)	t_0_-t_1_: -0.009	t_0_-t_1:_ 0.7840	.453	.507
	t_1_	2.53 (0.70)	t_0_-t_2_: -0.068	t_0_-t_2:_ 0.1173		.502
	t_2_	2.48 (0.67)	t_1_-t_2_: -0.059	t_1_-t_2:_ 0.1173		.436
Substance use	t_0_	1.34 (0.64)	t_0_-t_1_: 0.004	t_0_-t_1:_ 0.8708	.721	.909
	t_1_	1.35 (0.66)	t_0_-t_2_: -0.016	t_0_-t_2:_ 0.7158		.934
	t_2_	1.33 (0.66)	t_1_-t_2_: -0.020	t_1_-t_2:_ 0.7158		.929
Venting	t_0_	2.05 (0.72)	t_0_-t_1_: 0.011	t_0_-t_1:_ 0.7099	.541	.613
	t_1_	2.06 (0.72)	t_0_-t_2_: -0.029	t_0_-t_2:_ 0.5408		.625
	t_2_	2.01 (0.70)	t_1_-t_2_: -0.040	t_1_-t_2:_ 0.5408		.567

*Abbreviations:* *ICC* Intraclass correlation coefficient, *SD* Standard deviation, *p*_*BH*_ Benjamini–Hochberg corrected *p*-value

^a^Linear mixed model specifications: Independent variable = time; dependent variable = Brief COPE responses; Participants were considered subjects. Parameter estimates are based on the restricted maximum likelihood (REML) method. The variance–covariance structure of the random effects was specified as unstructured (UN), the variance–covariance structure of the within-group residuals (time) was specified as first-order autoregressive (AR1)

Irrespective of the time of assessment (Model 1, Supplementary Table 2), *acceptance*, *active coping*, *emotional support*, *humour*, and *positive reframing* had in general a positive effect on PD (negative association). In turn, *behavioural disengagement*, *denial*, *self-blame*, *self-distraction*, and *substance use* had a negative effect on PD (positive association). The *use of informational support*, *planning*, *religion*, and *venting* were not significantly associated with PD.

After the interaction term with the time of measurement was included (Model 2), results indicated a significantly lower effect of *behavioural disengagement* and *denial* on PD at t2 compared to t0. Moreover, *venting* became statistically significant, showing a positive link with PD at baseline. At third measurement *planning* and *venting* were negatively associated with PD. Since the data was positively skewed, the same analysis was conducted after the dependent variable had been logarithmically transformed. Results then indicated a significant interaction for *planning* and *venting* only.

Model 3 was the multivariable model including all coping responses still attaining statistical significance after Benjamini–Hochberg correction was applied. Hence, *use of informational support*, *planning*, *religion*, and *venting* were excluded. Results indicated that *humour* was no longer significantly associated with PD (Model 3, Supplementary Table 3). The highest positive associations (i.e. negative effect) between coping responses and PD were evident for *substance use* and *self-blame*, followed by *denial*, *behavioural disengagement*, and *self-distraction*. Concerning positive effects (i.e. negatively associated) on PD, *active coping* and *emotional support* could be identified as most influential, followed by *positive reframing* and *acceptance*. Similarly to Model 2, Model 4 included the time of measurement as interaction term, which was not statistically significant concerning any coping response. Model 3 was more parsimonious compared to Model 4. Thus, it is primarily used for the discussion of the results.

### Secondary gender and country specific analysis

Comparing men and women did reveal statistically significant differences at baseline for *anger-hostility*, *anxiety*, *somatization* and *global severity*, with higher scores for women (Supplementary Table 9). *Interpersonal sensitivity* was lower in men at all assessments. Both populations, from Tyrol and South-Tyrol did not differ regarding PD responses (Supplementary Table 11).

Men and women differed significantly (i.e. men scored lower) at all measurements concerning the following coping responses: *emotional support*, *informational support*, *venting*. *Active coping* (t2), *religion* (t2 & t3) was higher, and *humour* (t0 & t1), *substance use* (t1 & t2) lower in women, compared to men (Supplementary Table 10). Tyrolean and South-Tyrolean participants differed concerning *emotional support*, *humour*, *religion*, and *substance use* at t0, t1 and t2 (Supplementary Table 12).

### Sensitivity analysis

According to the results by multivariable logistic regression analysis, none of the included predictor variables, i.e. age, gender, residence, relationship status, severe physical illness, and treatment due to MHD, were significant predictors for missing data (Supplementary Table 4). Little’s test of missing completely at random was statistically not significant (χ^2^[1399] = 1328.2, *p* = 0.9110).

**Fig. 2:**
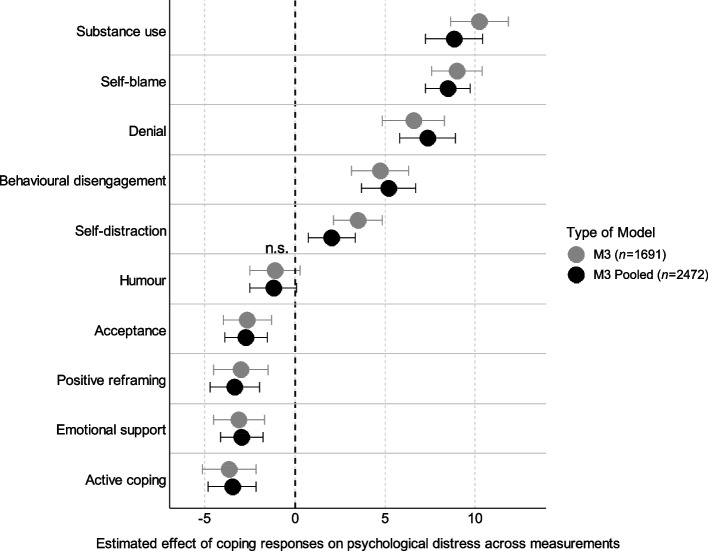
Lollipop plot visualizing the effect of coping responses on psychological distress. Abbreviations. n.s. = not significant at the α = 0.05 level. Note. Plot shows model estimates with 95% confidence intervals. Variable order is based on the estimates’ size (highest positive to highest negative) in the non-pooled Model 3. Positive estimates are associated with increased psychological distress, negative estimates are associated with decreased psychological distress. Included covariates: *gender*, *age*, *residence*. Both models did not include the following coping responses as independent variables due to non-significant results in the univariate analysis: *Informational support*, *planning*, *religion*, and *venting*

The model comparison (non-pooled Model 3 vs. pooled Model 3) showed mostly consistent results in terms of effect size and confidence intervals. Point estimates regarding *self-distraction*, *substance use*, and *denial* showed highest deviations compared to the non-pooled model but were well within the range of the respective confidence intervals, thereby indicating no statistically significant differences between the two models. Furthermore, according to the pooled model point-estimate *positive reframing* had a higher positive impact on PD compared to *emotional support*. Detailed results of the pooled models can be found in the Supplementary Tables 6 and 7. The coping responses’ effects on PD for both models are displayed in Fig. 2.

## Discussion

The aim of our study was to investigate whether PD and coping behaviour changed over the course of the pandemic and to what extent coping responses are related to PD.

Contrary to our hypothesis, the results indicated only marginally decreased overall PD from first to second follow-up measurement. It was found that particularly the proportion of clinically relevant phobic anxiety values subsided.

As assumed, mean-level changes between measurements were only apparent for 3 out of 14 coping responses. *Behavioural disengagement* increased from baseline to subsequent measurements, whereas *positive reframing* and *humour* were slightly lower at follow-up compared to baseline. Differential stability was moderate for most coping responses and high for *humour*, *substance use*, and *religion*.

*Substance use*, *self-blame*, *denial*, *behavioural disengagement*, and *self-distraction* predicted increased PD, whereas *active coping*, *emotional support*, *positive reframing*, and *acceptance*, in turn, were predictors for decreased PD. These associations were stable over time, which was verified by sensitivity analysis. Results are discussed more thoroughly in the subsequent sections.

### Psychological distress in the general population during and after the pandemic

Our analysis yielded decreased PD levels from first to second follow-up measurement. Considering the sample’s average scores in 2020/2021 (29.08), 2022 (31.01), and 2023 (28.70) it becomes evident that this change (effect size: d_t1-t2_ = 0.08) is more statistically significant than clinically relevant.

Although meta-analytic studies predominantly show that mental health deteriorated at the beginning of the pandemic and returned to the pre-pandemic levels over time, these findings are not homogeneous among samples [56, 57]. In fact, variability between subgroups is evident, but it was also highlighted that a major proportion of the population has high resilience capabilities and thus experienced no to very little mental health impairments during the pandemic [58, 59]. This may explainmarginal changes in PD in our sample.

The degree of psychological distress observed in our sample could also partly be explained on a sub-domain level, considering the constructs measured with the BSCL questionnaire. For instance, *somatization* scores were increased at first follow-up and measures among other symptoms e.g. ‘pains in the heart or chest’, ‘trouble getting your breath’, ‘hot and cold spells’, and ‘feeling weak in parts of your body’. Moreover, the obsessive-compulsiveness domain also includes symptoms related to ‘trouble remembering things’, ‘your mind going blank’ or ‘trouble concentrating’. These symptoms are commonly observed in patients during and after a SARS-CoV-2 infection [60]. Given the increasing numbers of individuals reporting on having a COVID-19 infection, it can be assumed that many of the respondents were currently experiencing or have recently experienced at least some extend of these symptoms. Moreover, we observed decreased mean and clinically relevant T-scores between consecutive measurements for *phobic anxiety*, measured by items like ‘Feeling afraid to travel on buses, subways, or trains’ or ‘Feeling uneasy in crowds’. Since their roll-out COVID-19 vaccines were quickly spread among the population with over 90% being vaccinated in our sample. This and the decrease in the life-threatening potential of the virus itself could have contributed to the fact that people's perception of the virus has changed.

Drapeau and colleagues [61] suggests that the prevalence of PD ranges from 5 to 27% in the general population but can reach even higher levels in certain population groupings or when someone is exposed to stressful events and life conditions. For example, some studies found that compared to men, women exhibited increased PD during and after pandemic-associated lockdowns [62, 63]. Yoshioka and colleagues found that, among women, caregiving burden, domestic violence and fear of COVID-19 were independently associated with higher levels of psychological distress [64]. According to a study by Kim and Royle, these periods were also associated with increased rates of domestic violence against women [65]. These findings are somewhat contrary to our results. Although, we found slightly higher overall PD (and on some sub-domains) in women, compared to men at baseline measurement (winter 2020 – spring 2021), exposure to domestic violence was not higher for women between measurements when compared to men.

The pandemic’s repercussions continue to be observed in the economy and labour markets. The tourism and hospitality sector make up much of the (South-) Tyrol’s economic strength and provided employment to almost 20% of study participants. These recovering industries were severely impacted, resulting in a shocked labour market where unemployment rates rose by nearly 100%, from 4.7% to 12.9%; [66, 67]. In line with findings from the US, related job insecurity can be expected to be associated with decreased mental health [68]. In addition, the events of the war in Ukraine exacerbated many of the economic difficulties and caused humanitarian catastrophes that affected people beyond Ukraine's borders [69, 70].

### Variability of coping behaviour

In terms of normative stability, we observed that, with the exception of three responses, coping behaviour remained stable over the three-year survey period. *Behavioural disengagement* marginally increased from baseline to follow-up, *humour* slightly decreased from baseline to first follow-up, and *positive reframing* also slightly decreased between baseline and second follow-up. Although these differences were statistically significant, their magnitude and associated effect sizes were rather unsubstantial, thus favouring a trait-like disposition.

In line with our findings, Nielsen and colleagues [38] have shown that mean-levels of coping were hardly susceptible to change over a 2-year period in a sample of employed Norwegian adults. Next to *positive reframing* they identified small differences for *informational* and *emotional support*, *self-distraction*, and *self-blaming*. Except for *active coping*, *venting*, and *acceptance*, Godor and van der Hallen [37] found similar results in a sample from the Netherlands before and during the COVID-19 pandemic.

Looking at the covariance analysis in our study, at first glance there seems to be a contradiction as we obtained moderate differential stability results for most coping responses, providing arguments in favour of state-like disposition. Yet, previous research also supports these findings [38, 71, 72]. According to a study by Louvet and colleagues [73], this equivocality might be explained by possible subgroup differences that could have balanced each other out, concealing meaningful changes in individuals behind non-significant mean-level results.

Our sample consisted of a broad cross-section of society from North- and South Tyrol, including people with different educational backgrounds, resources, and obligations (e.g., childcare, homeschooling, treatment due to MHD, etc.). Given that coping behaviour is influenced by cognitive appraisals, situational factors, resources, previous experiences, and personal attributes [17], it is conceivable that a mixture of these factors has contributed to the heterogeneity in coping utilization across measurements. For instance, parents of young children could have perceived lockdown-related school closure as uncontrollable, very stressful, and an important event, which possibly led to adapted coping utilization. On the other hand, people without young children may have attached little importance to this restriction and may therefore have been able to maintain their coping strategies. These adults also may have had a harder time adjusting their coping styles during the pandemic, since their pre-pandemic coping strategies likely involved frequent social activities, such as dining out, attending bars, or engaging in other external leisure activities. When these outlets were suddenly unavailable due to restrictions, those without children may have struggled more to adapt, as their typical means of coping were heavily reliant on social and public environments.

In addition, resources such as social support were available to varying degrees in subgroups during the pandemic [74–76], which may have had an impact on susceptibility to situational factors. Interestingly, we observed both mean-level stability and high ICCs in *religion* and *substance use* coping with both responses being used to a low extent. In terms of clinical implications, this suggests that these strategies are far less adaptive in their nature compared to other coping responses.

### Impact of coping behaviour on psychological distress

Our study’s findings highlight a crucial distinction between maladaptive and adaptive coping mechanisms. Positive associations with PD were evident for maladaptive coping strategies such as s*ubstance use*, *self-blame*, *denial*, *behavioural disengagement*, and *self-distraction*. In contrast, adaptive coping strategies like *active coping*, *emotional support*, *positive reframing*, and *acceptance* were negatively associated with PD. Research across 30 countries reported similar associations for maladaptive coping with *self-blame* being the strongest predictor not only for lower mental health, but also for decreased physical health. Furthermore, *humour*, *acceptance*, and *active coping* predicted lower PD, with the latter also associated with increased physical health [31].

Conversely to our findings, *emotional support* was not [77, 78] or even positively [31] linked to PD in studies conducted during the pandemic’s initial phase. The wording used for *emotional support* in the Brief COPE includes statements like ‘I've been getting emotional support from others’, and ‘I've been getting comfort and understanding from someone’. It is possible that the relief experienced through supportive behaviour was intertwined with the level of symptoms and negative experiences already present in participants. Thus, individuals with high levels of distress may perceive this resource as not viable or even burdensome, while participants with lower levels of distress may find it helpful. This may have resulted in a bidirectional association with PD as described in the literature [79].

As previously demonstrated in a study by Avsec and collaborators [80], we also observed that the negative impact of avoidance coping (*behavioural disengagement*, *denial*, *self-blame*, and *substance use*) on PD was consistently higher than the positive impact of approach coping (e.g., *acceptance*, *active coping*, *emotional support*, and *positive reframing*). The latter strategies aim to reduce or eliminate the stressor. The changes and restrictions brought about by the pandemic were primarily the result of decisions not within the control of an individual. As a result, people were able to take only limited action against these restrictions, preventing these coping strategies from being fully effective. Another aspect is limited availability of resources and factors that can facilitate the application of certain coping behaviours. For example, it has been shown that next to social support, psychological flexibility also encourages the application of approach coping [81]. Nevertheless, the potential of coping strategies should not be ignored since positive reappraisal (comparable to *positive reframing*) was shown to be a protective factor with regard to PD during the pandemic [15].

## Limitations and outlook

Our study is distinguished by the examination of coping behaviour and PD in a general population sample over the course of three years. Through sensitivity analysis we were able to confirm the results of the complete case analysis. Nonetheless, there are a few limitations to mention. Most importantly, representativeness of the sample may by impaired as attrition between consecutive measurements was not random. We also fail to answer the question of whether coping behaviour is a trait or a state construct. This question will likely not be answered singularly; rather, it is more probable that both are important to varying degrees [71]. Although we countered some of the criticisms raised by Carver and Connor-Smith [82], like a lack of longitudinal studies, or recording of coping categories that are too broad, further adaptations are necessary. They suggest investigating whether certain coping behaviours are perceived as more or less useful at different times and advocate for prospective studies within a laboratory setting facilitating standardised stressors and including daily surveys. Given that we did not conduct any intervention in our study, we cannot rule out the existence of reciprocal mechanisms of action. Studies suggest that mental health can influence coping behaviour [38]. Through standardized experimental procedures, researchers should be able to better investigate the cause-and-effect mechanisms as well as where differences in stress appraisal of individuals lie. In addition, the stable relationships between coping responses and PD shown in our study can also be taken into account when designing E-Health cognitive behavioural programmes (e.g. [83].

## Supplementary Information

Supplementary Material 1.

## Data Availability

According to the Austrian and Italian law, data sharing requires approvals from the regional Committees for Medical and Health Research Ethics and from the regional Data Protection Officers. The data are therefore not publicly available. The data that support these findings can be provided by TS, Medical University Innsbruck, upon reasonable request.

## References

[1] Lal A, Erondu NA, Heymann DL, Gitahi G, Yates R. Fragmented health systems in COVID-19: rectifying the misalignment between global health security and universal health coverage. Lancet. 2021;397(10268):61–7. 33275906 10.1016/S0140-6736(20)32228-5 PMC7834479

[2] Choi S-Y. Industry volatility and economic uncertainty due to the COVID-19 pandemic: Evidence from wavelet coherence analysis. Financ Res Lett. 2020;37: 101783. 33013239 10.1016/j.frl.2020.101783 PMC7524523

[3] Santomauro DF, Herrera AMM, Shadid J, Zheng P, Ashbaugh C, Pigott DM, et al. Global prevalence and burden of depressive and anxiety disorders in 204 countries and territories in 2020 due to the COVID-19 pandemic. Lancet 2021;398(10312):1700–12. Available from: URL: https://www.sciencedirect.com/science/article/pii/S0140673621021437. 10.1016/S0140-6736(21)02143-7 PMC8500697 34634250

[4] Alzueta E, Podhajsky S, Zhao Q, Tapert SF, Thompson WK, de Zambotti M, et al. Risk for depression tripled during the COVID-19 pandemic in emerging adults followed for the last 8 years. Psychol Med. 2023;53(5):2156–63. 34726149 10.1017/S0033291721004062 PMC10260372

[5] Leung CMC, Ho MK, Bharwani AA, Cogo-Moreira H, Wang Y, Chow MSC, et al. Mental disorders following COVID-19 and other epidemics: a systematic review and meta-analysis. Transl Psychiatry. 2022;12(1):205. 35581186 10.1038/s41398-022-01946-6 PMC9110635

[6] Necho M, Tsehay M, Birkie M, Biset G, Tadesse E. Prevalence of anxiety, depression, and psychological distress among the general population during the COVID-19 pandemic: A systematic review and meta-analysis. Int J Soc Psychiatry. 2021;67(7):892–906. 33794717 10.1177/00207640211003121

[7] Varrasi S, Guerrera CS, Platania GA, Castellano S, Pirrone C, Caponnetto P, et al. Professional quality of life and psychopathological symptoms among first-line healthcare workers facing COVID-19 pandemic: an exploratory study in an Italian southern hospital. Health Psychol Res. 2023;11:67961. 10.52965/001c.67961. 10.52965/001c.67961 PMC9907327 36777810

[8] Daly M, Robinson E. Psychological distress and adaptation to the COVID-19 crisis in the United States. J Psychiatr Res. 2021;136:603–9. 33138985 10.1016/j.jpsychires.2020.10.035 PMC7588823

[9] Ahmed N, Barnett P, Greenburgh A, Pemovska T, Stefanidou T, Lyons N, et al. Mental health in Europe during the COVID-19 pandemic: a systematic review. Lancet Psychiatry. 2023;10(7):537–56. 37321240 10.1016/S2215-0366(23)00113-X PMC10259832

[10] Fancourt D, Steptoe A, Bu F. Trajectories of anxiety and depressive symptoms during enforced isolation due to COVID-19 in England: a longitudinal observational study. Lancet Psychiatry. 2021;8(2):141–9. 33308420 10.1016/S2215-0366(20)30482-X PMC7820109

[11] Prati G, Mancini AD. The psychological impact of COVID-19 pandemic lockdowns: a review and meta-analysis of longitudinal studies and natural experiments. Psychol Med. 2021;51(2):201–11. 33436130 10.1017/S0033291721000015 PMC7844215

[12] Richter D, Riedel-Heller S, Zürcher SJ. Mental health problems in the general population during and after the first lockdown phase due to the SARS-Cov-2 pandemic: rapid review of multi-wave studies. Epidemiol Psychiatr Sci. 2021;30: e27. 33685551 10.1017/S2045796021000160 PMC7985862

[13] Murphy L, Markey K, O’Donnell C, Moloney M, Doody O. The impact of the COVID-19 pandemic and its related restrictions on people with pre-existent mental health conditions: A scoping review. Arch Psychiatr Nurs. 2021;35(4):375–94. 34176579 10.1016/j.apnu.2021.05.002 PMC9759111

[14] Robinson E, Daly M. Explaining the rise and fall of psychological distress during the COVID-19 crisis in the United States: Longitudinal evidence from the Understanding America Study. Br J Health Psychol. 2021;26(2):570–87. 33278066 10.1111/bjhp.12493

[15] Riepenhausen A, Veer IM, Wackerhagen C, Reppmann ZC, Köber G, Ayuso-Mateos JL, et al. Coping with COVID: risk and resilience factors for mental health in a German representative panel study. Psychol Med. 2023;53(9):3897–907. 35301966 10.1017/S0033291722000563 PMC8943230

[16] Miglani M, Upadhyay P, Mahajan R, Mishra BP, Sharma T, Mohan B, et al. Psychological resilience, coping, and distress in admitted patients With COVID-19 infection. Prim Care Companion CNS Disord. 2022;24(3):40843. 10.4088/PCC.21m03230 35522834

[17] Lazarus RS, Folkman S. Stress, Appraisal, and Coping. New York: Springer Publishing Company; 1984.

[18] Armocida B, Formenti B, Ussai S, Palestra F, Missoni E. The Italian health system and the COVID-19 challenge. Lancet Public Health. 2020;5(5): e253. 32220653 10.1016/S2468-2667(20)30074-8 PMC7104094

[19] Grande E, Fedeli U, Pappagallo M, Crialesi R, Marchetti S, Minelli G, et al. Variation in cause-specific mortality rates in Italy during the first wave of the COVID-19 pandemic: a study based on nationwide data. Int J Environ Res Public Health. 2022;19(2):805. 35055627 10.3390/ijerph19020805 PMC8776013

[20] Scortichini M, Schneider Dos Santos R, Donato F de', Sario M de, Michelozzi P, Davoli M et al. Excess mortality during the COVID-19 outbreak in Italy: a two-stage interrupted time-series analysis. Int J Epidemiol. 2021;49(6):1909–17. 10.1093/ije/dyaa169 PMC7665549 33053172

[21] Knabl L, Mitra T, Kimpel J, Rössler A, Volland A, Walser A, et al. High SARS-CoV-2 seroprevalence in children and adults in the Austrian ski resort of Ischgl. Commun Med (Lond). 2021;1(1):4. 10.1038/s43856-021-00007-1. 10.1038/s43856-021-00007-1 PMC8633917 34870284

[22] Gibney E. Whose coronavirus strategy worked best? Scientists hunt most effective policies. Nature. 2020;581(7806):15–6. 32341558 10.1038/d41586-020-01248-1

[23] Simon J, Helter TM, White RG, van der Boor C, Łaszewska A. Impacts of the Covid-19 lockdown and relevant vulnerabilities on capability well-being, mental health and social support: an Austrian survey study. BMC Public Health. 2021;21(1):314. 33557816 10.1186/s12889-021-10351-5 PMC7868863

[24] Tracy EL, Chin B, Lehrer HM, Carroll LW, Buysse DJ, Hall MH. Coping strategies moderate the effect of perceived stress on sleep and health in older adults during the COVID-19 pandemic. Stress Health. 2022;38(4):708–21. 34951930 10.1002/smi.3124 PMC10124294

[25] Miao M, Zheng L, Wen J, Jin S, Gan Y. Coping with coronavirus disease 2019: Relationships between coping strategies, benefit finding and well-being. Stress Health. 2022;38(1):47–56. 34057274 10.1002/smi.3072 PMC8237076

[26] Desie Y, Habtamu K, Asnake M, Gina E, Mequanint T. Coping strategies among Ethiopian migrant returnees who were in quarantine in the time of COVID-19: a center-based cross-sectional study. BMC Psychol. 2021;9(1):192. 34879855 10.1186/s40359-021-00699-z PMC8653623

[27] Fluharty M, Fancourt D. How have people been coping during the COVID-19 pandemic? Patterns and predictors of coping strategies amongst 26,016 UK adults. BMC Psychol. 2021;9(1):107. 34266498 10.1186/s40359-021-00603-9 PMC8280648

[28] Shekriladze I, Javakhishvili N, Butsashvili N, Lortkipanidze M. Anxiety, Worry, Life Satisfaction and Coping During the Acute VS Prolonged Pandemic Stress: Evidence From a Repeated Cross-Sectional Study. Int J Public Health. 2022;67: 1604650. 35719738 10.3389/ijph.2022.1604650 PMC9198216

[29] Götmann A, Bechtoldt MN. Coping with COVID-19 – Longitudinal analysis of coping strategies and the role of trait mindfulness in mental well-being. Pers Individ Dif. 2021;175: 110695. 33531724 10.1016/j.paid.2021.110695 PMC7843110

[30] Rogowska AM, Kuśnierz C, Bokszczanin A. Examining Anxiety, Life Satisfaction, General Health, Stress and Coping Styles During COVID-19 Pandemic in Polish Sample of University Students. Psychol Res Behav Manag. 2020;13:797–811. 33061695 10.2147/PRBM.S266511 PMC7532061

[31] Eisenbeck N, Carreno DF, Wong PTP, Hicks JA, María R-RG, Puga JL, et al. An international study on psychological coping during COVID-19: Towards a meaning-centered coping style. Int J Clin. Health Psychol. 2022;22(1):100256. 10.1016/j.ijchp.2021.100256 PMC8355913 34429729

[32] Rahman MA, Islam SMS, Tungpunkom P, Sultana F, Alif SM, Banik B, et al. COVID-19: Factors associated with psychological distress, fear, and coping strategies among community members across 17 countries. Global Health. 2021;17(1):117. 34598720 10.1186/s12992-021-00768-3 PMC8485312

[33] Hanfstingl B, Gnambs T, Fazekas C, Gölly KI, Matzer F, Tikvić M. The Dimensionality of the Brief COPE before and during the COVID-19 Pandemic. Assessment. 2023;30(2):287–301. 34654329 10.1177/10731911211052483 PMC9902999

[34] Collantoni E, Saieva AM, Meregalli V, Girotto C, Carretta G, Boemo DG, et al. Psychological Distress, Fear of COVID-19, and Resilient Coping Abilities among Healthcare Workers in a Tertiary First-Line Hospital during the Coronavirus Pandemic. J Clin Med. 2021;10(7):1465. 10.3390/jcm10071465. 10.3390/jcm10071465 PMC8038142 33918169

[35] Vagni M, Maiorano T, Giostra V, Pajardi D. Coping With COVID-19: emergency stress, secondary trauma and self-efficacy in healthcare and emergency workers in Italy. Front Psychol. 2020;11: 566912. 33013603 10.3389/fpsyg.2020.566912 PMC7494735

[36] Canestrari C, Bongelli R, Fermani A, Riccioni I, Bertolazzi A, Muzi M, et al. Coronavirus disease stress among Italian healthcare workers: the role of coping humor. Front Psychol. 2020;11: 601574. 33569023 10.3389/fpsyg.2020.601574 PMC7868596

[37] Godor BP, van der Hallen R. Investigating the susceptibility to change of coping and resiliency during COVID-19. Scand J Psychol. 2022;63(3):238–45. 34738232 10.1111/sjop.12787 PMC8662188

[38] Nielsen MB, Knardahl S. Coping strategies: a prospective study of patterns, stability, and relationships with psychological distress. Scand J Psychol. 2014;55(2):142–50. 24697686 10.1111/sjop.12103

[39] Pollak M, Kowarz N, Partheymüller J. Chronology of the Corona Crisis in Austria - Part 4: Lockdowns, mass testing and the launch of the vaccination campaign: University of Vienna - Faculty of Social Sciences; 2021. Available from: URL: https://viecer.univie.ac.at/en/projects-and-cooperations/austrian-corona-panel-project/corona-blog/corona-blog-beitraege/blog100-en/ . Cited 2024 Jan 18.

[40] Carver CS. You want to measure coping but your protocol’s too long: consider the brief COPE. Int J Behav Med. 1997;4(1):92–100. 16250744 10.1207/s15327558ijbm0401_6

[41] Knoll N, Rieckmann N, Schwarzer R. Coping as a mediator between personality and stress outcomes: a longitudinal study with cataract surgery patients. Eur J Pers. 2005;19(3):229–47.

[42] Conti L. Repertorio delle scale di valutazione in psichiatria [Italian collection of the assessment scales in psychiatry]. Firenze: SEE; 2000.

[43] Carver Scheier MF, Weintraub JK. Assessing coping strategies: a theoretically based approach. J Pers Soc Psychol. 1989;56(2):267–83. 2926629 10.1037//0022-3514.56.2.267

[44] Lazarus RS. Psychological stress and the coping process. New York: McGraw-Hill; 1966.

[45] Carver CS, Scheier MF. Attention and Self-Regulation. New York, NY: Springer, New York; 1981.

[46] Scheier MF, Carver CS. A model of behavioral self-regulation: translating intention into action. In: Advances in experimental social psychology, vol. 21. Elsevier; 1988. p. 303–46. (Advances in Experimental Social Psychology).

[47] Franke GH. BSCL: Brief-Symptom-Checklist. 1st ed. Göttingen: Hogrefe; 2017.

[48] Swiss National Association for Quality Development in Hospitals and Clinics. Brief Symptom Checklist BSCL Information on the German, French, Italian an English versions of the BSCL assessment tool.: Swiss National Association for Quality Development in Hospitals and Clinics. 2012. Available from: URL: www.anq.ch/fileadmin/redaktion/sprachneutral/20160426_120611_Info-Instrument_BSCL_EN_final.pdf.

[49] Holzner B, Giesinger JM, Pinggera J, Zugal S, Schöpf F, Oberguggenberger AS, et al. The Computer-based Health Evaluation Software (CHES): a software for electronic patient-reported outcome monitoring. BMC Med Inform Decis Mak. 2012;12: 126. 23140270 10.1186/1472-6947-12-126 PMC3529695

[50] IBM Spss Statistics for Windows. Version version 29. Armonk, NY: IBM Corp; 2022.

[51] R: A language and environment for statistical computing. Vienna, Austria: R Foundation for Statistical; 2023. Available from: URL: https://www.R-project.org/.

[52] nlme: Linear and Nonlinear Mixed Effects Models. Version R package version 3.1–163; 2023. Available from: URL: https://CRAN.R-project.org/package=nlme.

[53] Hadley W. Ggplot2: Elegrant graphics for data analysis. Second edition. Switzerland: Springer; 2016. (Use R!). Available from: URL: https://ggplot2.tidyverse.org.

[54] McDonald RP. Test Theory: A Unified Treatment. 1st ed. London: Taylor and Francis; 1999.

[55] Cohen J. Statistical power analysis for the behavioral sciences. 2nd ed. Hillsdale, N.J.: L. Erlbaum Associates; 1988.

[56] Sun Y, Wu Y, Fan S, Dal Santo T, Li L, Jiang X, et al. Comparison of mental health symptoms before and during the covid-19 pandemic: evidence from a systematic review and meta-analysis of 134 cohorts. BMJ. 2023;380: e074224. 36889797 10.1136/bmj-2022-074224 PMC9992728

[57] Robinson E, Sutin AR, Daly M, Jones A. A systematic review and meta-analysis of longitudinal cohort studies comparing mental health before versus during the COVID-19 pandemic in 2020. J Affect Disord. 2022;296:567–76. 34600966 10.1016/j.jad.2021.09.098 PMC8578001

[58] Shevlin M, Butter S, McBride O, Murphy J, Gibson-Miller J, Hartman TK, et al. Refuting the myth of a “tsunami” of mental ill-health in populations affected by COVID-19: evidence that response to the pandemic is heterogeneous, not homogeneous. Psychol Med. 2023;53(2):429–37. 33875044 10.1017/S0033291721001665 PMC8111207

[59] Shevlin M, Butter S, McBride O, Murphy J, Gibson-Miller J, Hartman TK, et al. Psychological responses to the COVID-19 pandemic are heterogeneous but have stabilised over time: 1 year longitudinal follow-up of the COVID-19 Psychological Research Consortium (C19PRC) study. Psychol Med. 2023;53(7):3245–7. 34538283 10.1017/S0033291721004025 PMC8485012

[60] Ceban F, Ling S, Lui LM, Lee Y, Gill H, Teopiz KM, et al. Fatigue and cognitive impairment in post-COVID-19 syndrome: a systematic review and meta-analysis. Brain Behav Immun. 2021;101:93–135. 34973396 10.1016/j.bbi.2021.12.020 PMC8715665

[61] Drapeau A, Marchand A, Beaulieu-Prevost D. Epidemiology of Psychological Distress. Mental Illnesses -Understanding, Prediction and Control. InTech; 2012. 10.5772/30872.

[62] Tutzer F, Frajo-Apor B, Pardeller S, Plattner B, Chernova A, Haring C, et al. Psychological distress, loneliness, and boredom among the general population of Tyrol, Austria during the COVID-19 Pandemic. Front Psychiatry. 2021;12: 691896. 34177672 10.3389/fpsyt.2021.691896 PMC8222609

[63] Niederkrotenthaler T, Laido Z, Kirchner S, Braun M, Metzler H, Waldhör T, et al. Mental health over nine months during the SARS-CoV2 pandemic: representative cross-sectional survey in twelve waves between april and december 2020 in Austria. J Affect Disord. 2022;296:49–58. 34587549 10.1016/j.jad.2021.08.153 PMC8426850

[64] Yoshioka T, Okubo R, Tabuchi T, Odani S, Shinozaki T, Tsugawa Y. Factors associated with serious psychological distress during the COVID-19 pandemic in Japan: a nationwide cross-sectional internet-based study. BMJ Open. 2021;11(7): e051115. 34226236 10.1136/bmjopen-2021-051115 PMC8260284

[65] Kim B, Royle M. Domestic violence in the context of the COVID-19 Pandemic: A synthesis of systematic reviews. Trauma Violence Abuse. 2024;25(1):476–93. 36847221 10.1177/15248380231155530 PMC9974382

[66] Aschauer W, Egger R. Transformations in tourism following COVID-19? A longitudinal study on the perceptions of tourists. JTF 2023.

[67] Bichler BF, Petry T, Peters M. ‘We did everything we could’: how employees’ made sense of COVID-19 in the tourism and hospitality industry. Curr Issue Tour. 2022;25(23):3766–82.

[68] Obrenovic B, Du J, Godinic D, Baslom MMM, Tsoy D. The Threat of COVID-19 and Job Insecurity Impact on Depression and Anxiety: An Empirical Study in the USA. Front Psychol. 2021;12: 648572. 34484024 10.3389/fpsyg.2021.648572 PMC8411708

[69] Østergaard SD, Rohde C, Jefsen OH. Deterioration of patients with mental disorders in Denmark coinciding with the invasion of Ukraine. Acta Psychiatr Scand. 2022;146(2):107–9. 35575624 10.1111/acps.13440 PMC9545221

[70] Chudzicka-Czupała A, Hapon N, Man RHC, Li D-J, Żywiołek-Szeja M, Karamushka L, et al. Associations between coping strategies and psychological distress among people living in Ukraine, Poland, and Taiwan during the initial stage of the 2022 War in Ukraine. Eur J Psychotraumatol. 2023;14(1): 2163129. 37052087 10.1080/20008066.2022.2163129 PMC9848330

[71] Schwartz JE, Neale J, Marco C, Shiffman SS, Stone AA. Does trait coping exist? A momentary assessment approach to the evaluation of traits. J Pers Soc Psychol. 1999;77(2):360–9. 10474211 10.1037//0022-3514.77.2.360

[72] Todd M, Tennen H, Carney MA, Armeli S, Affleck G. Do we know how we cope? Relating daily coping reports to global and time-limited retrospective assessments. J Pers Soc Psychol. 2004;86(2):310–9. 14769086 10.1037/0022-3514.86.2.310

[73] Louvet B, Gaudreau P, Menaut A, Genty J, Deneuve P. Longitudinal patterns of stability and change in coping across three competitions: a latent class growth analysis. J Sport Exerc Psychol. 2007;29(1):100–17. 17556778 10.1123/jsep.29.1.100

[74] Tutzer F, Schurr T, Frajo-Apor B, Pardeller S, Plattner B, Schmit A, et al. Relevance of spirituality and perceived social support to mental health of people with pre-existing mental health disorders during the COVID-19 pandemic: a longitudinal investigation. Soc Psychiatry Psychiatr Epidemiol. 2024;59(8):1437-48. 10.1007/s00127-023-02590-1. 10.1007/s00127-023-02590-1 PMC11291591 38112803

[75] Lommer K, Schurr T, Frajo-Apor B, Plattner B, Chernova A, Conca A, et al. Addiction in the time of COVID-19: Longitudinal course of substance use, psychological distress, and loneliness among a transnational Tyrolean sample with substance use disorders. Front Psychiatry. 2022;13: 918465. 35982932 10.3389/fpsyt.2022.918465 PMC9380400

[76] Hofer A, Kachel T, Plattner B, Chernova A, Conca A, Fronthaler M, et al. Mental health in individuals with severe mental disorders during the covid-19 pandemic: a longitudinal investigation. Schizophrenia (Heidelb). 2022;8(1):17. 35260590 10.1038/s41537-022-00225-z PMC8903129

[77] Gurvich C, Thomas N, Thomas EH, Hudaib A-R, Sood L, Fabiatos K, et al. Coping styles and mental health in response to societal changes during the COVID-19 pandemic. Int J Soc Psychiatry. 2021;67(5):540–9. 33016171 10.1177/0020764020961790

[78] Shamblaw AL, Rumas RL, Best MW. Coping during the COVID-19 pandemic: Relations with mental health and quality of life. Canadian Psychology / Psychologie canadienne. 2021;62(1):92–100.

[79] Thomas S, Kanske P, Schäfer J, Hummel KV, Trautmann S. Examining bidirectional associations between perceived social support and psychological symptoms in the context of stressful event exposure: a prospective, longitudinal study. BMC Psychiatry. 2022;22(1):736. 36443716 10.1186/s12888-022-04386-0 PMC9703701

[80] Avsec A, Eisenbeck N, Carreno DF, Kocjan GZ, Kavčič T. Coping styles mediate the association between psychological inflexibility and psychological functioning during the COVID-19 pandemic: A crucial role of meaning-centered coping. J Contextual Behav Sci. 2022;26:201–9. 36247215 10.1016/j.jcbs.2022.10.001 PMC9536873

[81] Tindle R, Hemi A, Moustafa AA. Social support, psychological flexibility and coping mediate the association between COVID-19 related stress exposure and psychological distress. Sci Rep. 2022;12(1):8688. 35606392 10.1038/s41598-022-12262-w PMC9126245

[82] Carver CS, Connor-Smith J. Personality and coping. Annu Rev Psychol. 2010;61:679–704. 19572784 10.1146/annurev.psych.093008.100352

[83] Ebert DD, van Daele T, Nordgreen T, Karekla M, Compare A, Zarbo C, et al. Internet- and mobile-based psychological interventions: applications, efficacy, and potential for improving mental health. Eur Psychol. 2018;23(2):167–87.

