# Supplementary material for: Overcoming times of crisis: unveiling coping strategies and mental health in a transnational general population sample during and after the COVID-19 pandemic
Source: BMC Psychol. 2024 Sep 19;12:493. doi: 10.1186/s40359-024-02001-3 (PMC11412033; doi:10.1186/s40359-024-02001-3)
Supplement: Supplementary file 1 — Supplementary Material 1. [file 40359_2024_2001_MOESM1_ESM.docx]

[**Supplementary Table 1.** COVID-19 and treatment related variables 3](#_Toc176521555)

[**Supplementary Table 2.** Fixed effect estimates in the univariate Linear Mixed Model 4](#_Toc176521556)

[**Supplementary Table 3.** Fixed effect estimates in the multivariable Linear Mixed Model 5](#_Toc176521557)

[**Supplementary Figure 1.** Ridgeline plots depicting the participants‘ coping responses score distribution at baseline and follow-up measurements 6](#_Toc176521558)

[**Multiple Imputation & Sensitivity Analysis Procedure** 7](#_Toc176521559)

[**Multiple Imputation & Sensitivity Analysis Results** 7](#_Toc176521560)

[**Supplementary Figure 2.** Pie chart depicting complete and incomplete data with respect to cases and values 8](#_Toc176521561)

[**Supplementary Table 4.** Possible predictors of missing data - results of multivariable logistic regression analysis 9](#_Toc176521562)

[**Supplementary Table 5.** Number of missing data regarding Brief COPE and BSCL at baseline (t0) and follow-ups (t1 & t2) 10](#_Toc176521563)

[**Supplementary Figure 3a.** Line chart depicting the imputed mean values of BSCL and Brief COPE questionnaires as a function of iteration number and imputation number 11](#_Toc176521564)

[**Supplementary Figure 3b.** Line chart depicting the imputed mean values of BSCL and Brief COPE questionnaires as a function of iteration number and imputation number 12](#_Toc176521565)

[**Supplementary Figure 3c.** Line chart depicting the imputed mean values of BSCL and Brief COPE questionnaires as a function of iteration number and imputation number 13](#_Toc176521566)

[**Supplementary Figure 3d.** Line chart depicting the imputed mean values of BSCL and Brief COPE questionnaires as a function of iteration number and imputation number 14](#_Toc176521567)

[**Supplementary Figure 3e.** Line chart depicting the imputed mean values of BSCL and Brief COPE questionnaires as a function of iteration number and imputation number 15](#_Toc176521568)

[**Supplementary Table 6.** Pooled estimates of fixed effects in the univariate Linear Mixed Model after multiple imputation procedure 16](#_Toc176521569)

[**Supplementary Table 7.** Pooled estimates of fixed effects in the multivariable Linear Mixed Model after multiple imputation procedure 17](#_Toc176521570)

[**Supplementary Table 8.** Exposure and propensity to violence separated by men and women 18](#_Toc176521571)

[**Supplementary Table 9.** Means, and standard deviations of BSCL scales at baseline (t0) and follow-up (t1 & t2) for men and women 18](#_Toc176521572)

[**Supplementary Table 10.** Means, and standard deviations of Brief COPE scales at baseline (t0) and follow-up (t1 & t2) for men and women 18](#_Toc176521573)

[**Supplementary Table 11.** Means, and standard deviations of BSCL scales at baseline (t0) and follow-up (t1 & t2) for Tyrol and South-Tyrol 19](#_Toc176521574)

[**Supplementary Table 12.** Means, and standard deviations of Brief COPE scales at baseline (t0) and follow-up (t1 & t2) for Tyrol and South-Tyrol 19](#_Toc176521575)

[**References** 20](#_Toc176521576)

# **Supplementary Table 1.** COVID-19 and treatment related variables

| **Variable** | **Measurement** | **N (%)** | **Statistics**^†^ | **Effect size**^§^ | **p-value** |
| --- | --- | --- | --- | --- | --- |
| Exposed to increased violence since the outbreak of the COVID-19 pandemic | t_0_  t_1_  t_2_ | 19/ 598 (3.2%)  24/ 566 (4.2%)  33/ 527 (6.3%) | 9 / (9+12)  10 / (10+20)  9 / (9+20) | 0.07  0.17  0.19 | t_0_-t_1:_ 0.6642  t_0_-t_2:_ 0.1000  t_1_-t_2:_ 0.0631 |
| Propensity for violence has increased since the outbreak of the COVID-19 pandemic | t_0_  t_1_  t_2_ | 73/ 598 (12.2%)  89/ 566 (15.7%)  98/ 527 (18.6%) | χ^2^(1) = 0.24  χ^2^(1) = 5.96  χ^2^(1) = 3.31 | 0.04  0.15  0.12 | t_0_-t_1:_ 0.6254  t_0_-t_2:_ 0.0152^‡^  t_1_-t_2:_ 0.0690 |
| Severe physical health problems (e.g. diabetes, cancer, etc.) | t_0_  t_1_  t_2_ | 49/ 598 (8.2%)  43/ 566 (7.6%)  49/ 527 (9.3%) | 16 / (16+16)  12 / (12+14)  10 / (10+13) | < 0.01  0.04  0.07 | t_0_-t_1:_ >0.9999  t_0_-t_2:_ 0.8454  t_1_-t_2:_ 0.6783 |
| Current treatment due to mental health disorder | t_0_  t_1_  t_2_ | 44/598 (7.4%)  43/566 (7.6%)  41/527 (7.8%) | 14 / (14+16)  14 / (14+15)  14 / (14+11) | 0.03  0.02  0.06 | t_0_-t_1:_ 0.8551  t_0_-t_2:_ >0.9999  t_1_-t_2:_ 0.6900 |
| Psychological/ psychotherapeutic treatment | t_0_  t_1_  t_2_ | 23/44 (52.3%)  30/43 (69.8%)  31/41 (75.6%) | 1 / (1+1)  3 / (3+1)  2 / (2+1) | < 0.01  0.25  0.17 | t_0_-t_1:_ >0.9999  t_0_-t_2:_ 0.6254  t_1_-t_2:_ >0.9999 |
| Psychiatric treatment (outside a hospital) | t_0_  t_1_  t_2_ | 19/44 (43.2%)  22/43 (51.2%)  18/41 (43.9%) | 1 / (1+2)  1 / (1+2)  1 / (1+3) | 0.17  0.17  0.25 | t_0_-t_1:_ >0.9999  t_0_-t_2:_ >0.9999  t_1_-t_2:_ 0.6254 |
| Psychiatric treatment (outpatient unit of a hospital) | t_0_  t_1_  t_2_ | 3/44 (6.8%)  6/43 (14.0%)  5/41 (12.2%) | 0 / (0+1)  0 / (0+1)  1 / (1+1) | ~ 0.50  ~ 0.50  < 0.01 | t_0_-t_1:_ >0.9999  t_0_-t_2:_ >0.9999  t_1_-t_2:_ >0.9999 |
| General practitioner | t_0_  t_1_  t_2_ | 8/44 (18.2%)  3/43 (7.0%)  7/41 (17.1%) | 0 / (0+3)  4 / (4+1)  5 / (5+0) | ~ 0.50  0.30  ~ 0.50 | t_0_-t_1:_ 0.2500  t_0_-t_2:_ 0.3753  t_1_-t_2:_ 0.0631 |
| Care facility (work) | t_0_  t_1_  t_2_ | 0/44 (-)  2/43 (4.7%)  1/41 (2.4%) | - | - | - |
| Care facility (living) | t_0_  t_1_  t_2_ | 1/44 (2.3%)  0/43 (-)  0/41 (-) | - | - | - |

^†^ For the McNemar test a χ^2^ distribution was used, when the number of observations was at least *n*=35. Else the binomial distribution was used and the cell counts for the 2x2 table in the form of (b/(b + c)) were reported.
^‡^ Remains statistically significant (α=0.05) after Benjamini-Hochberg correction
^§^ Cohen’s *g* is calculated with |(b/(b + c)) – 0.5|

# **Supplementary Table 2.** Fixed effect estimates in the univariate Linear Mixed Model

| **Model** | **Coping response** | **Estimate** | **S.E.** | **df** | **t** | **95% CI (Estimate)** | | **p-value** |
| --- | --- | --- | --- | --- | --- | --- | --- | --- |
|  |  |  |  |  |  | **LB** | **UB** |  |
| M1.1 | Acceptance | -5.174 | 0.730 | 871 | -7.084 | -6.608 | -3.741 | <0.0001 |
| M2.1 | Acceptance (t0) | -4.637 | 1.034 | 867 | -4.454 | -6.667 | -2.607 | <0.0001 |
|  | Acceptance (t1 vs. t0) | -0.818 | 1.262 | 867 | -0.648 | -3.296 | 1.659 | 0.5169 |
|  | Acceptance (t2 vs. t0) | -0.612 | 1.282 | 867 | -0.477 | -3.128 | 1.904 | 0.6333 |
| M1.2 | Active coping | -3.999 | 0.777 | 871 | -5.147 | -5.524 | -2.474 | <0.0001 |
| M2.2 | Active coping (t0) | -2.970 | 1.091 | 867 | -2.723 | -5.110 | -0.829 | 0.0066 |
|  | Active coping (t1 vs. t0) | -0.528 | 1.347 | 867 | -0.392 | -3.172 | 2.116 | 0.6952 |
|  | Active coping (t2 vs. t0) | -2.360 | 1.426 | 867 | -1.655 | -5.158 | 0.438 | 0.0983 |
| M1.3 | Behavioural disengagement | 9.008 | 0.874 | 871 | 10.302 | 7.292 | 10.724 | <0.0001 |
| M2.3 | Behavioural disengagement (t0) | 11.308 | 1.338 | 867 | 8.450 | 8.681 | 13.935 | <0.0001 |
|  | Behavioural disengagement (t1 vs. t0) | -2.275 | 1.626 | 867 | -1.399 | -5.465 | 0.916 | 0.1621 |
|  | Behavioural disengagement (t2 vs. t0) | -3.811 | 1.701 | 867 | -2.240 | -7.150 | -0.420 | 0.0253 |
| M1.4 | Denial | 12.647 | 0.902 | 871 | 14.021 | 10.876 | 14.417 | <0.0001 |
| M2.4 | Denial (t0) | 14.031 | 1.281 | 867 | 10.951 | 11.517 | 16.546 | <0.0001 |
|  | Denial (t1 vs. t0) | -0.191 | 1.618 | 867 | -0.118 | -3.366 | 2.985 | 0.9063 |
|  | Denial (t2 vs. t0) | -4.166 | 1.637 | 867 | -2.545 | -7.379 | -0.953 | 0.0111 |
| M1.5 | Emotional support | -3.378 | 0.775 | 871 | -4.361 | -4.899 | -1.858 | <0.0001 |
| M2.5 | Emotional support (t0) | -2.899 | 1.048 | 867 | -2.767 | -4.955 | -0.842 | 0.0058 |
|  | Emotional support (t1 vs. t0) | -0.171 | 1.231 | 867 | -0.139 | -2.586 | 2.245 | 0.8896 |
|  | Emotional support (t2 vs. t0) | -1.432 | 1.292 | 867 | -1.108 | -3.968 | 1.120 | 0.2680 |
| M1.6 | Humour | -3.217 | 0.782 | 871 | -4.115 | -4.751 | -1.683 | <0.0001 |
| M2.6 | Humour (t0) | -3.861 | 1.024 | 867 | -3.771 | -5.871 | -1.851 | 0.0002 |
|  | Humour (t1 vs. t0) | 2.074 | 1.221 | 867 | 1.698 | -0.323 | 4.472 | 0.0898 |
|  | Humour (t2 vs. t0) | 0.650 | 1.224 | 867 | 0.531 | -1.753 | 3.053 | 0.5957 |
| M1.7 | Informational support | -1.104 | 0.745 | 871 | -1.482 | -2.566 | 0.358 | 0.1386 |
| M2.7 | Informational support (t0) | -0.030 | 1.009 | 867 | -0.030 | -2.011 | 1.951 | 0.9762 |
|  | Informational support (t1 vs. t0) | -0.902 | 1.204 | 867 | -0.750 | -3.266 | 1.460 | 0.4536 |
|  | Informational support (t2 vs. t0) | -2.365 | 1.279 | 867 | -1.849 | -4.875 | 0.144 | 0.0647 |
| M1.8 | Planning | 0.113 | 0.786 | 871 | 0.144 | -1.429 | 1.655 | 0.8856 |
| M2.8 | Planning (t0) | 0.775 | 1.102 | 867 | 0.703 | -1.388 | 2.938 | 0.4821 |
|  | Planning (t1 vs. t0) | 0.709 | 1.381 | 867 | 0.513 | -2.001 | 3.419 | 0.6079 |
|  | Planning (t2 vs. t0) | -2.922 | 1.400 | 867 | -2.086 | -5.670 | -0.1733 | 0.0372 |
| M1.9 | Positive reframing | -6.692 | 0.759 | 871 | -8.816 | -8.181 | -5.202 | <0.0001 |
| M2.9 | Positive reframing (t0) | -6.673 | 1.021 | 867 | -6.539 | -8.676 | -4.670 | <0.0001 |
|  | Positive reframing (t1 vs. t0) | 0.608 | 1.259 | 867 | 0.483 | -1.863 | 3.079 | 0.6293 |
|  | Positive reframing (t2 vs. t0) | -1.060 | 1.301 | 867 | -0.815 | -3.613 | 1.494 | 0.4155 |
| M1.10 | Religion | -0.136 | 0.910 | 871 | -0.149 | -1.921 | 1.650 | 0.8816 |
| M2.10 | Religion (t0) | -0.921 | 1.091 | 867 | -0.844 | -3.061 | 1.220 | 0.3988 |
|  | Religion (t1 vs. t0) | 1.247 | 1.129 | 867 | 1.105 | -0.968 | 3.462 | 0.2695 |
|  | Religion (t2 vs. t0) | 1.455 | 1.205 | 867 | 1.208 | -0.909 | 3.819 | 0.2274 |
| M1.11 | Self-blame | 12.185 | 0.747 | 871 | 16.304 | 10.718 | 13.652 | <0.0001 |
| M2.11 | Self-blame (t0) | 13.302 | 1.028 | 867 | 12.946 | 11.286 | 15.319 | <0.0001 |
|  | Self-blame (t1 vs. t0) | -1.448 | 1.242 | 867 | -1.166 | -3.885 | 0.989 | 0.2438 |
|  | Self-blame (t2 vs. t0) | -1.909 | 1.259 | 867 | -1.516 | -4.381 | 0.562 | 0.1299 |
| M1.12 | Self-distraction | 2.059 | 0.784 | 871 | 2.625 | 0.520 | 3.599 | 0.0088 |
| M2.12 | Self-distraction (t0) | 2.139 | 1.060 | 867 | 2.019 | 0.059 | 4.219 | 0.0438 |
|  | Self-distraction (t1 vs. t0) | 0.964 | 1.368 | 867 | 0.705 | -1.721 | 3.649 | 0.4812 |
|  | Self-distraction (t2 vs. t0) | -1.895 | 1.431 | 867 | -1.325 | -4.704 | 0.913 | 0.1857 |
| M1.13 | Substance use | 15.221 | 0.990 | 871 | 15.376 | 13.278 | 17.164 | <0.0001 |
| M2.13 | Substance use (t0) | 14.588 | 1.239 | 867 | 11.770 | 12.155 | 17.021 | <0.0001 |
|  | Substance use (t1 vs. t0) | 1.907 | 1.319 | 867 | 1.446 | -0.681 | 4.495 | 0.1485 |
|  | Substance use (t2 vs. t0) | -0.380 | 1.378 | 867 | -0.276 | -3.084 | 2.324 | 0.7828 |
| M1.14 | Venting | 1.433 | 0.823 | 871 | 1.741 | -0.182 | 3.047 | 0.0820 |
| M2.14 | Venting (t0) | 3.157 | 1.118 | 867 | 2.823 | 0.962 | 5.352 | 0.0049 |
|  | Venting (t1 vs. t0) | -1.649 | 1.343 | 867 | -1.227 | -4.285 | 0.988 | 0.2200 |
|  | Venting (t2 vs. t0) | -4.050 | 1.411 | 867 | -2.872 | -6.819 | -1.282 | 0.0042 |

Abbreviations. CI=confidence interval; S.E.=standard error; df=degree of freedom; LB=lower bound; UB=upper bound; M1=univariate model, including each coping response separately; M2=univariate model, including the interaction with time and each coping response separately

Linear mixed model specifications: Independent variable = coping response; dependent variable = psychological distress; covariates = age, gender, residence;

Participants were considered subjects, time of assessment as factor. Parameter estimates are based on the restricted maximum likelihood (REML) method. The variance-covariance structure of the random effects was specified as unstructured (UN), the variance-covariance structure of the within-group residuals (time) was specified as first-order autoregressive (AR1)

# **Supplementary Table 3.** Fixed effect estimates in the multivariable Linear Mixed Model

| **Model** | **Coping response** | **Estimate** | **S.E.** | **df** | **t** | **95% CI (Estimate)** | | **p-value** |
| --- | --- | --- | --- | --- | --- | --- | --- | --- |
|  |  |  |  |  |  | **LB** | **UB** |  |
| M3 | Acceptance | -2.636 | 0.680 | 862 | -3.875 | -3.972 | -1.301 | 0.0001 |
| M4 | Acceptance (t0) | -2.126 | 1.020 | 840 | -2.084 | -4.129 | -0.124 | 0.0374 |
|  | Acceptance (t1 vs. t0) | 1.172 | 1.305 | 840 | 0.898 | -1.386 | 3.730 | 0.3696 |
|  | Acceptance (t2 vs. t0) | 0.252 | 1.350 | 840 | 0.187 | -2.398 | 2.902 | 0.8520 |
| M3 | Active coping | -3.650 | 0.755 | 862 | -4.834 | -5.132 | -2.168 | <0.0001 |
| M4 | Active coping (t0) | -3.465 | 1.172 | 840 | -2.955 | -5.766 | -1.164 | 0.0032 |
|  | Active coping (t1 vs. t0) | 1.162 | 1.561 | 840 | 0.745 | -1.901 | 4.225 | 0.4566 |
|  | Active coping (t2 vs. t0) | -0.826 | 1.603 | 840 | -0.515 | -3.972 | 2.320 | 0.6064 |
| M3 | Behavioural disengagement | 4.715 | 0.804 | 862 | 5.863 | 3.137 | 6.294 | <0.0001 |
| M4 | Behavioural disengagement (t0) | 5.963 | 1.310 | 840 | 4.55 | 3.392 | 8.535 | <0.0001 |
|  | Behavioural disengagement (t1 vs. t0) | 2.066 | 1.663 | 840 | 1.242 | -1.193 | 5.325 | 0.2145 |
|  | Behavioural disengagement (t2 vs. t0) | -0.922 | 1.710 | 840 | -0.539 | -4.278 | 2.434 | 0.5898 |
| M3 | Denial | 6.577 | 0.877 | 862 | 7.503 | 4.857 | 8.297 | <0.0001 |
| M4 | Denial (t0) | 7.291 | 1.365 | 840 | 5.339 | 4.611 | 9.971 | 0.0001 |
|  | Denial (t1 vs. t0) | 1.203 | 1.779 | 840 | 0.676 | -2.289 | 4.696 | 0.4991 |
|  | Denial (t2 vs. t0) | -3.533 | 1.829 | 840 | -1.932 | -7.123 | 0.056 | 0.0537 |
| M3 | Emotional support | -3.106 | 0.717 | 862 | -4.329 | -4.514 | -1.698 | <0.0001 |
| M4 | Emotional support (t0) | -3.413 | 1.020 | 840 | 3.346 | -5.415 | -1.411 | 0.0009 |
|  | Emotional support (t1 vs. t0) | 0.481 | 1.236 | 840 | 0.390 | -1.944 | 2.907 | 0.6969 |
|  | Emotional support (t2 vs. t0) | -0.178 | 1.318 | 840 | -0.135 | -2.765 | 2.409 | 0.8925 |
| M3 | Humour | -1.105 | 0.710 | 862 | -1.557 | -2.499 | 0.288 | 0.1199 |
| M4 | Humour (t0) | -1.338 | 0.967 | 840 | -1.383 | -3.237 | 0.561 | 0.1671 |
|  | Humour (t1 vs. t0) | 0.965 | 1.196 | 840 | 0.806 | -1.383 | 3.313 | 0.4203 |
|  | Humour (t2 vs. t0) | 0.305 | 1.244 | 840 | 0.245 | -2.137 | 2.747 | 0.8063 |
| M3 | Positive reframing | -3.008 | 0.765 | 862 | -3.932 | -4.509 | -1.507 | 0.0001 |
| M4 | Positive reframing (t0) | -3.008 | 1.112 | 840 | 2.704 | -5.191 | -0.825 | 0.0070 |
|  | Positive reframing (t1 vs. t0) | -0.004 | 1.506 | 840 | -0.002 | -2.959 | 2.952 | 0.9980 |
|  | Positive reframing (t2 vs. t0) | -1.042 | 1.557 | 840 | -0.670 | -4.098 | 2.010 | 0.5033 |
| M3 | Self-blame | 8.987 | 0.715 | 862 | 12.569 | 7.583 | 10.390 | <0.0001 |
| M4 | Self-blame (t0) | 9.317 | 1.044 | 840 | 8.921 | 7.267 | 11.367 | <0.0001 |
|  | Self-blame (t1 vs. t0) | -1.021 | 1.325 | 840 | -0.771 | -3.623 | 1.580 | 0.4410 |
|  | Self-blame (t2 vs. t0) | 0.416 | 1.326 | 840 | 0.314 | -2.186 | 3.019 | 0.7536 |
| M3 | Self-distraction | 3.487 | 0.688 | 862 | 5.068 | 2.139 | 4.835 | <0.0001 |
| M4 | Self-distraction (t0) | 4.399 | 1.082 | 840 | 4.065 | 2.275 | 6.523 | 0.0001 |
|  | Self-distraction (t1 vs. t0) | -1.389 | 1.402 | 840 | 0.990 | -4.142 | 1.364 | 0.3222 |
|  | Self-distraction (t2 vs. t0) | -2.133 | 1.484 | 840 | 1.437 | -5.047 | 0.780 | 0.1510 |
| M3 | Substance use | 10.239 | 0.810 | 862 | 12.641 | 8.651 | 11.827 | <0.0001 |
| M4 | Substance use (t0) | 9.745 | 1.203 | 840 | 8.101 | 7.384 | 12.107 | <0.0001 |
|  | Substance use (t1 vs. t0) | 1.156 | 1.391 | 840 | 0.832 | -1.573 | 3.886 | 0.4059 |
|  | Substance use (t2 vs. t0) | 0.191 | 1.424 | 840 | 0.134 | -2.600 | 2.982 | 0.8932 |

Abbreviations. CI=confidence interval; S.E.=standard error; df=degree of freedom; LB=lower bound; UB=upper bound; M3=multivariable model, including the combined effect of coping responses; M4=multivariable model, including the interaction with time and coping responses

Linear mixed model specifications: independent variable = coping responses; dependent variable = psychological distress; covariates: age, gender, residence;

Participants were considered subjects, time of assessment as factor. Parameter estimates are based on the restricted maximum likelihood (REML) method. The variance-covariance structure of the random effects was specified as unstructured (UN), the variance-covariance structure of the within-group residuals (time) was specified as first-order autoregressive (AR1)

Note. In both models the following independent variables were excluded due to non-significant results in the univariate analysis: *Informational support*, *planning*, *religion*, and *venting*


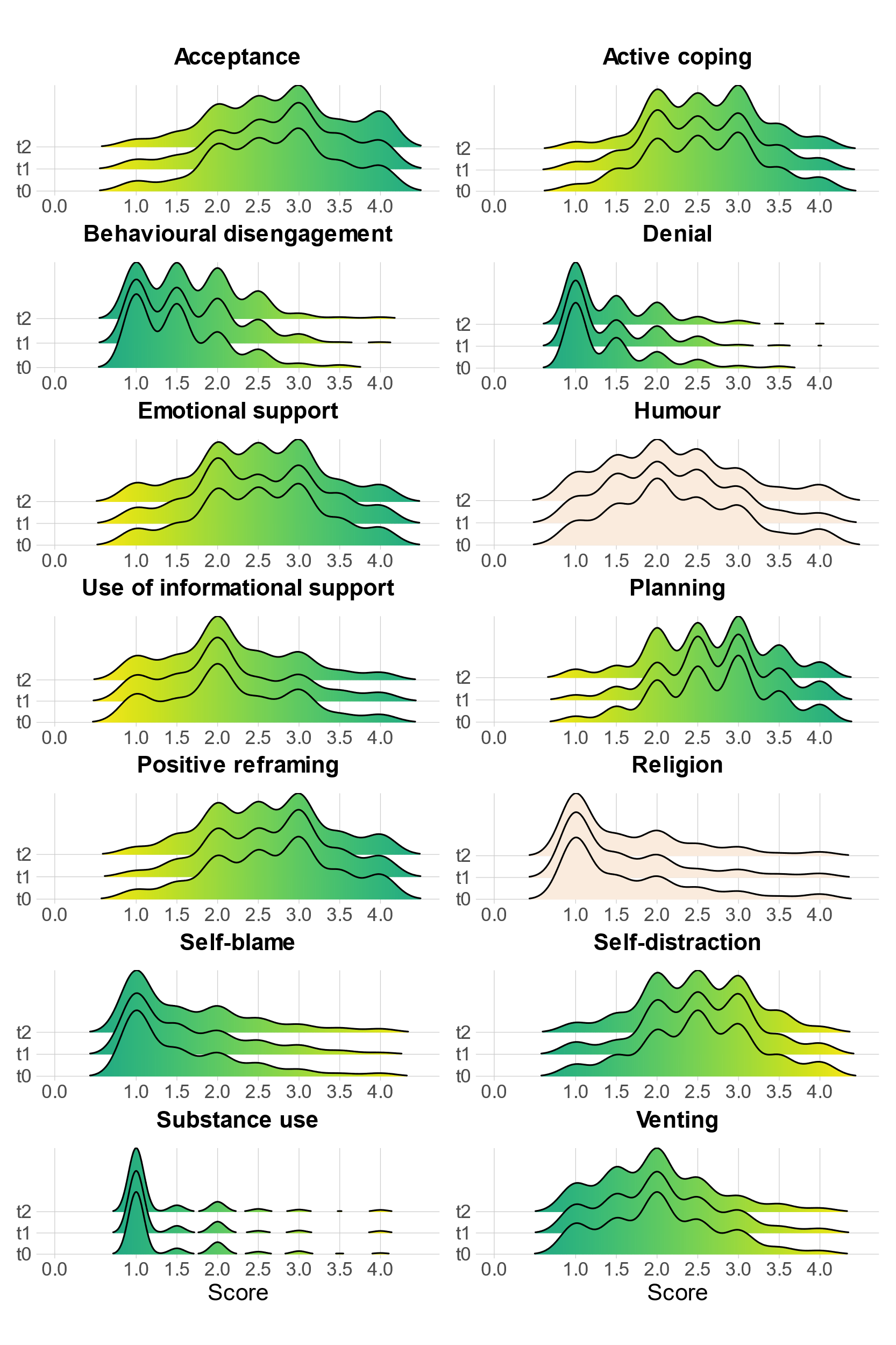


**Supplementary Figure 1.** Ridgeline plots depicting the participants‘ coping responses score distribution at baseline and follow-up measurements
Abbreviations. t0 = Baseline measurement; t1, t2 = Follow-up measurement
Note. The colour gradient indicates in which direction (higher or lower scores) the coping response must be pronounced to be considered favourable or unfavourable. Green colour represents favourable coping, whereas yellow colour can be considered as rather unfavourable coping. *Humour* and *religion* are considered neutral coping styles.

# **Multiple Imputation & Sensitivity Analysis Procedure**

To evaluate the robustness of the main analysis’ results, we compared the primary sample (*n*=1691) with the sample that underwent multiple imputation for missing data (*n*=2472). Initially, a multivariable logistic regression has been conducted, including age, gender, residence, relationship status, severe physical illness and treatment due to mental health disorder as independent variables and information about missing / non-missing data as dependent variable. Next, Little’s MCAR test (Little, 1988) was used to analyse if the data is missing completely at random.

Thereafter, the fully conditional specification (FCS) technique has been employed. This iterative Markov chain Monte Carlo (MCMC) method uses a series of conditional imputation models to specify the multivariate model. The advantage of this approach is that no joint distributional assumptions have to be made (van Buuren, 2007, 2018). For metric variables, predictive mean matching (PMM) was selected as the imputation model type, ensuring that missing values are imputed reasonably. This is done by using complete cases whose predicted mean values are closest to the predicted value for the incomplete case. Thus, if the normality assumption is violated, as in the case of skewed data, PMM might be more suitable than the standard linear regression approach (Horton & Lipsitz, 2001).

The predictor variables incorporated in the analysis encompassed age, gender, relationship status, residence, severe physical illness, treatment due to mental health disorder, and available Brief COPE and BSCL questionnaire scores. Missing values were imputed into the latter both. The minimum and maximum value for the imputed numbers was specified according to the potential scale range of the respective variable. The number of maximum parameters and case draws was set to 10,000. For practical model building purposes the initial number of imputations was established at *m* = 5, hereafter incrementally increasing to *m* = 30, which approximately represented the average percentage of missing values in our dataset (von Hippel, 2009). The maximum number of iterations per imputation was capped at 100.

To ascertain successful convergence of the imputation model, a visual inspection was performed. This involved plotting (line chart) the number of iterations against means of the imputed data. Convergence was evidenced by the absence of distinct trends or divergent streams. Imputed data validity was evaluated through graphical presentations and descriptive statistics. Following Rubin's rules (Rubin, 1987), the data underwent separate analyses and was subsequently pooled to generate final estimates.

# **Multiple Imputation & Sensitivity Analysis Results**

Initially, variables that had undergone the multiple imputation procedure, were analyzed for missingness. Supplementary Figure 2 shows the incomplete data when split according to cases and values. The number of missing data within the coping responses and psychological distress variables is depicted in the Supplementary Table 5. Across all measurements, on average 30% of the values within the Brief COPE and BSCL questionnaire are missing. In total, 61% of all cases had missing data.

According to the results of the multivariable logistic regression, none of the included variables predicted missingness (Supplementary Table 4). Results of the conducted Little’s MCAR was not statistically significant (χ^2^=1328.2, df= 1399, p=0.9110).

Starting with converging five imputations, the multiple imputation procedure could steadily be expanded up to the planned 30 imputations. Successful convergence of the imputation models was checked by line charts, plotting the number of iterations against the mean values of the imputed data (Supplementary Figure 3a-e). Regarding psychological distress, convergence was obtained after 5-10 iterations, meaning there was no separation of streams or definite trend. Similar results could be observed concerning the imputation models for the coping responses.

Based on the data sets generated by multiple imputation (data entries: *n*=2472), pooled estimates for the linear mixed models could then be derived (Supplementary Table 6 and 7) and compared to the models with complete data (data entries: *n*=1691).

# **Supplementary Figure 2.** Pie chart depicting complete and incomplete data with respect to cases and values


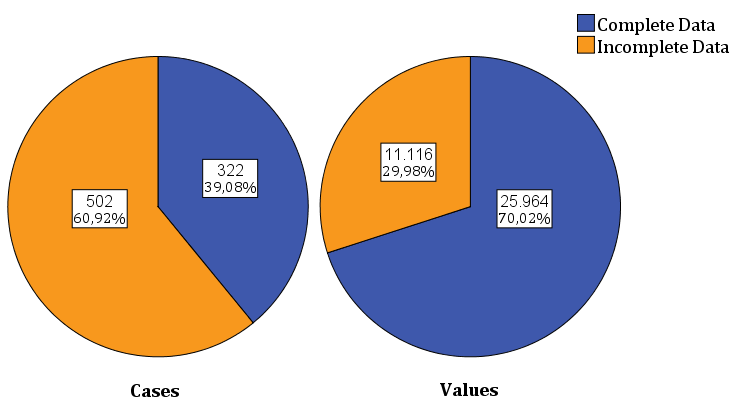


# **Supplementary Table 4.** Possible predictors of missing data - results of multivariable logistic regression analysis

Abbreviations. df= degree of freedom; S.E. = standard error; OR = odds ratio

# **Supplementary Table 5.** Number of missing data regarding Brief COPE and BSCL at baseline (t0) and follow-ups (t1 & t2)

| **Variable** | **Measurement** | **Missing N (%)** |
| --- | --- | --- |
| COPE - Acceptance | t_0_  t_1_  t_2_ | 208/824 (25.2%)  244/824 (29.6%)  288/824 (35.0%) |
| COPE - Active coping | t_0_  t_1_  t_2_ | 206/824 (25.0%)  242/824 (29.4%)  289/824 (35.1%) |
| COPE - Behavioural disengagement | t_0_  t_1_  t_2_ | 210/824 (25.5%)  243/824 (29.5%)  289/824 (35.1%) |
| COPE - Denial | t_0_  t_1_  t_2_ | 210/824 (25.5%)  245/824 (29.7%)  287/824 (34.8%) |
| COPE - Emotional support | t_0_  t_1_  t_2_ | 209/824 (25.4%)  244/824 (29.6%)  288/824 (35.0%) |
| COPE - Humour | t_0_  t_1_  t_2_ | 208/824 (25.2%)  244/824 (29.6%)  288/824 (35.0%) |
| COPE - Informational support | t_0_  t_1_  t_2_ | 208/824 (25.2%)  244/824 (29.6%)  288/824 (35.0%) |
| COPE - Planning | t_0_  t_1_  t_2_ | 210/824 (25.5%)  245/824 (29.7%)  288/824 (35.0%) |
| COPE - Positive reframing | t_0_  t_1_  t_2_ | 210/824 (25.5%)  245/824 (29.7%)  287/824 (34.8%) |
| COPE - Religion | t_0_  t_1_  t_2_ | 209/824 (25.4%)  244/824 (29.6%)  289/824 (35.1%) |
| COPE - Self-blame | t_0_  t_1_  t_2_ | 209/824 (25.4%)  243/824 (29.5%)  288/824 (35.0%) |
| COPE - Self-distraction | t_0_  t_1_  t_2_ | 207/824 (25.1%)  243/824 (29.5%)  289/824 (35.1%) |
| COPE - Substance use | t_0_  t_1_  t_2_ | 206/824 (25.0%)  242/824 (29.4%)  285/824 (34.6%) |
| COPE - Venting | t_0_  t_1_  t_2_ | 212/824 (25.7%)  246/824 (29.9%)  289/824 (35.1%) |
| BSCL - Global Severity Index | t_0_  t_1_  t_2_ | 213/824 (25.8%)  247/824 (30.0%)  288/824 (35.0%) |

# **Supplementary Figure 3a.** Line chart depicting the imputed mean values of BSCL and Brief COPE questionnaires as a function of iteration number and imputation number


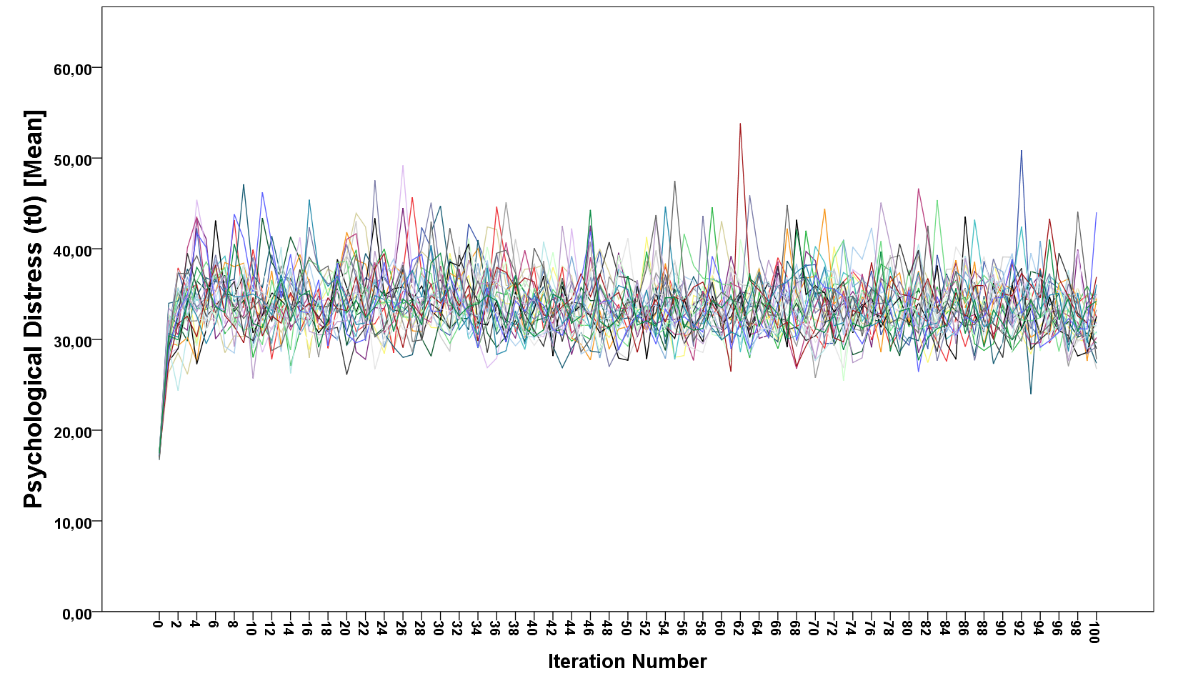

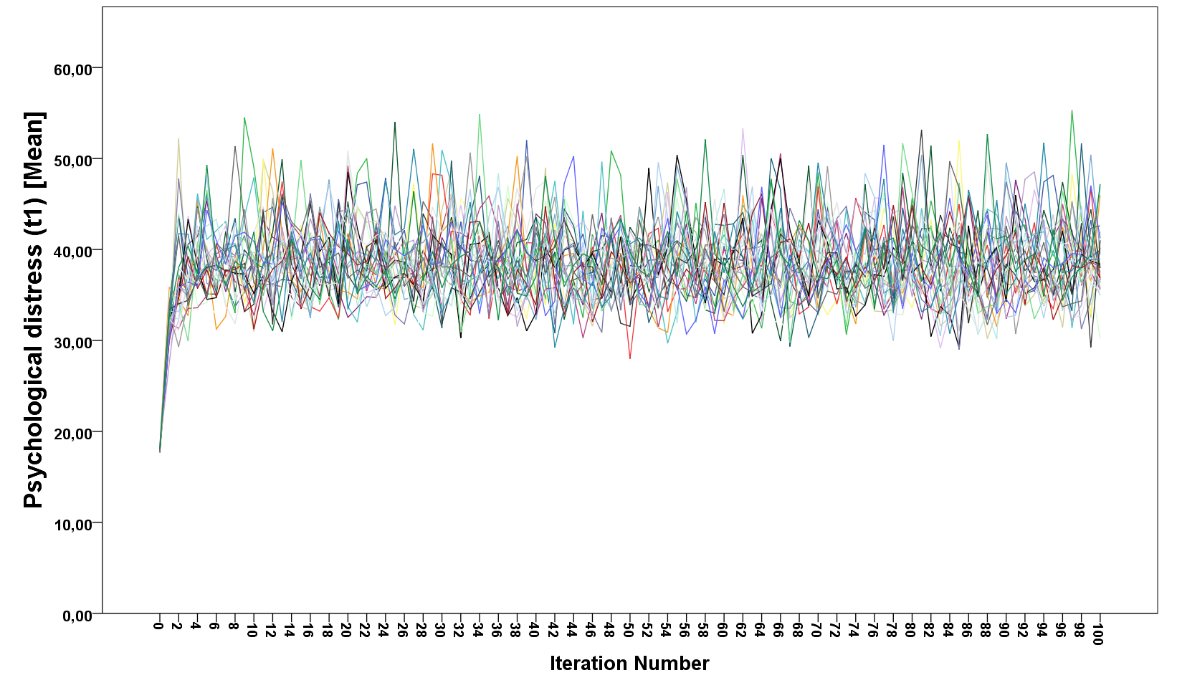

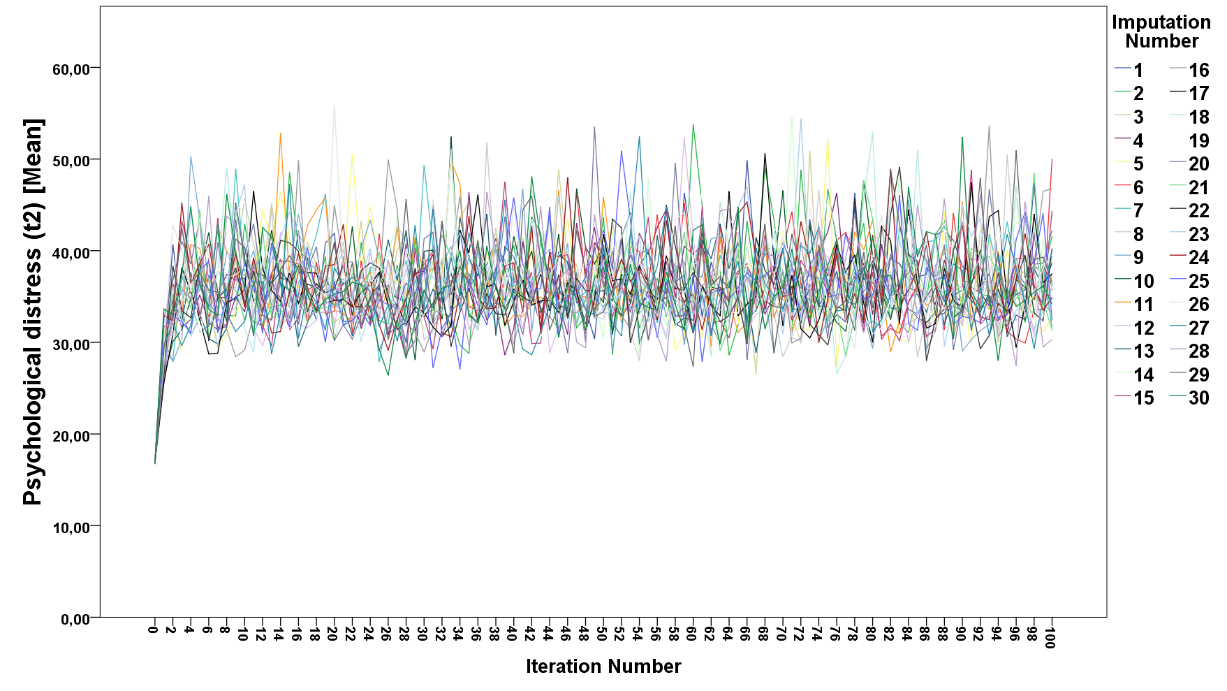


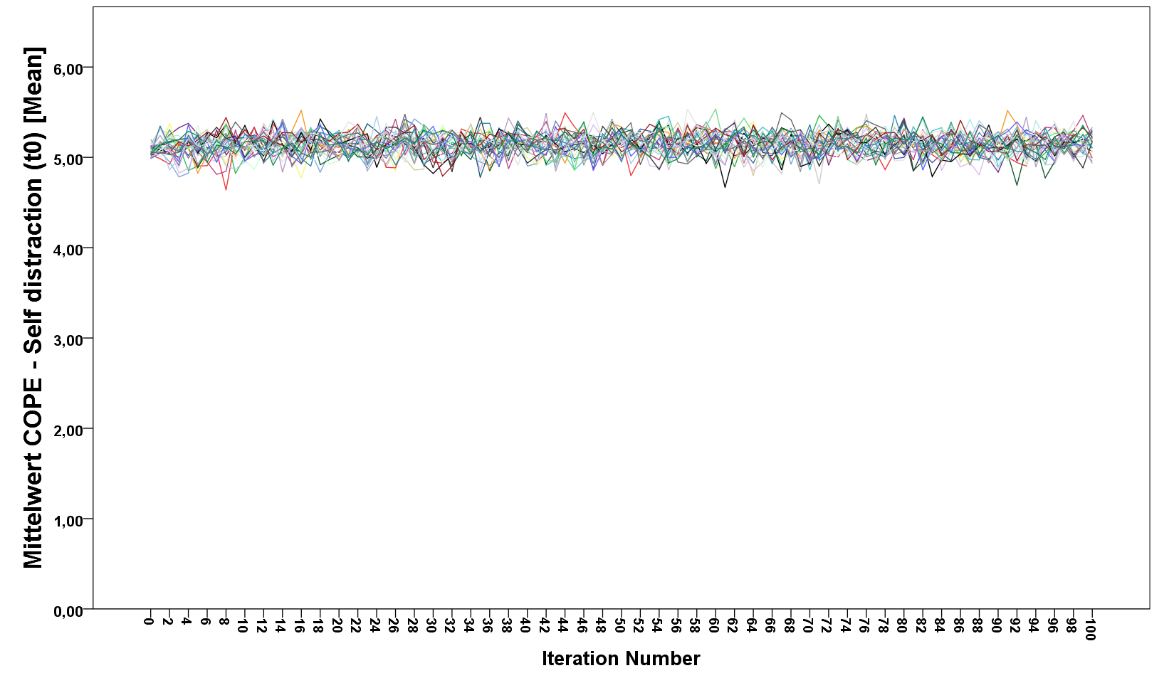

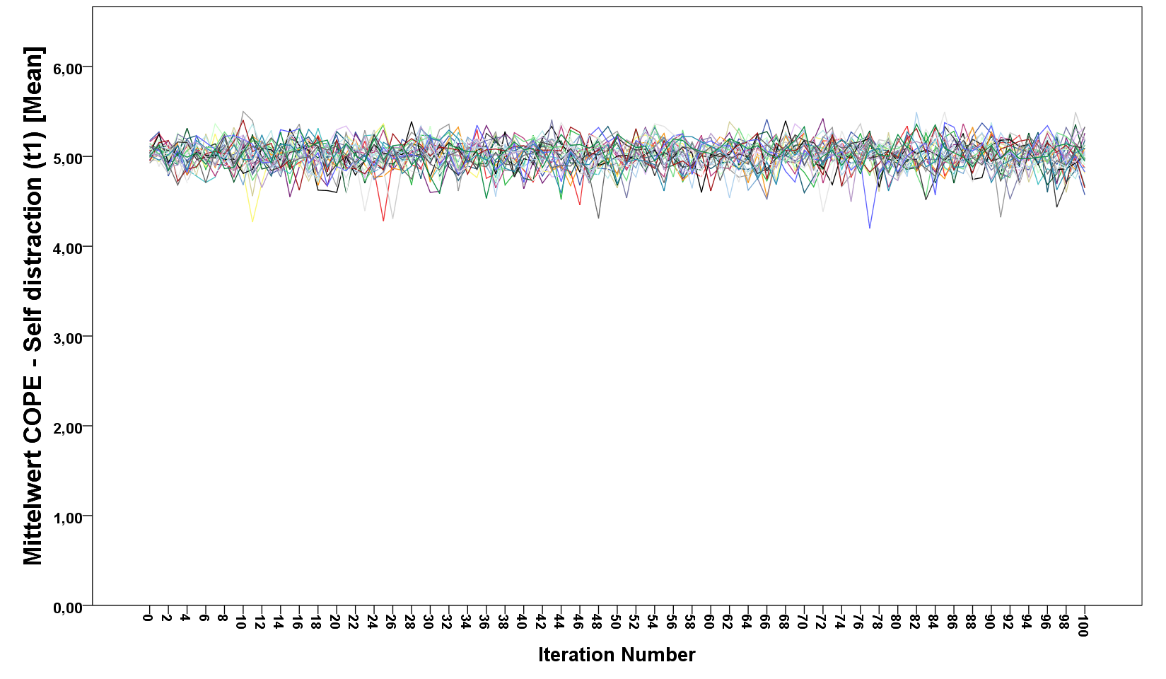

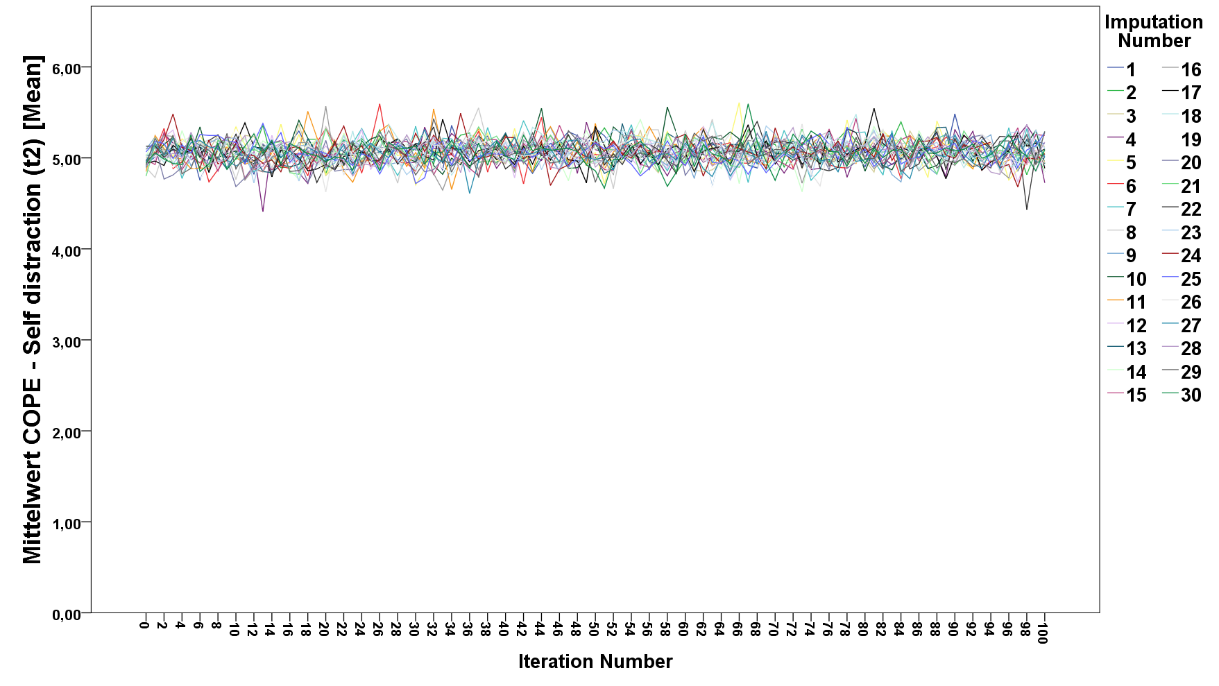


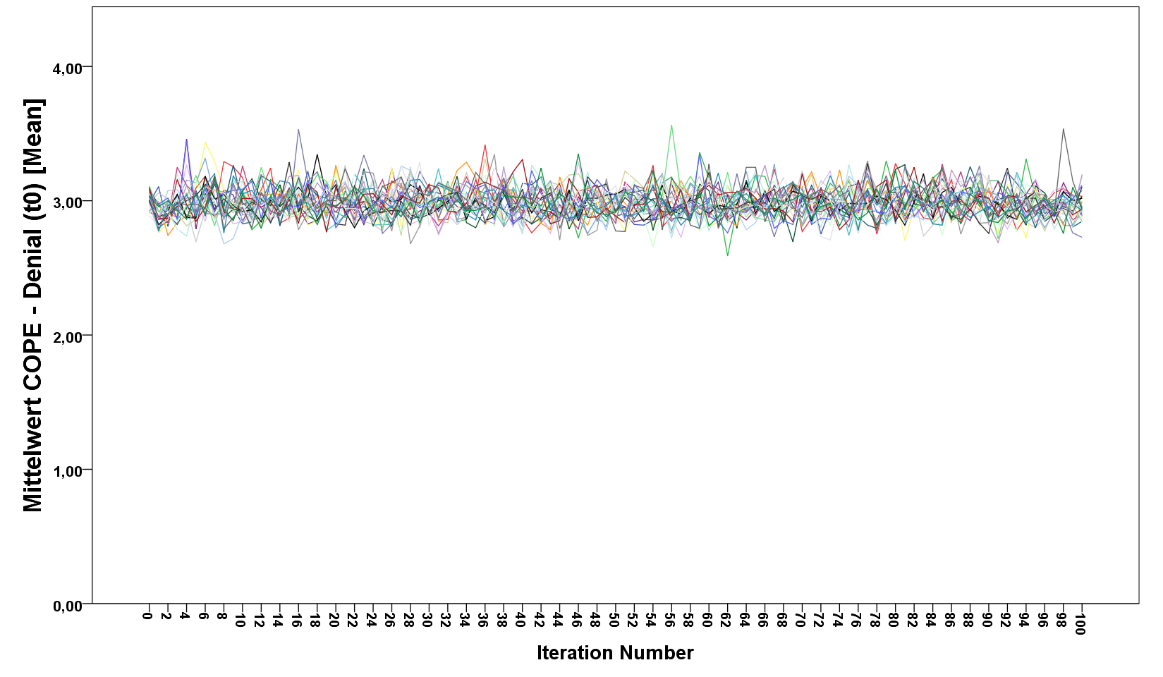

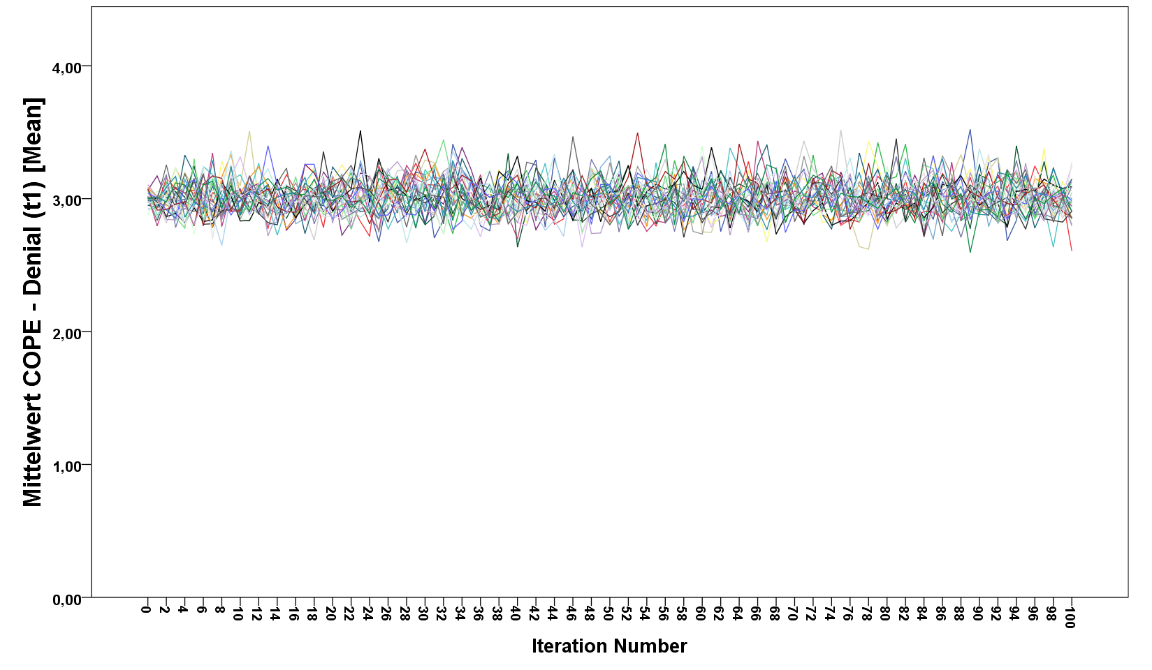

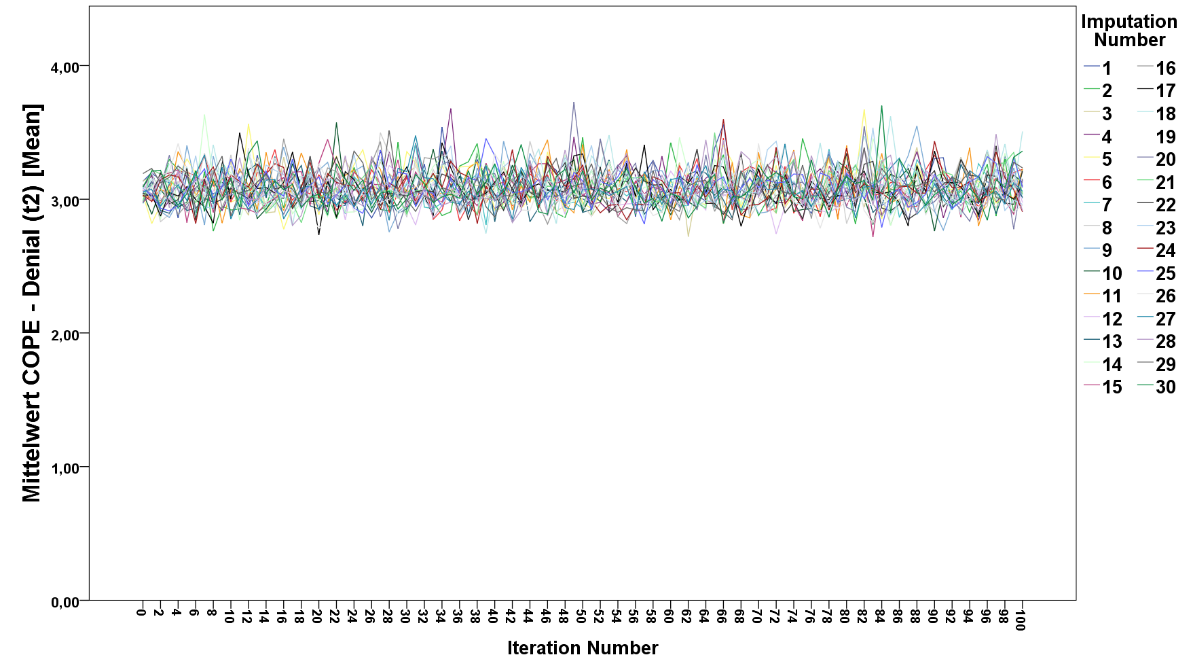


# **Supplementary Figure 3b.** Line chart depicting the imputed mean values of BSCL and Brief COPE questionnaires as a function of iteration number and imputation number


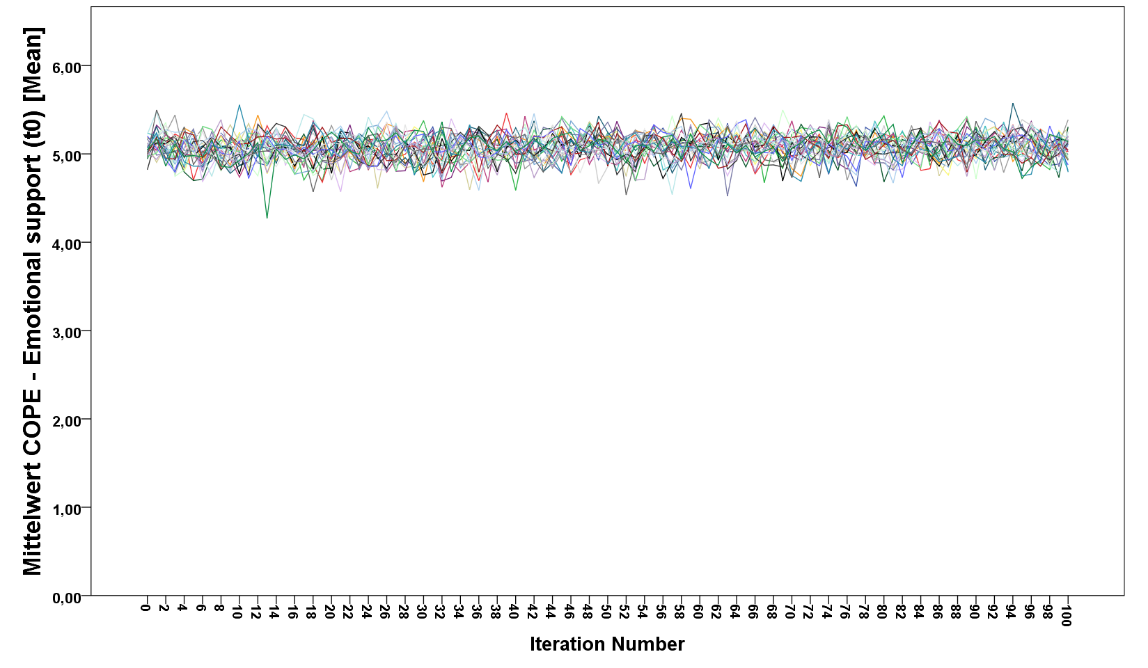

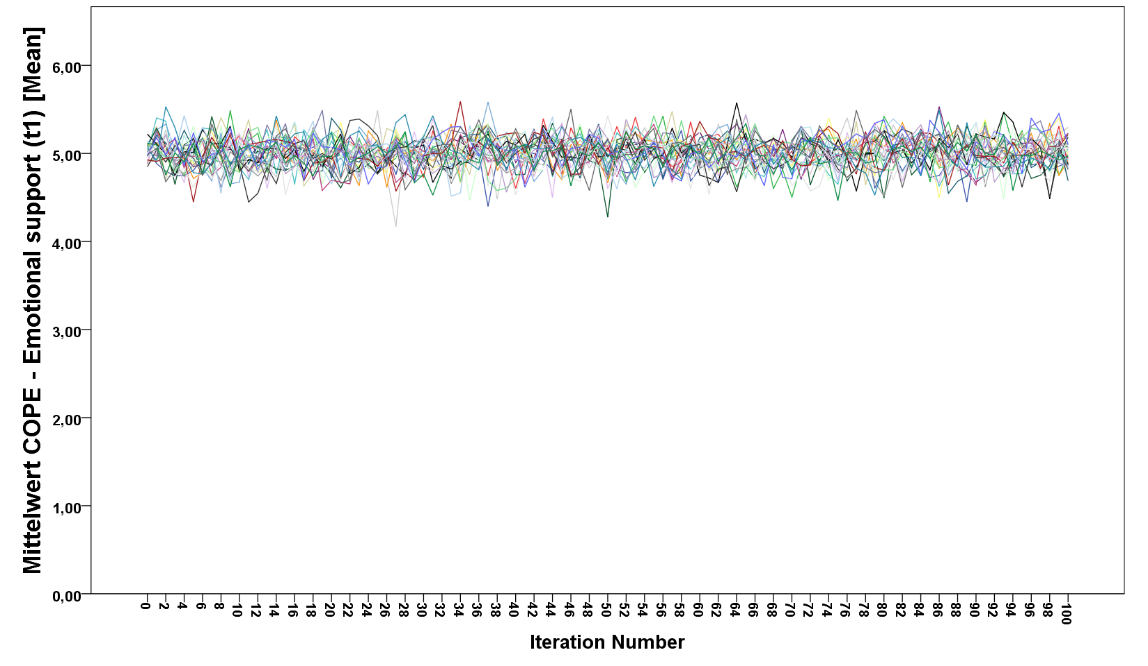

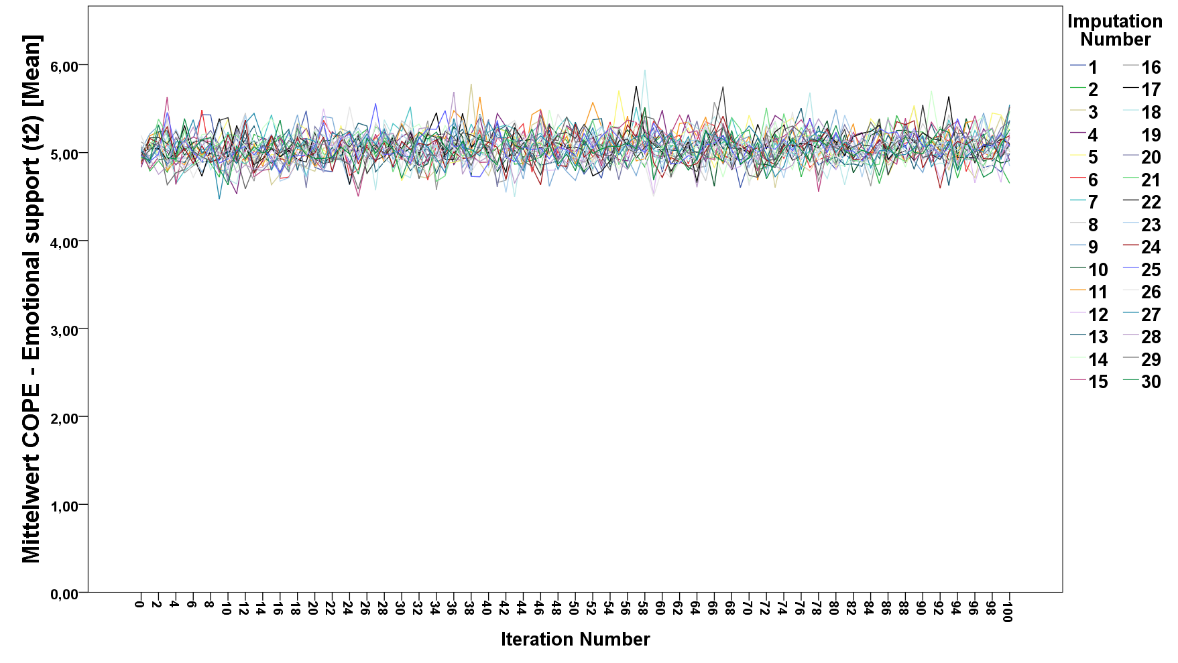


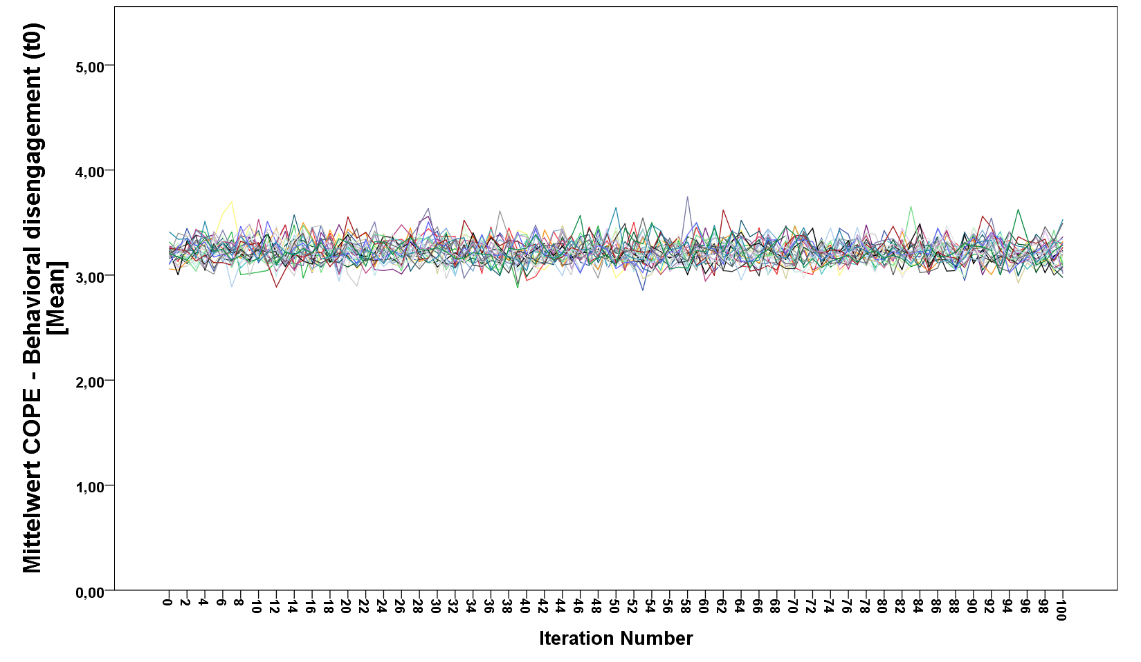

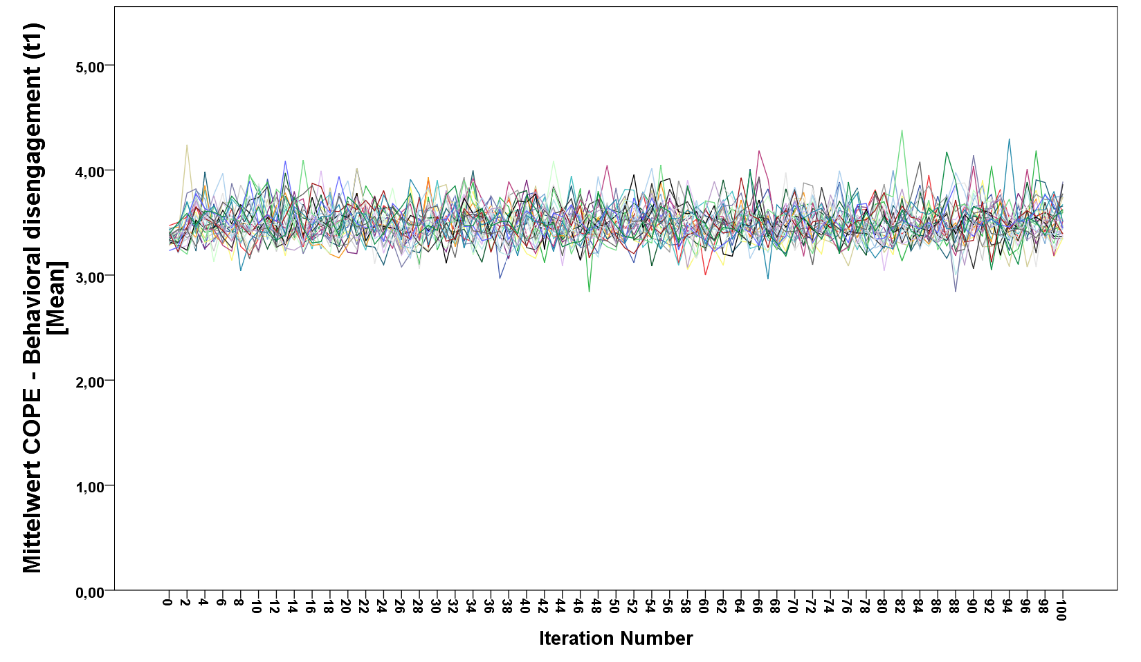

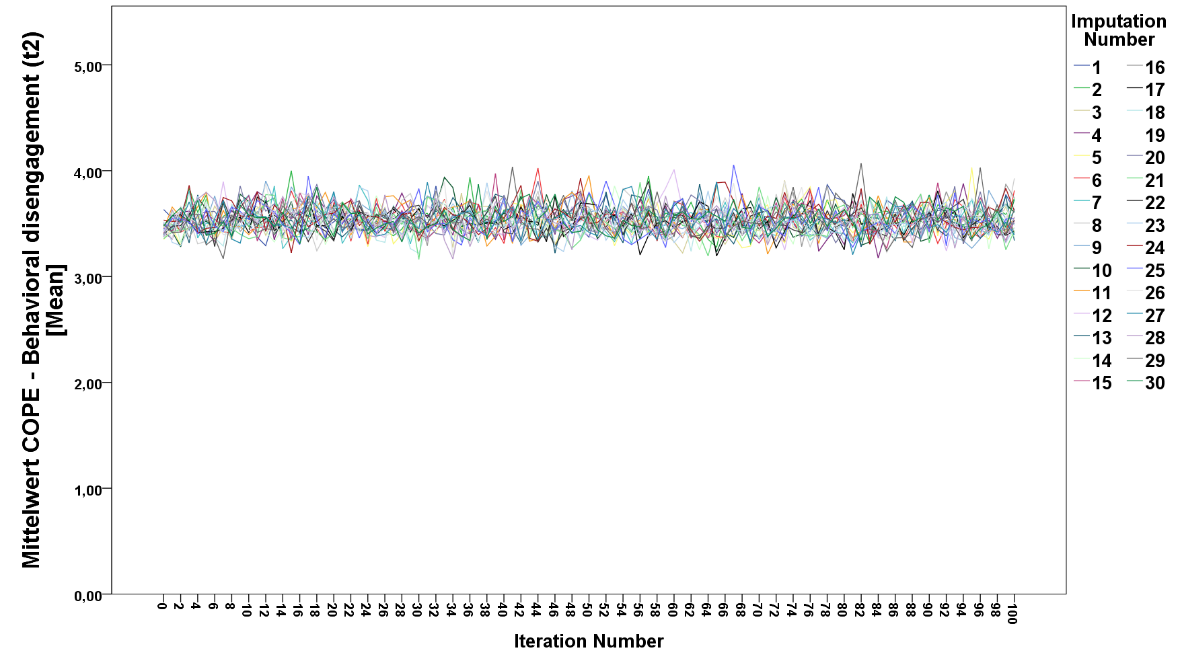


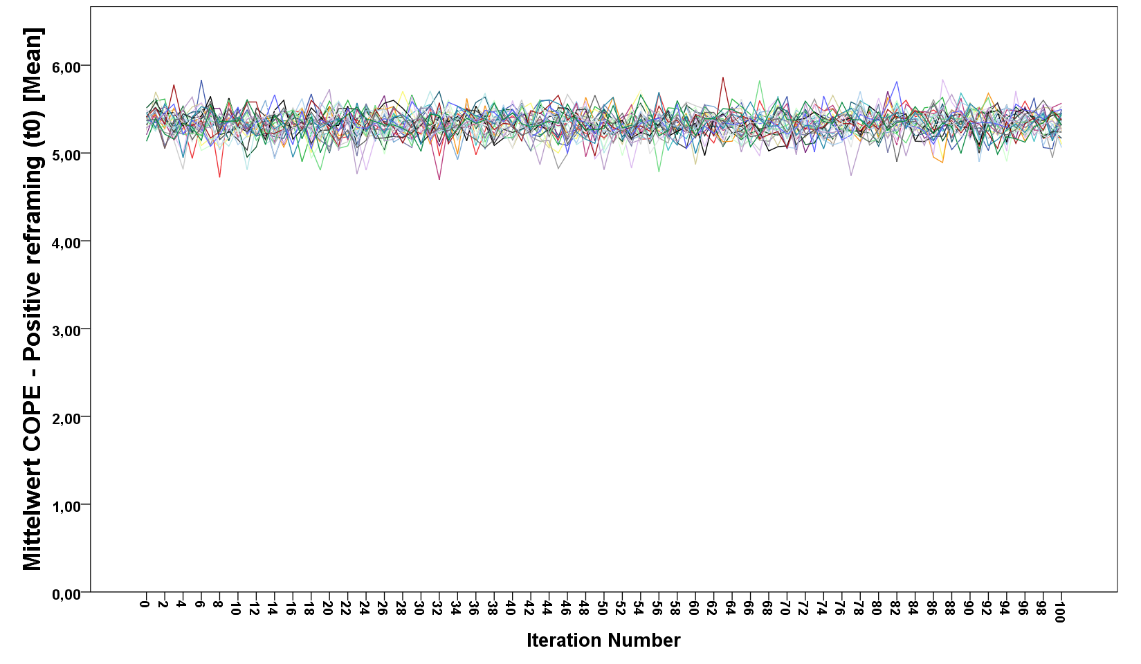

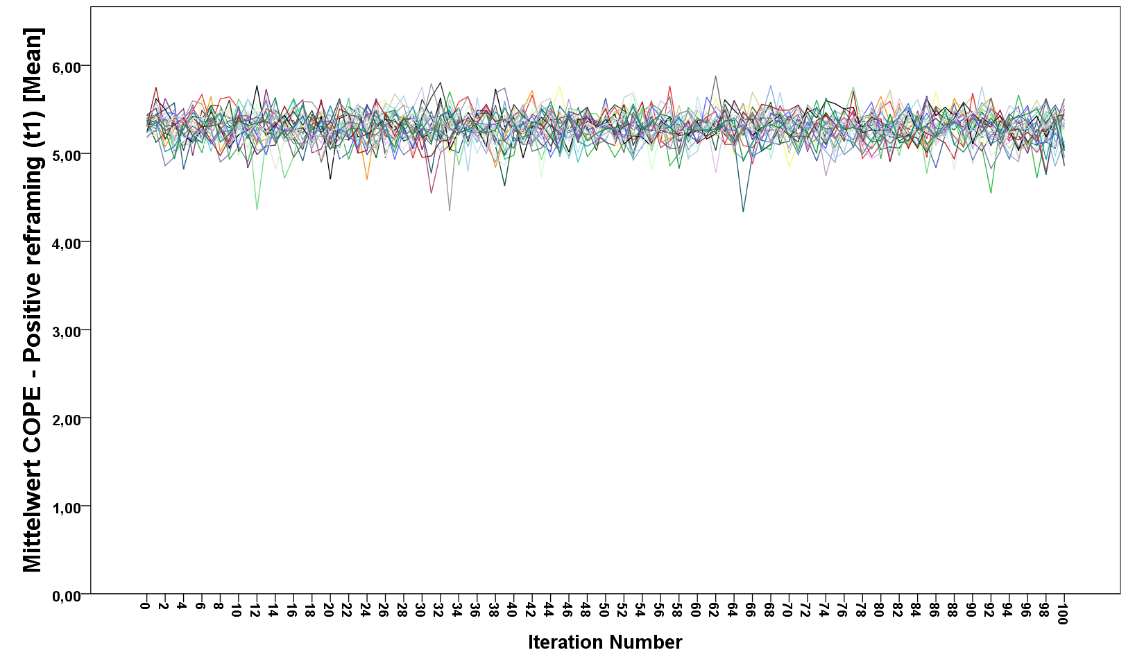

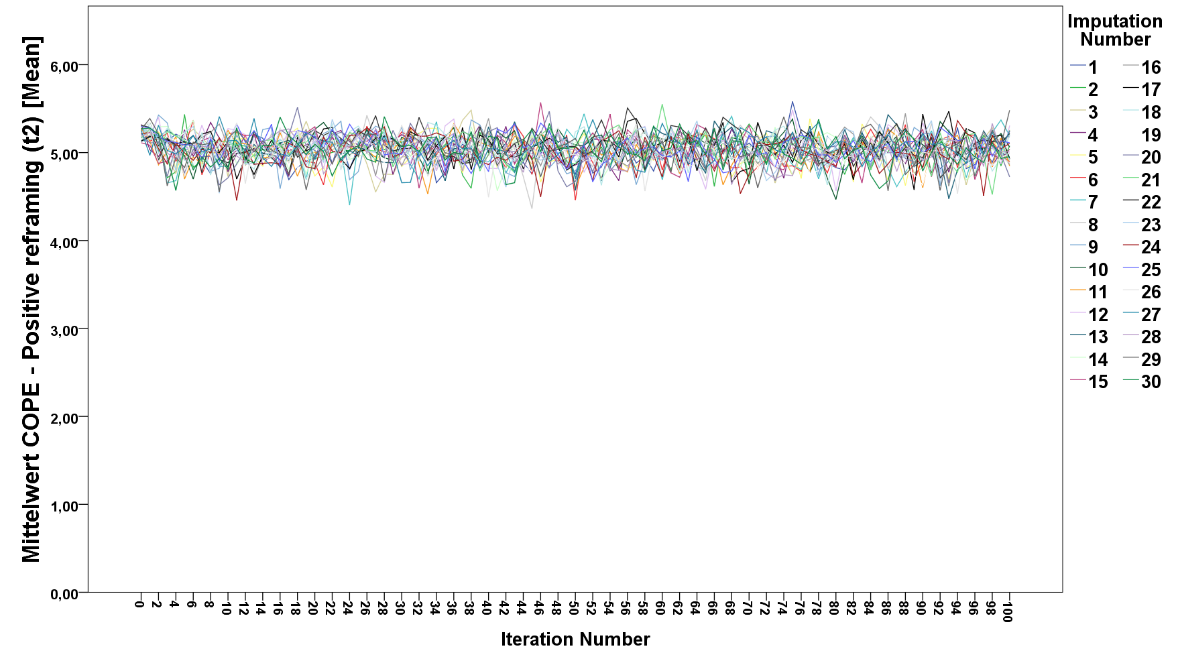


# **Supplementary Figure 3c.** Line chart depicting the imputed mean values of BSCL and Brief COPE questionnaires as a function of iteration number and imputation number


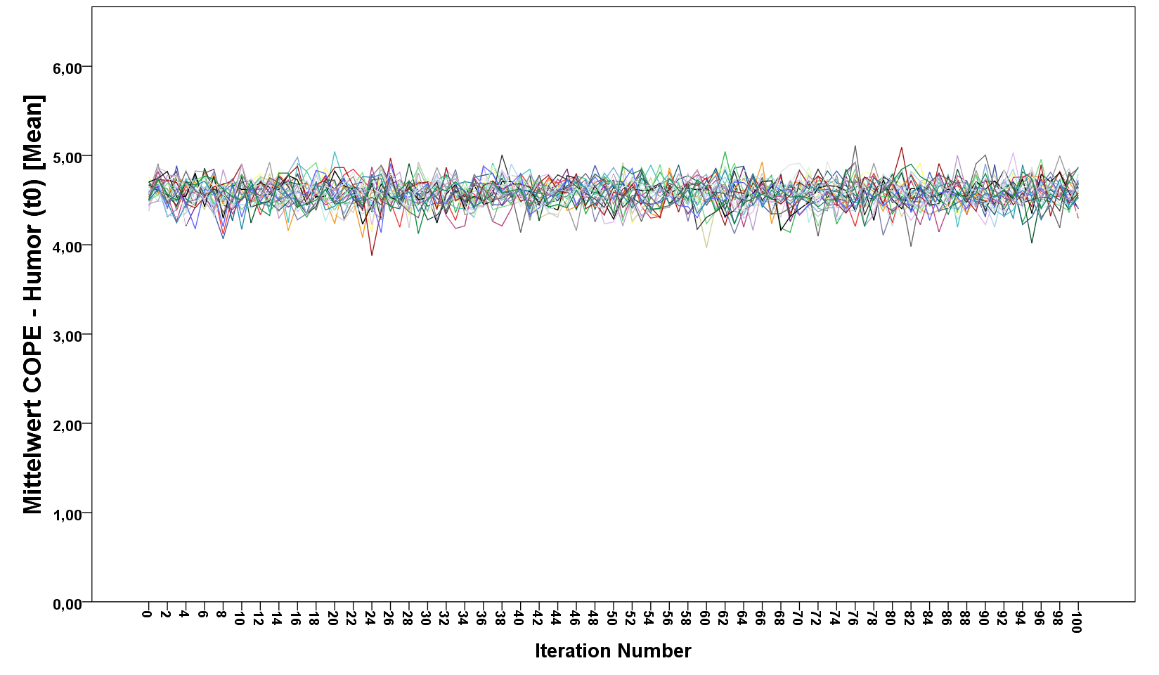

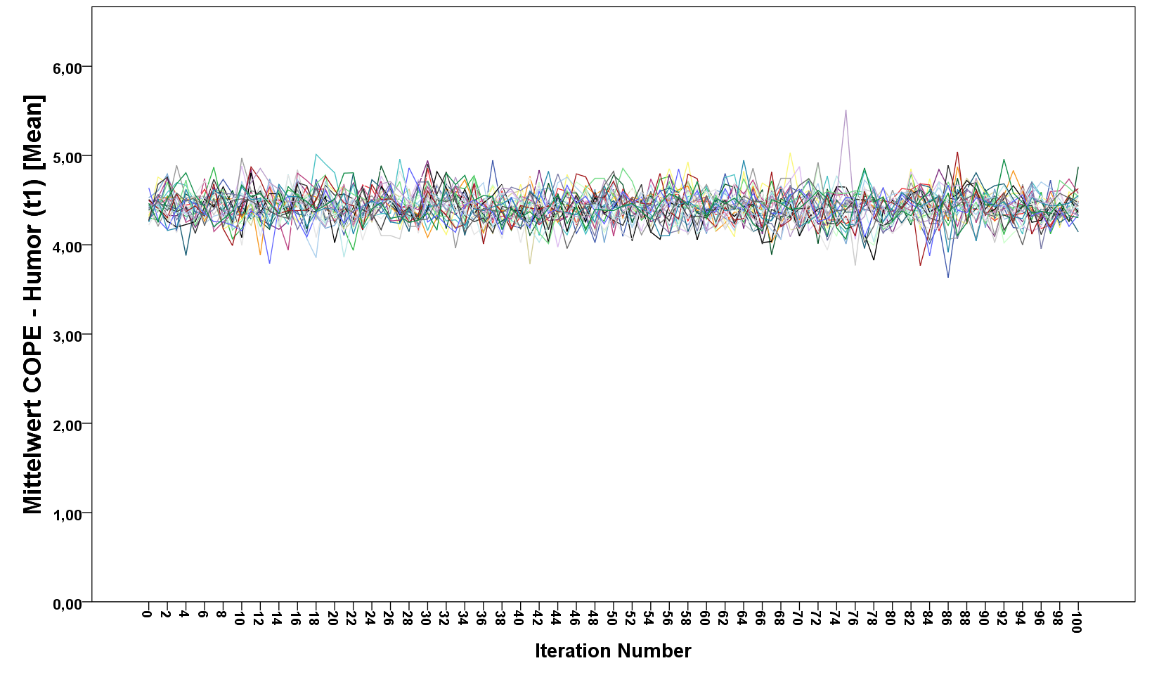

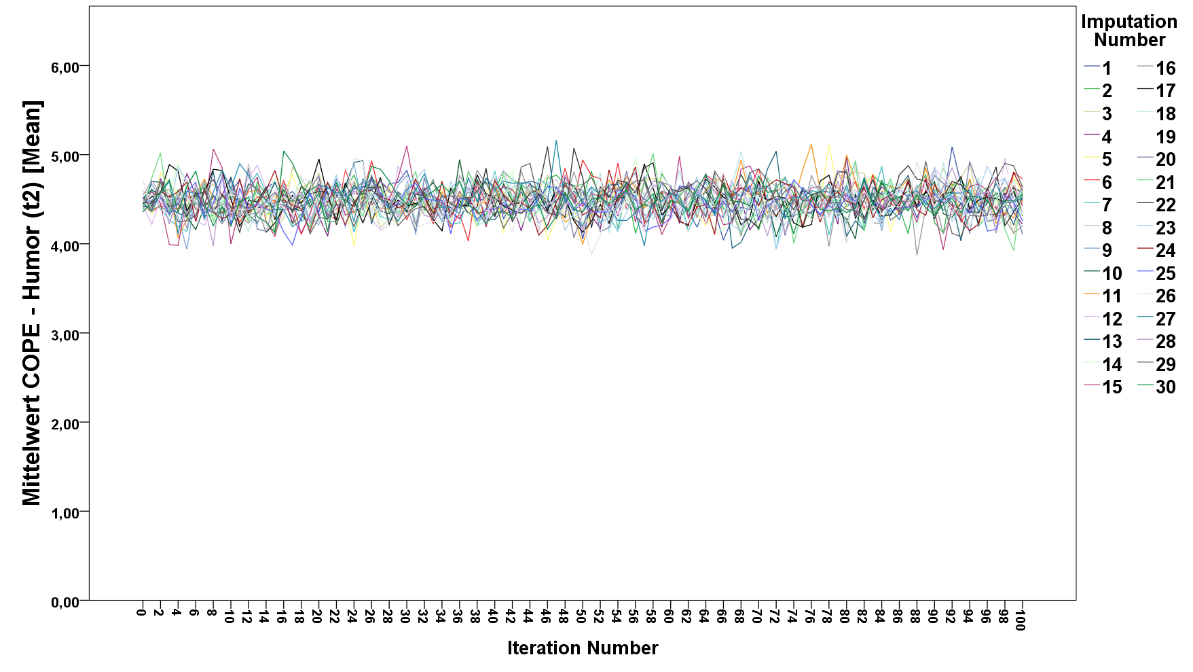

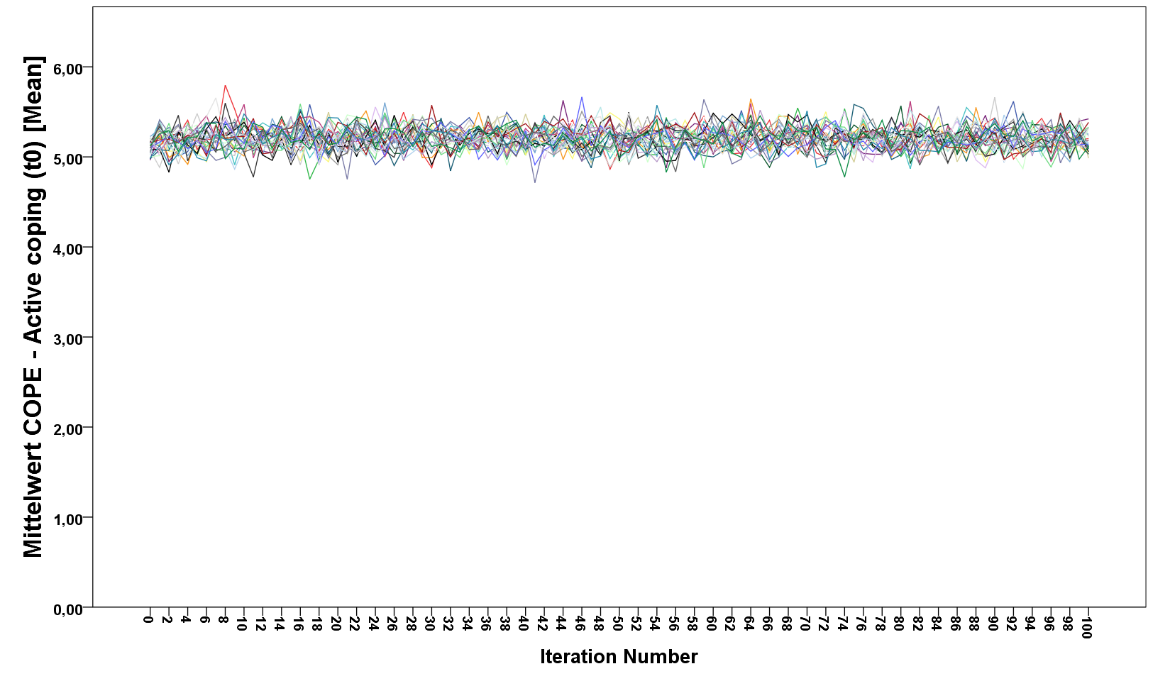

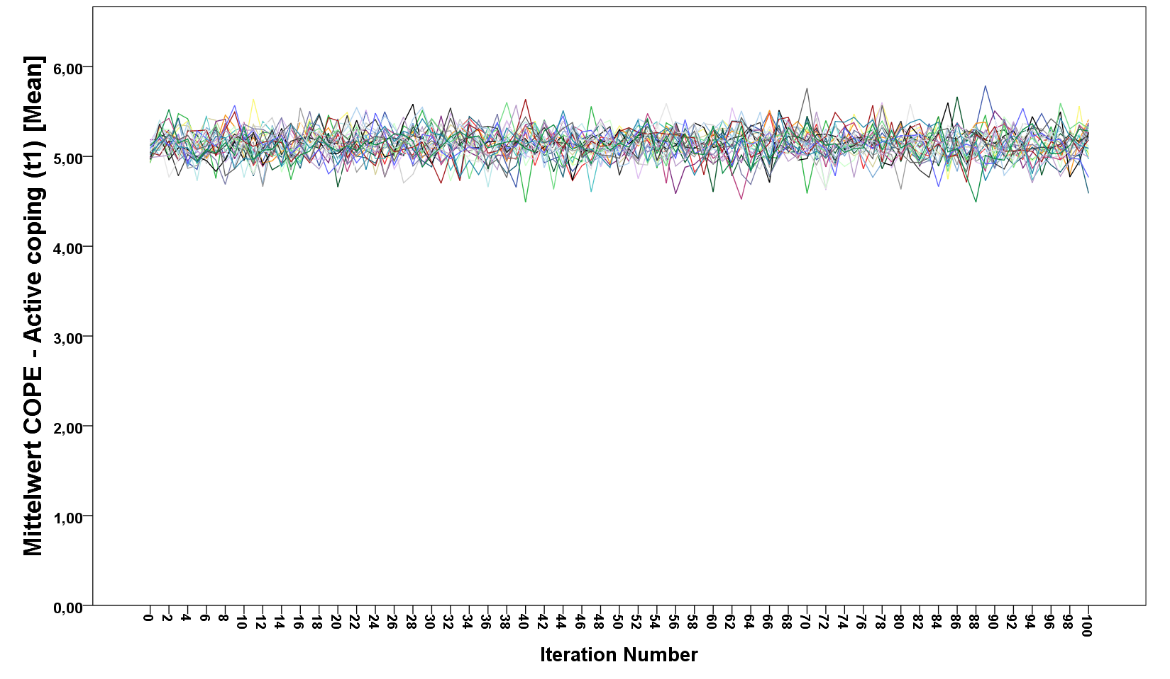

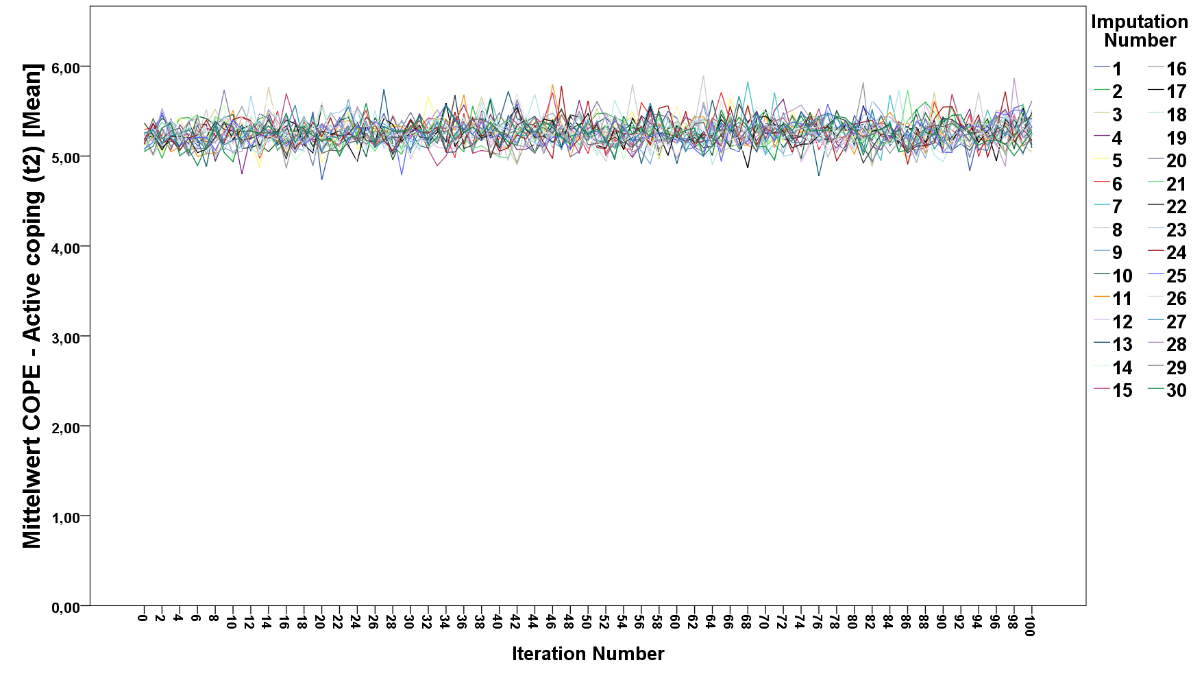


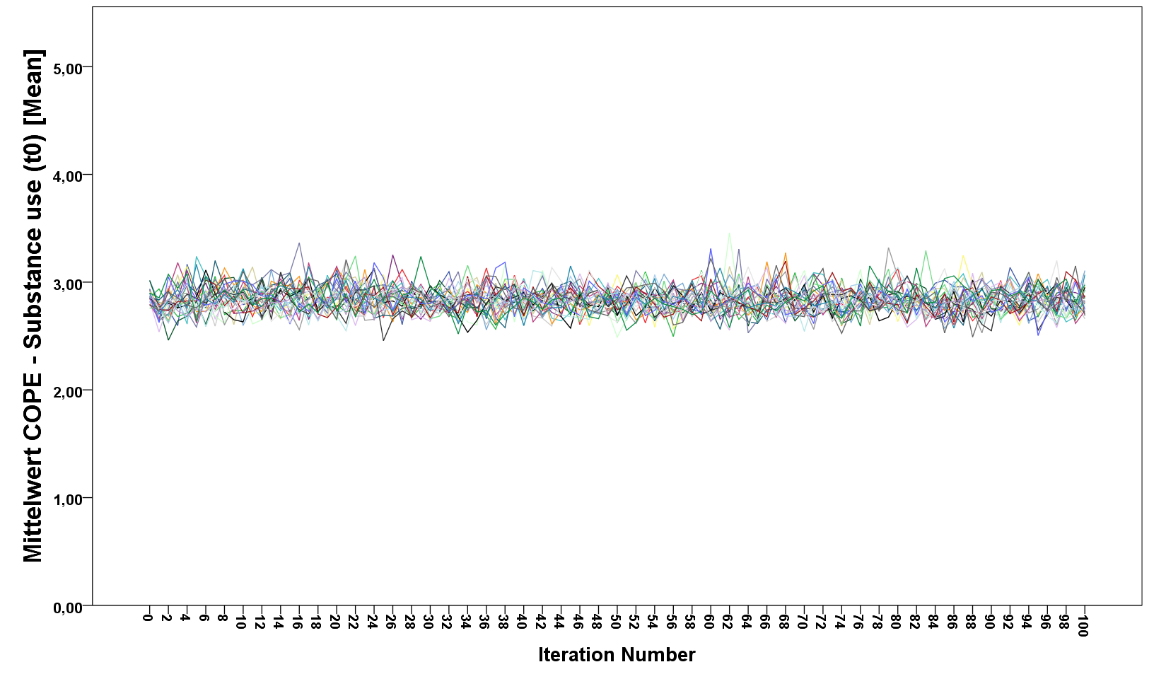

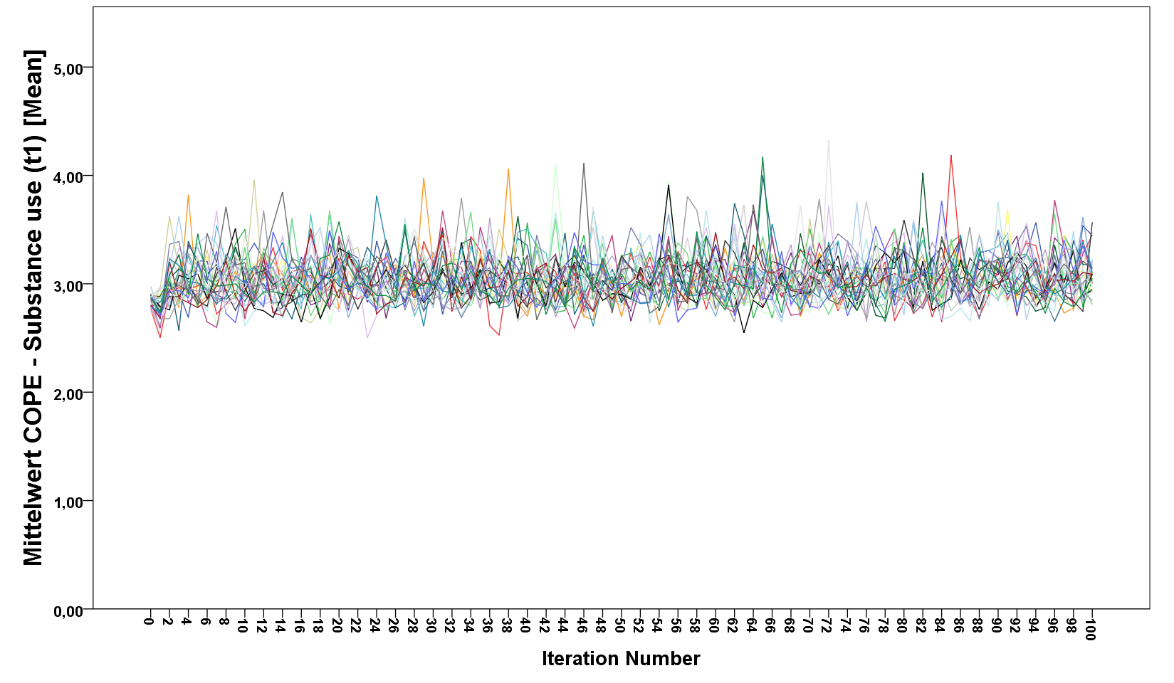

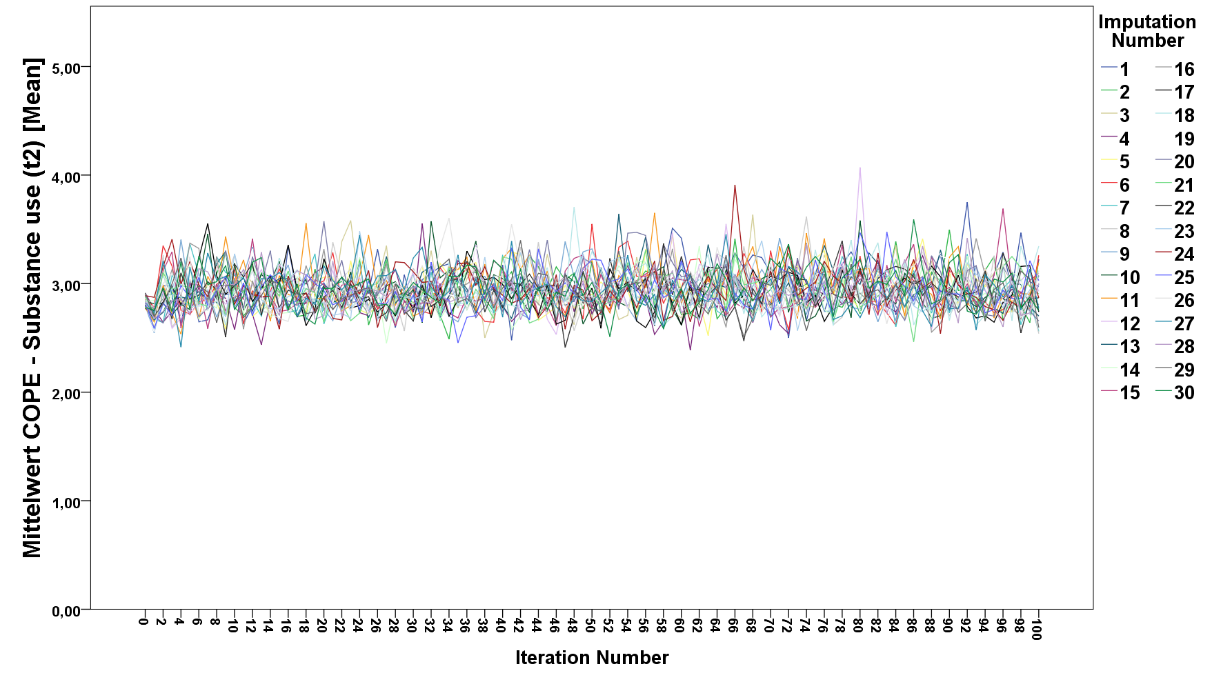


# **Supplementary Figure 3d.** Line chart depicting the imputed mean values of BSCL and Brief COPE questionnaires as a function of iteration number and imputation number


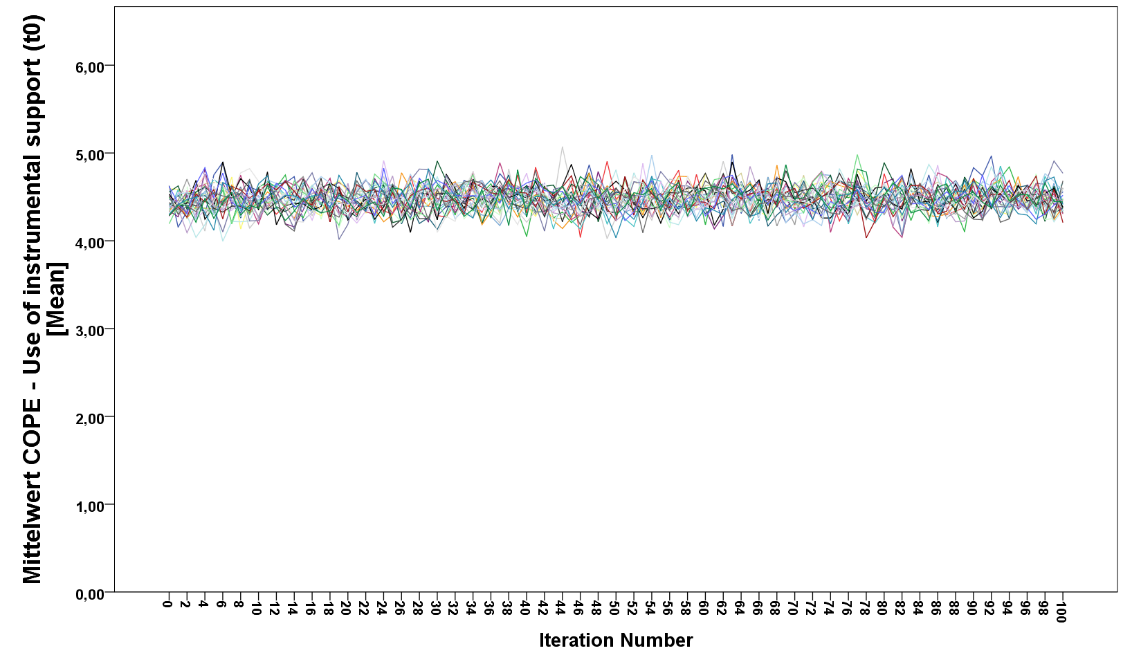

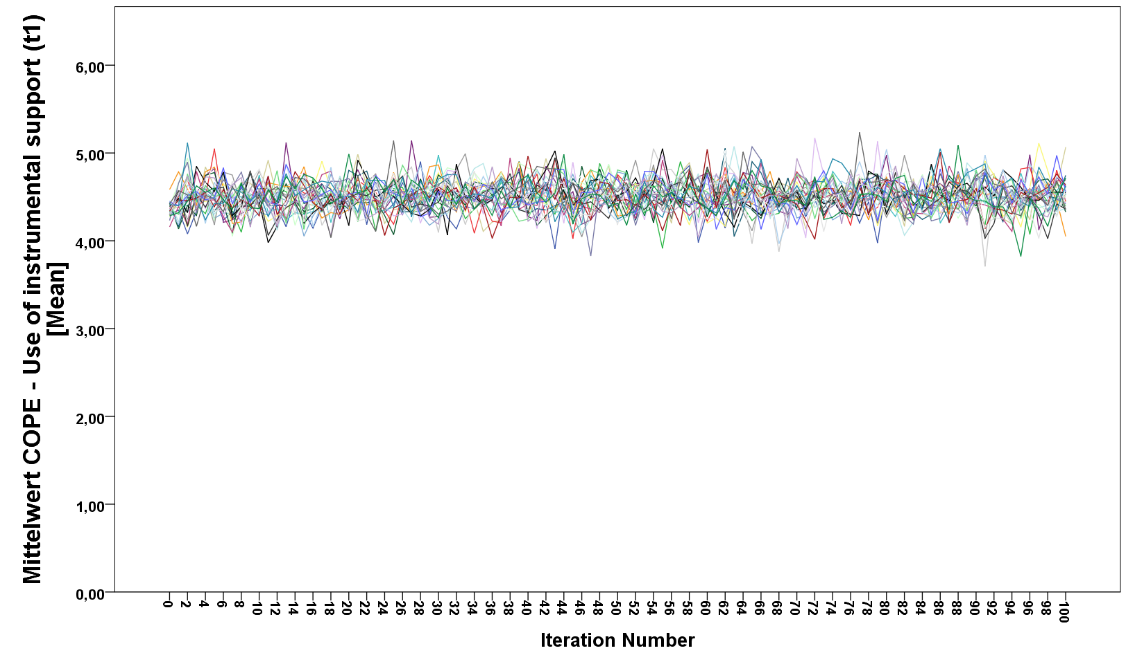

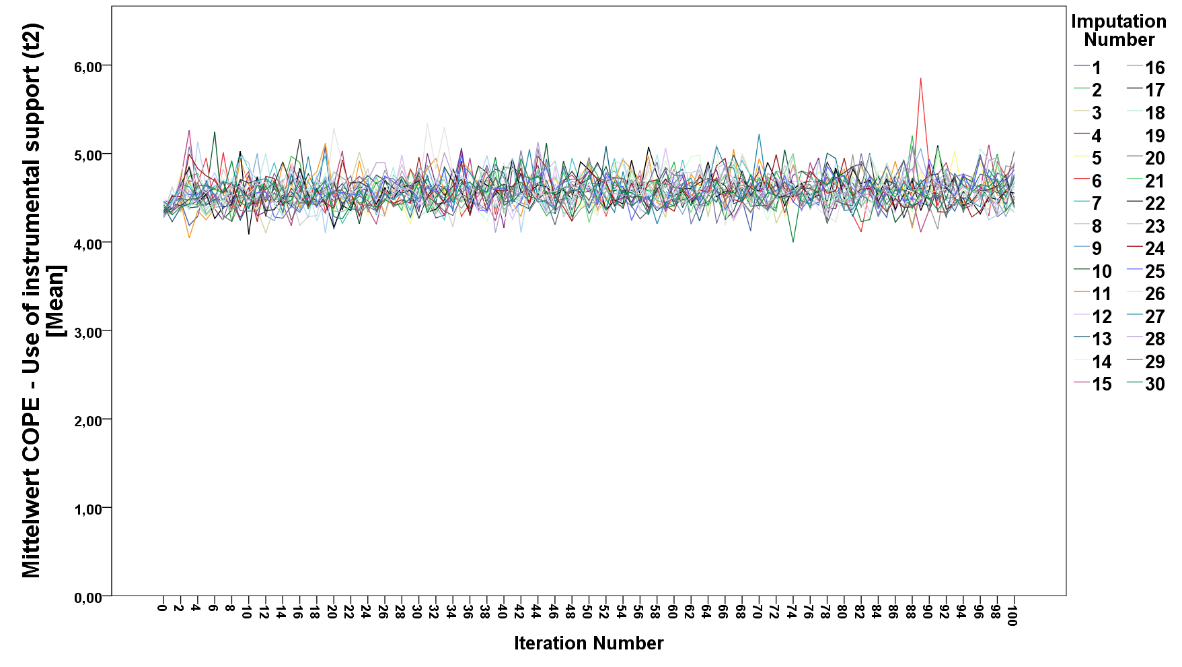


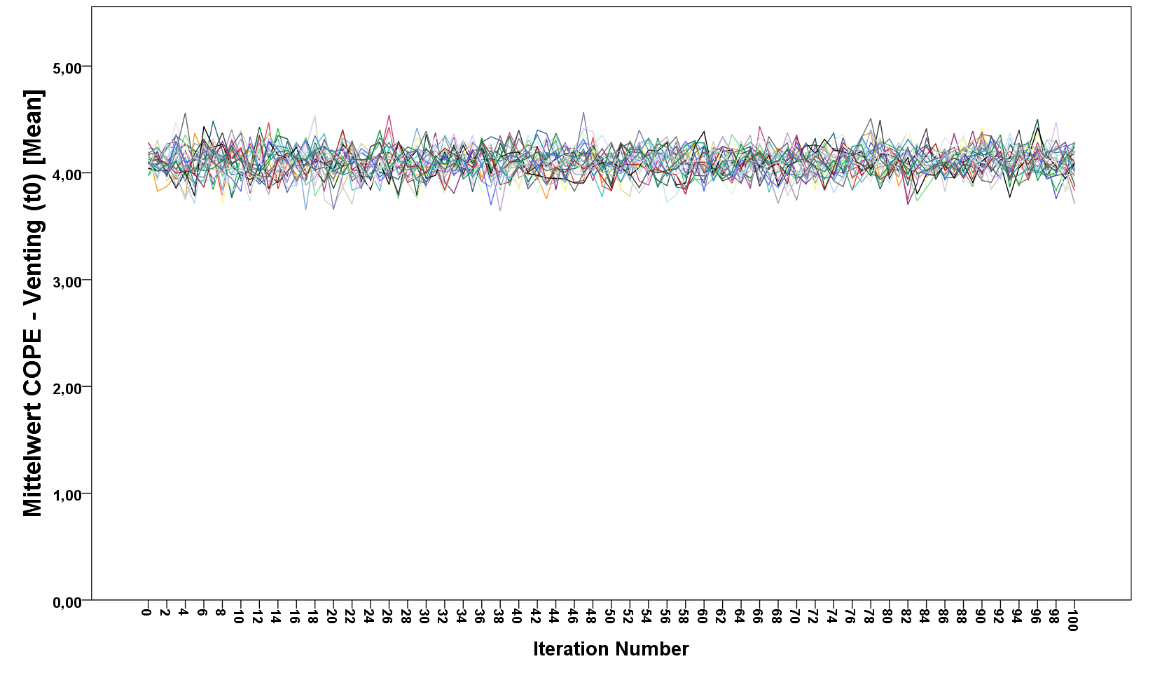

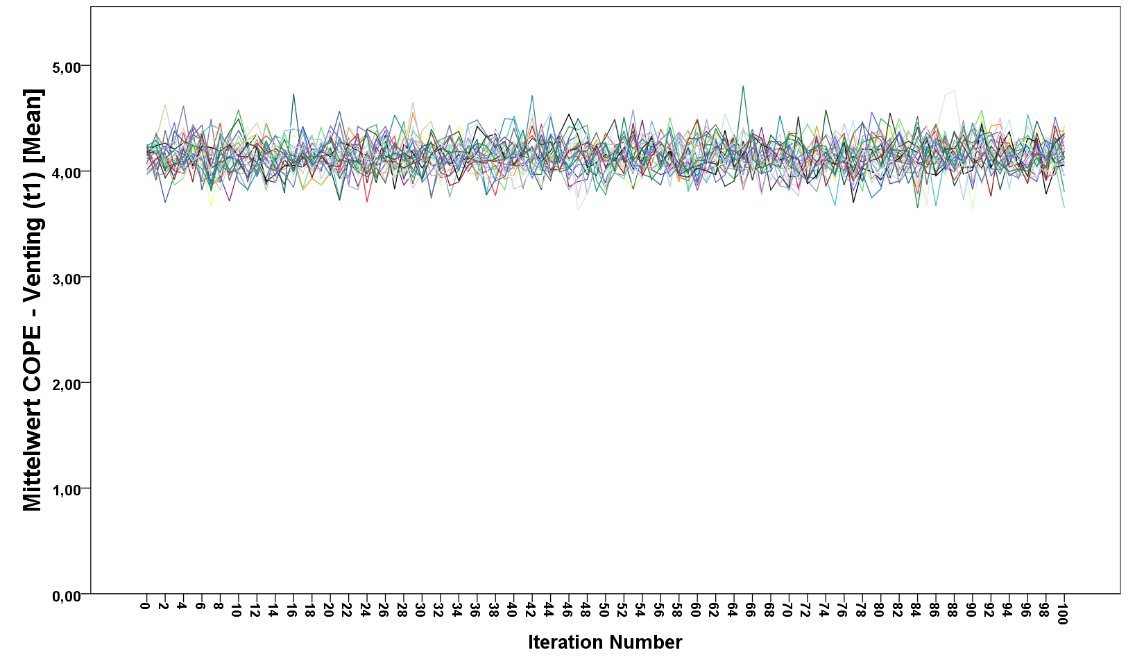

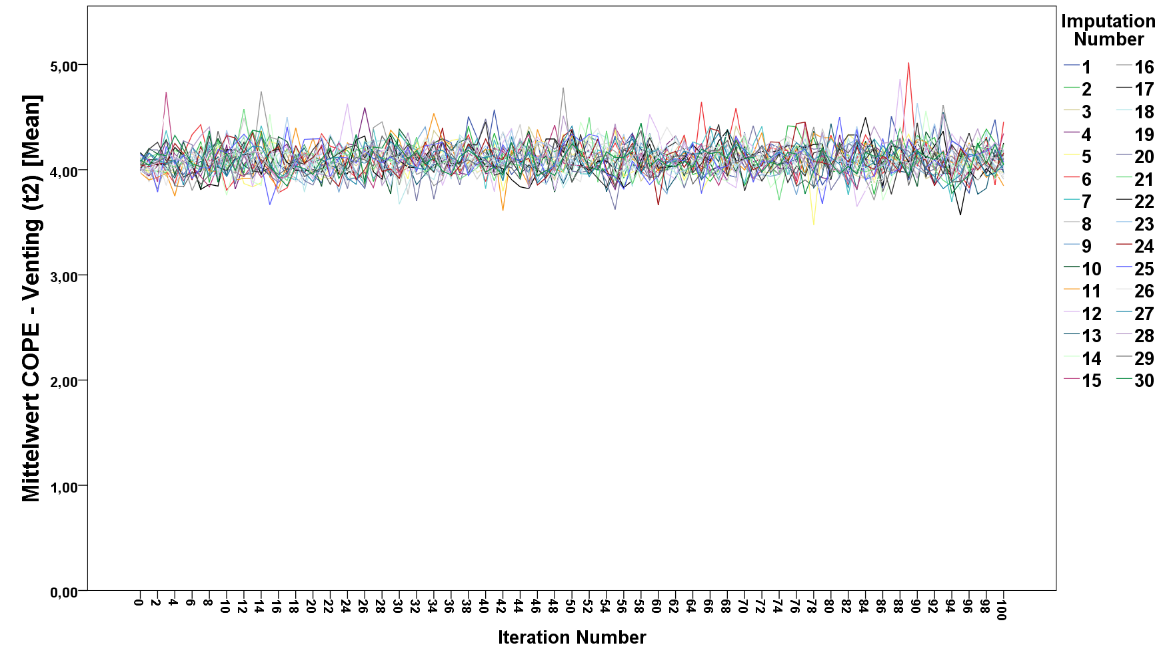


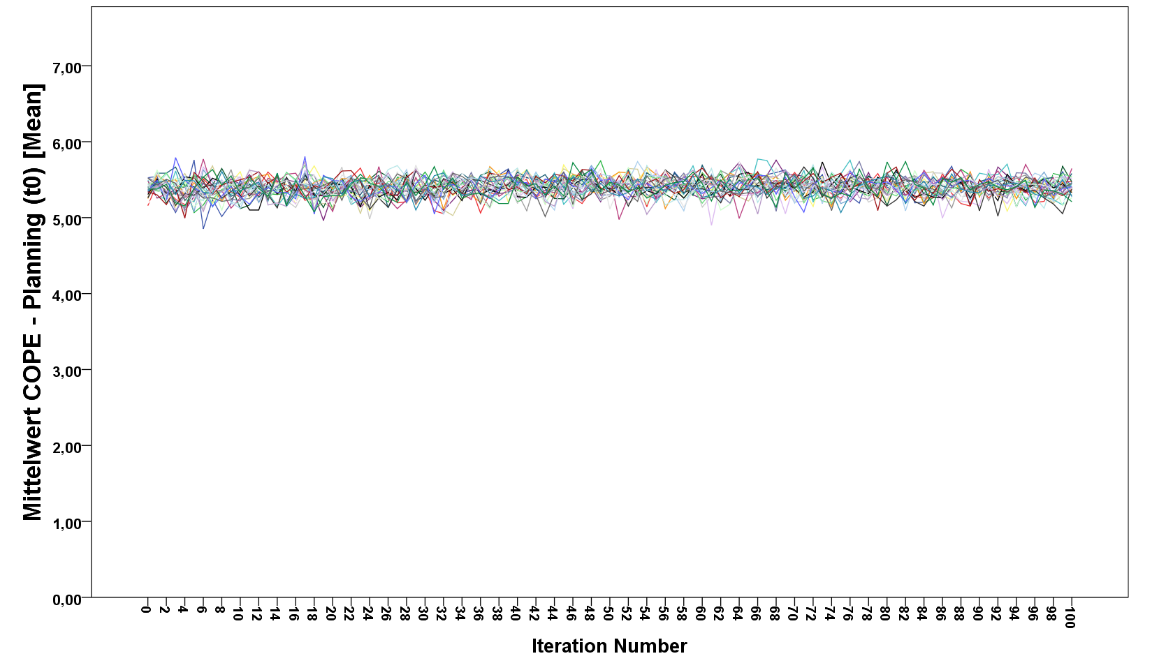

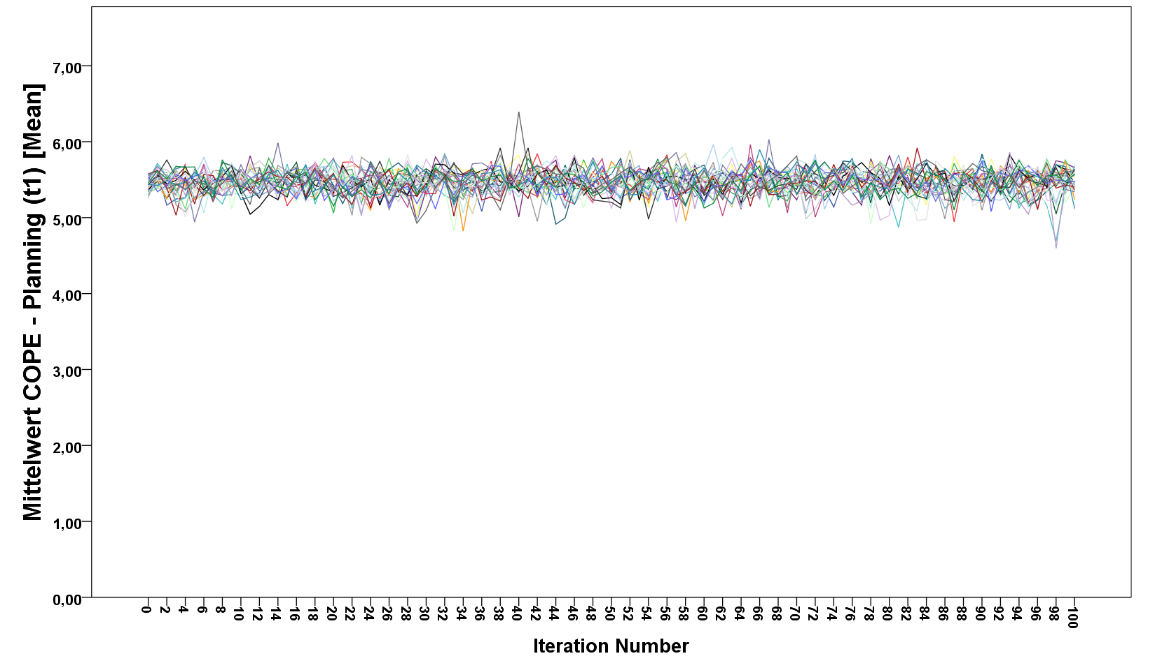

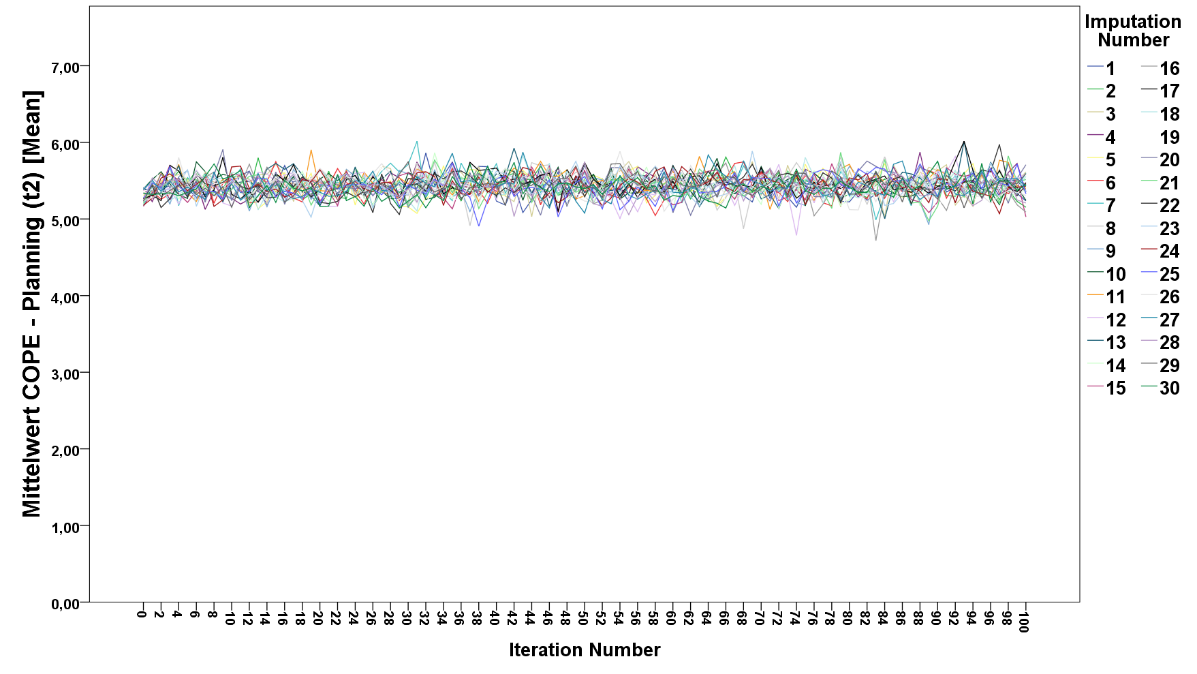


# **Supplementary Figure 3e.** Line chart depicting the imputed mean values of BSCL and Brief COPE questionnaires as a function of iteration number and imputation number


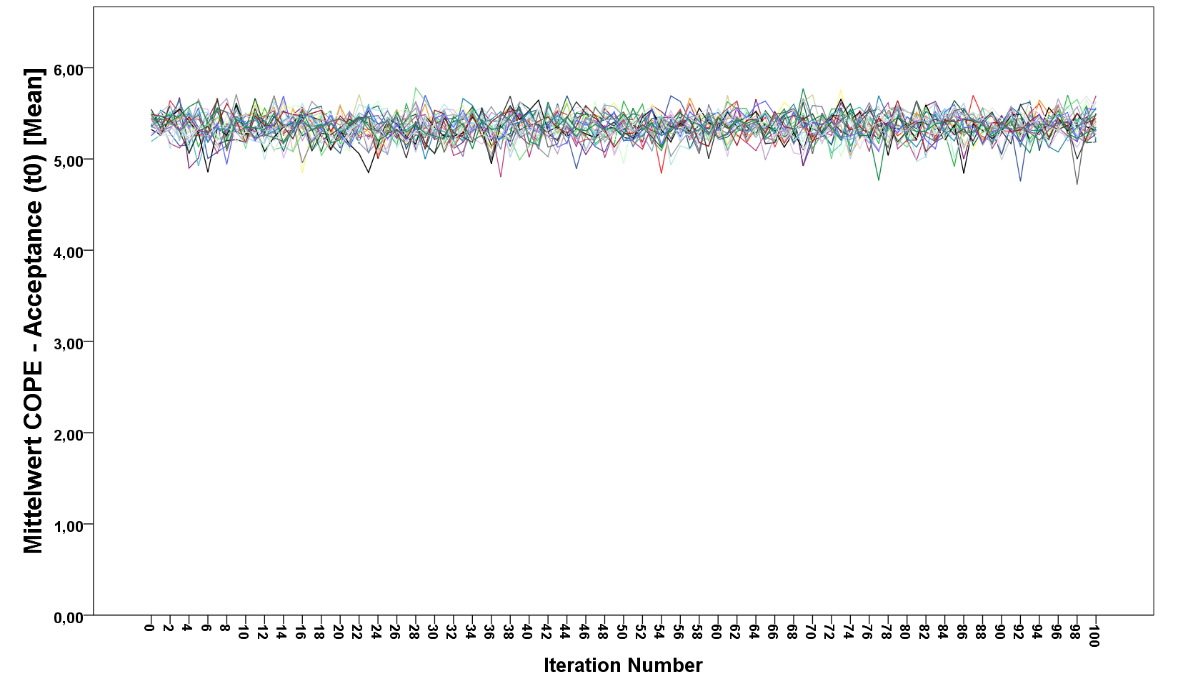

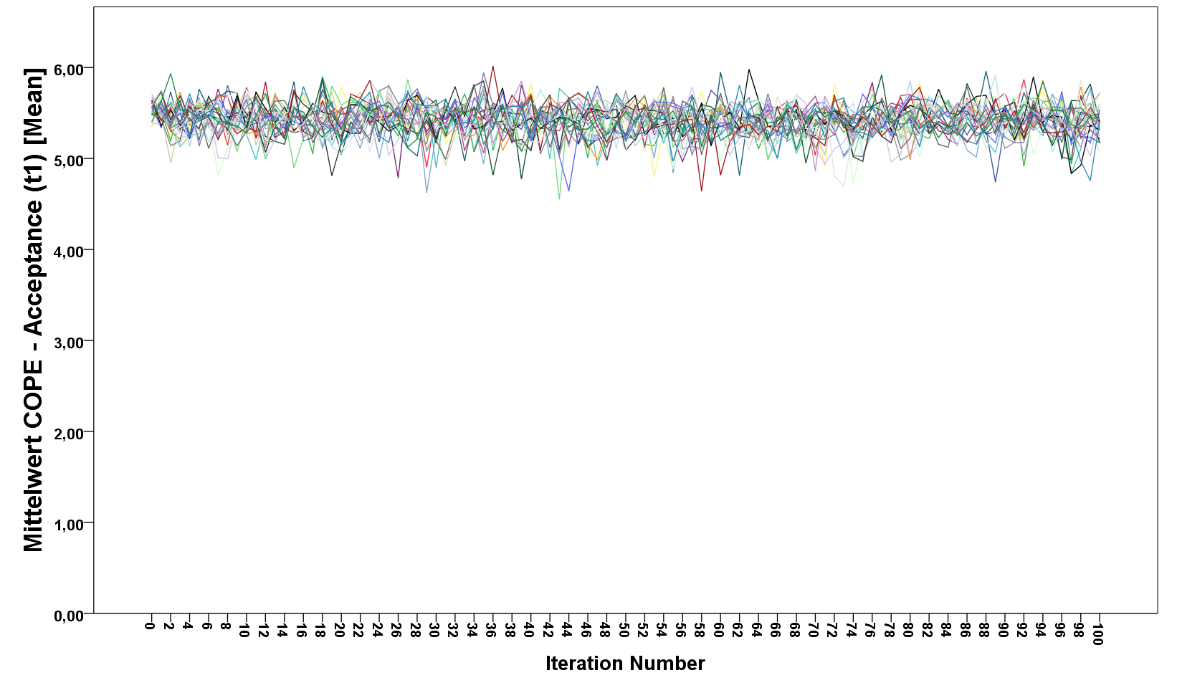

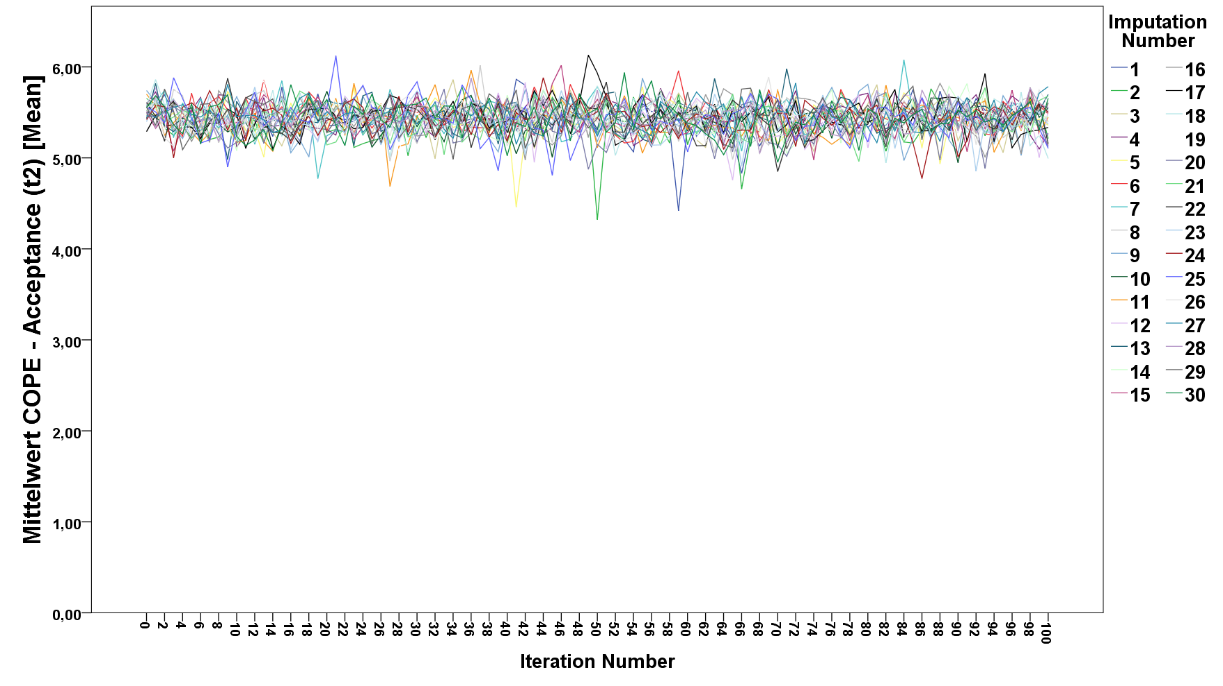


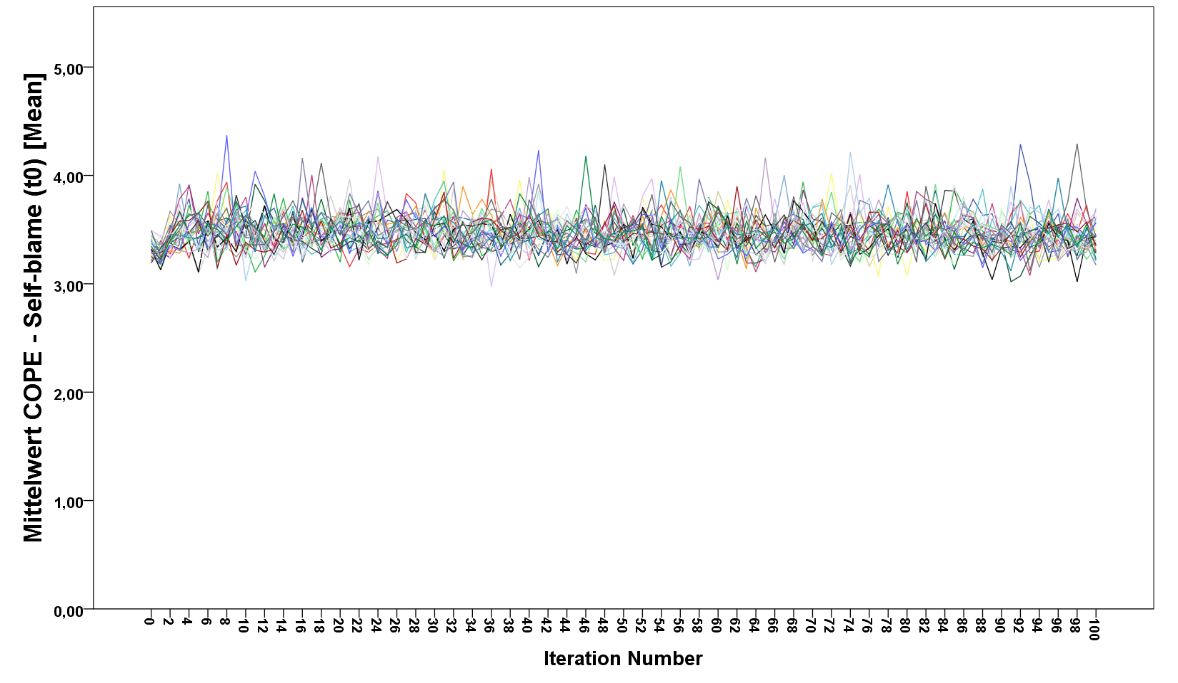

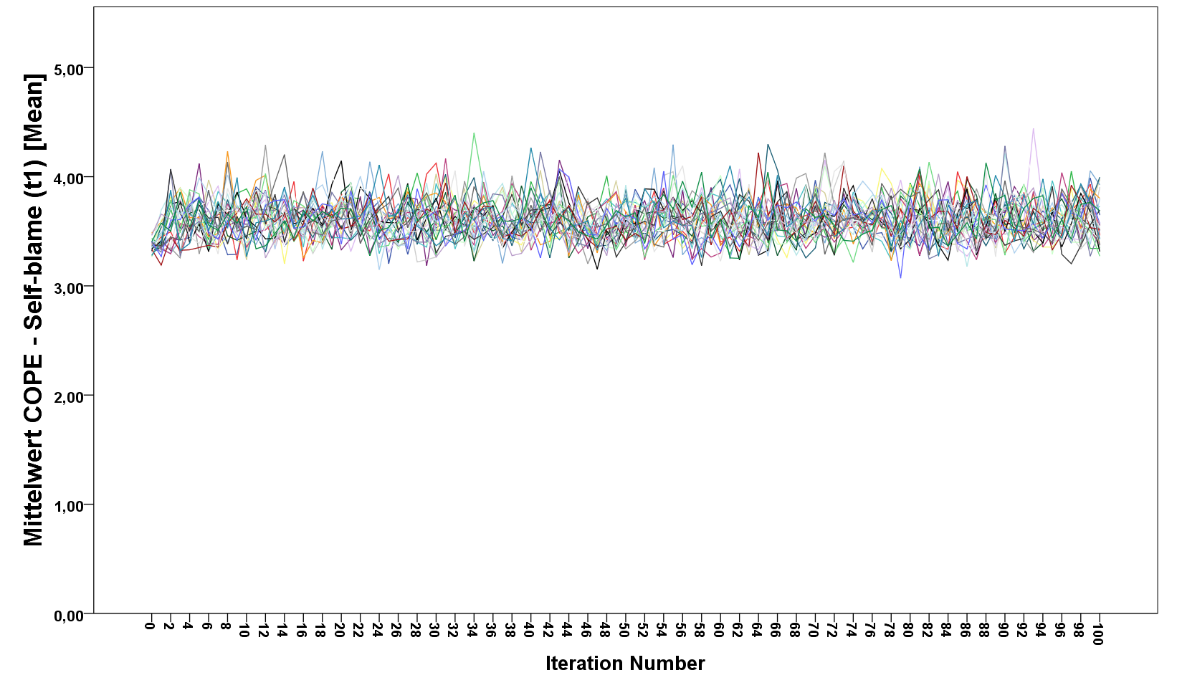

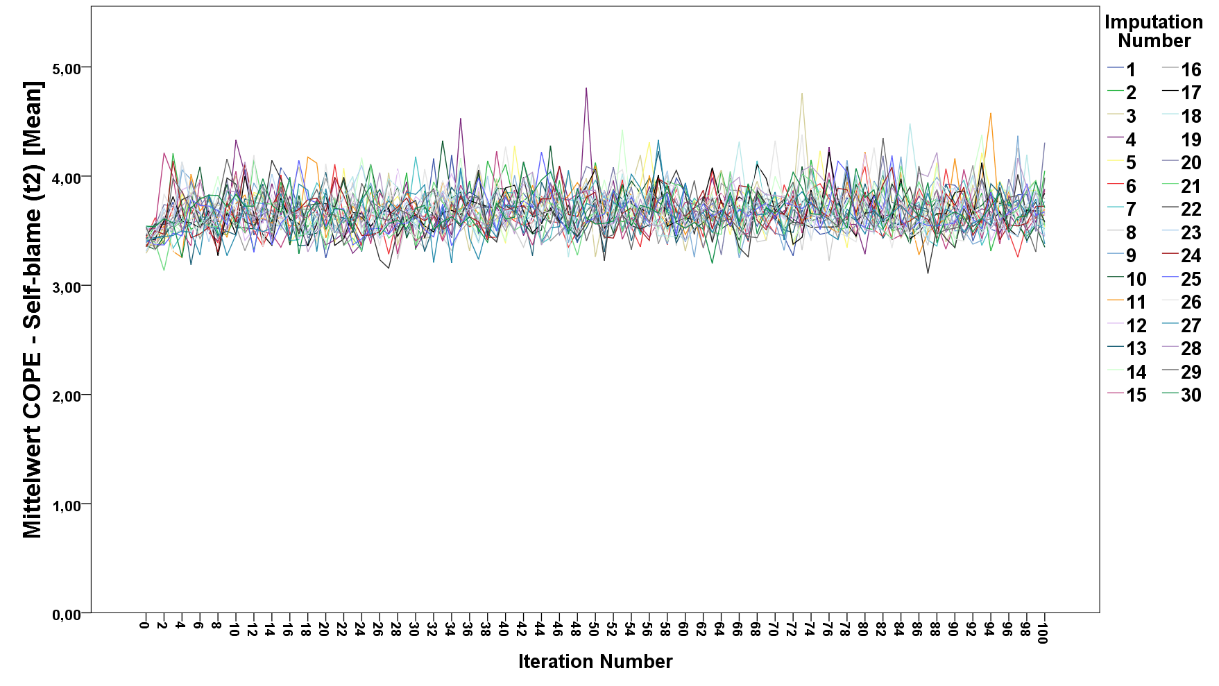


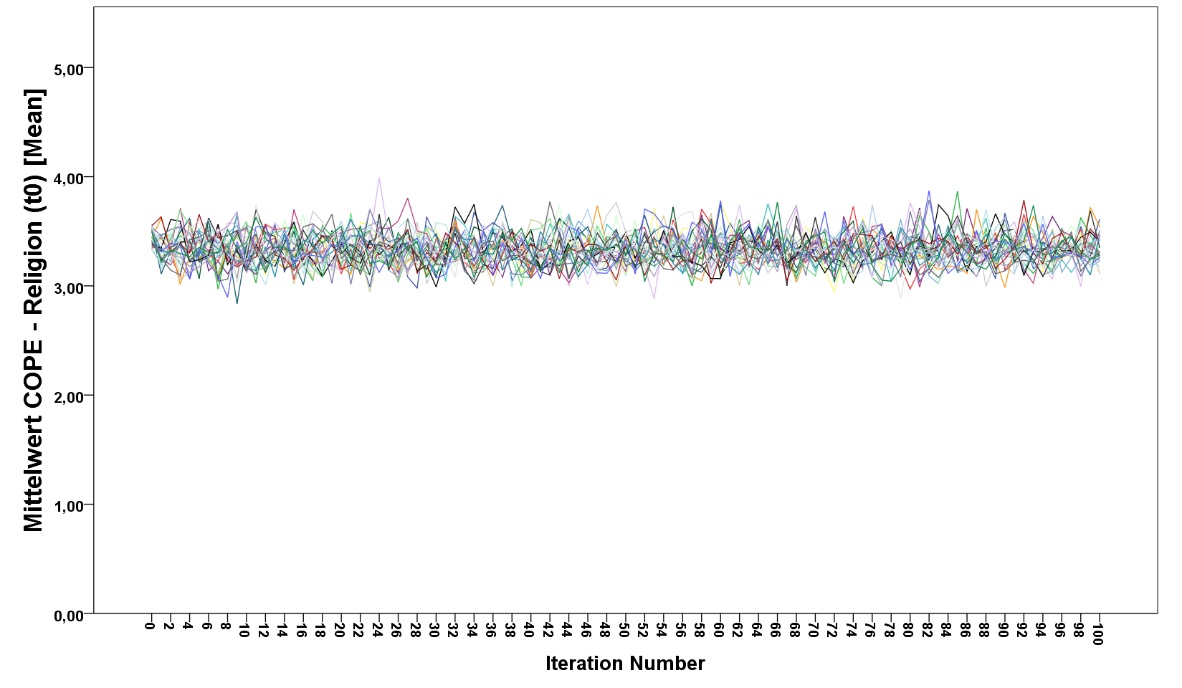

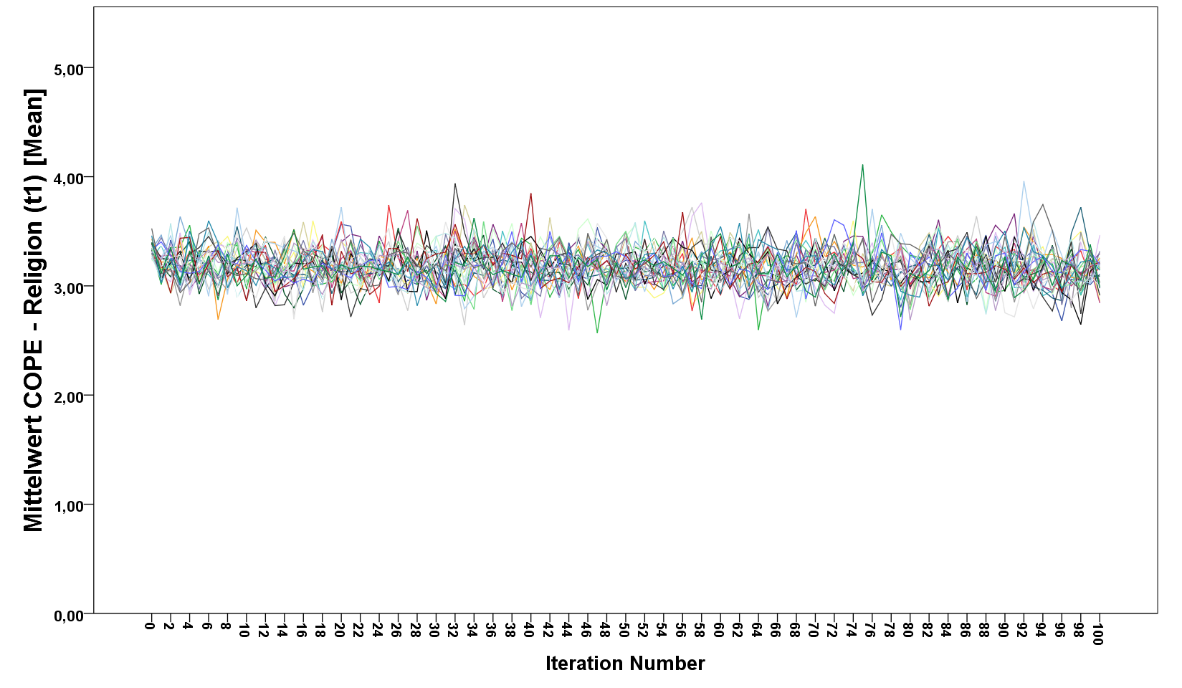

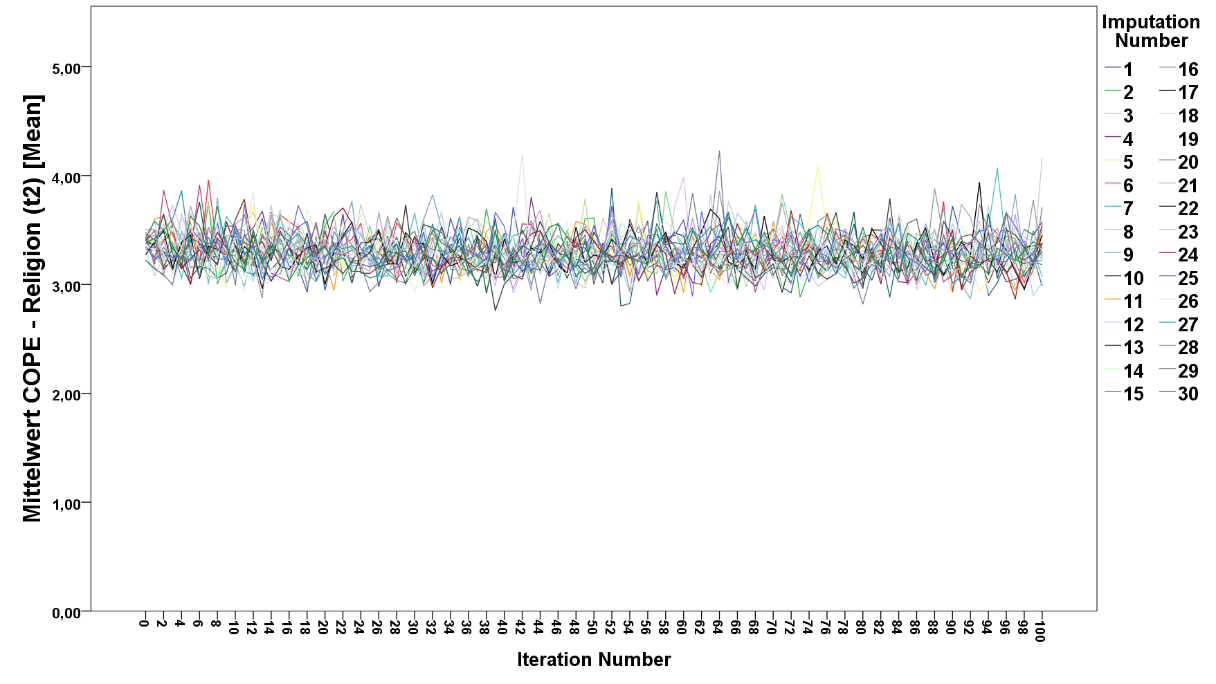


# **Supplementary Table 6.** Pooled estimates of fixed effects in the univariate Linear Mixed Model after multiple imputation procedure

| **Model** | **Coping response** | **Estimate** | **S.E.** | **t** | **95% CI (Estimate)** | | **p-value** |
| --- | --- | --- | --- | --- | --- | --- | --- |
|  |  |  |  |  | **LB** | **UB** |  |
| M1.1 Pooled | Acceptance | -5.022 | 0.667 | -7.529 | -6.329 | -3.715 | <0.0001 |
| M2.1 Pooled | Acceptance (t0) | -4.598 | 1.014 | -4.535 | -6.585 | -2.611 | <0.0001 |
|  | Acceptance (t1 vs. t0) | -0.436 | 1.199 | -0.364 | -2.786 | 1.914 | 0.4441 |
|  | Acceptance (t2 vs. t0) | -0.574 | 1.135 | -0.506 | -2.799 | 1.651 | 0.5890 |
| M1.2 Pooled | Active coping | -3.543 | 0.678 | -5.226 | -4.872 | -2.214 | <0.0001 |
| M2.2 Pooled | Active coping (t0) | -3.226 | 1.08 | -2.987 | -5.343 | -1.109 | 0.0044 |
|  | Active coping (t1 vs. t0) | 0.446 | 1.246 | 0.358 | -1.996 | 2.888 | 0.5773 |
|  | Active coping (t2 vs. t0) | -1.192 | 1.378 | -0.865 | -3.893 | 1.509 | 0.0781 |
| M1.3 Pooled | Behavioural disengagement | 9.314 | 0.776 | 12.003 | 7.793 | 10.835 | <0.0001 |
| M2.3 Pooled | Behavioural disengagement (t0) | 12.397 | 1.321 | 9.385 | 9.808 | 14.986 | <0.0001 |
|  | Behavioural disengagement (t1 vs. t0) | -2.384 | 1.54 | -1.548 | -5.402 | 0.634 | 0.2954 |
|  | Behavioural disengagement (t2 vs. t0) | -4.297 | 1.576 | -2.727 | -7.386 | -1.208 | 0.0161 |
| M1.4 Pooled | Denial | 12.519 | 0.822 | 15.230 | 10.908 | 14.130 | <0.0001 |
| M2.4 Pooled | Denial (t0) | 13.679 | 1.185 | 11.543 | 11.356 | 16.002 | <0.0001 |
|  | Denial (t1 vs. t0) | -0.201 | 1.547 | -0.130 | -3.233 | 2.831 | 0.8420 |
|  | Denial (t2 vs. t0) | -3.388 | 1.475 | -2.297 | -6.279 | -0.497 | 0.0104 |
| M1.5 Pooled | Emotional support | -3.025 | 0.624 | -4.848 | -4.248 | -1.802 | <0.0001 |
| M2.5Pooled | Emotional support (t0) | -2.891 | 0.939 | -3.079 | -4.731 | -1.051 | 0.0014 |
|  | Emotional support (t1 vs. t0) | 0.295 | 1.012 | 0.292 | -1.689 | 2.279 | 0.7074 |
|  | Emotional support (t2 vs. t0) | -1.207 | 1.123 | -1.075 | -3.408 | 0.994 | 0.1733 |
| M1.6 Pooled | Humour | -3.811 | 0.703 | -5.421 | -5.189 | -2.433 | <0.0001 |
| M2.6 Pooled | Humour (t0) | -3.518 | 1.018 | -3.456 | -5.513 | -1.523 | 0.0001 |
|  | Humour (t1 vs. t0) | 2.177 | 1.175 | 1.853 | -0.126 | 4.480 | 0.0601 |
|  | Humour (t2 vs. t0) | 0.374 | 1.203 | 0.311 | -1.984 | 2.732 | 0.4411 |
| M1.7 Pooled | Informational support | -0.863 | 0.632 | -1.366 | -2.102 | 0.376 | 0.0813 |
| M2.7 Pooled | Informational support (t0) | -0.158 | 0.977 | -0.162 | -2.073 | 1.757 | 0.9302 |
|  | Informational support (t1 vs. t0) | -0.873 | 1.131 | -0.772 | -3.090 | 1.344 | 0.3523 |
|  | Informational support (t2 vs. t0) | -1.395 | 1.228 | -1.136 | -3.802 | 1.012 | 0.2344 |
| M1.8 Pooled | Planning | 0.212 | 0.623 | 0.340 | -1.009 | 1.433 | 0.5131 |
| M2.8 Pooled | Planning (t0) | 0.283 | 1.039 | 0.272 | -1.753 | 2.319 | 0.3562 |
|  | Planning (t1 vs. t0) | 0.322 | 1.352 | 0.238 | -2.328 | 2.972 | 0.4822 |
|  | Planning (t2 vs. t0) | -3.176 | 1.283 | -2.475 | -5.691 | -0.661 | 0.0080 |
| M1.9 Pooled | Positive reframing | -6.874 | 0.736 | -9.340 | -8.317 | -5.431 | <0.0001 |
| M2.9 Pooled | Positive reframing (t0) | -7.084 | 0.842 | -8.413 | -8.734 | -5.434 | <0.0001 |
|  | Positive reframing (t1 vs. t0) | 0.831 | 1.065 | 0.780 | -1.256 | 2.918 | 0.5211 |
|  | Positive reframing (t2 vs. t0) | -0.923 | 1.252 | -0.737 | -3.377 | 1.531 | 0.6393 |
| M1.10 Pooled | Religion | 0.015 | 0.852 | 0.018 | -1.655 | 1.685 | 0.8772 |
| M2.10 Pooled | Religion (t0) | -0.699 | 0.975 | -0.717 | -2.610 | 1.212 | 0.3672 |
|  | Religion (t1 vs. t0) | 1.212 | 1.037 | 1.169 | -0.821 | 3.245 | 0.2214 |
|  | Religion (t2 vs. t0) | 1.395 | 1.225 | 1.139 | -1.006 | 3.796 | 0.2430 |
| M1.11 Pooled | Self-blame | 11.463 | 0.665 | 17.238 | 10.160 | 12.766 | <0.0001 |
| M2.11 Pooled | Self-blame (t0) | 13.324 | 1.017 | 13.101 | 11.331 | 15.317 | <0.0001 |
|  | Self-blame (t1 vs. t0) | -1.224 | 1.224 | -1.000 | -3.623 | 1.175 | 0.3454 |
|  | Self-blame (t2 vs. t0) | -2.283 | 1.231 | -2.019 | -4.500 | 0.130 | 0.0883 |
| M1.12 Pooled | Self-distraction | 2.708 | 0.693 | 3.908 | 1.350 | 4.066 | 0.0003 |
| M2.12 Pooled | Self-distraction (t0) | 2.145 | 0.935 | 2.294 | 0.312 | 3.978 | 0.0293 |
|  | Self-distraction (t1 vs. t0) | 0.318 | 1.316 | 0.242 | -2.261 | 2.897 | 0.6114 |
|  | Self-distraction (t2 vs. t0) | -1.110 | 1.074 | -1.034 | -3.215 | 0.995 | 0.3032 |
| M1.13 Pooled | Substance use | 12.440 | 0.942 | 13.206 | 10.594 | 14.286 | <0.0001 |
| M2.13 Pooled | Substance use (t0) | 12.249 | 1.075 | 11.394 | 10.142 | 14.356 | <0.0001 |
|  | Substance use (t1 vs. t0) | 1.542 | 1.218 | 1.266 | -0.845 | 3.929 | 0.4752 |
|  | Substance use (t2 vs. t0) | -0.912 | 1.251 | -0.729 | -3.364 | 1.540 | 0.1964 |
| M1.14 Pooled | Venting | 1.153 | 0.723 | 1.595 | -0.264 | 2.570 | 0.1921 |
| M2.14 Pooled | Venting (t0) | 2.487 | 1.007 | 2.470 | 0.513 | 4.461 | 0.0027 |
|  | Venting (t1 vs. t0) | -1.404 | 1.133 | -1.239 | -3.625 | 0.817 | 0.1830 |
|  | Venting (t2 vs. t0) | -2.822 | 1.311 | -2.153 | -5.392 | -0.252 | 0.0035 |

Abbreviations. CI=confidence interval; S.E.=standard error; df=degree of freedom; LB=lower bound; UB=upper bound; M1= Univariate model, including each coping response separately; M2=Univariate model, including the interaction with time and each coping response separately

Linear mixed model specifications: independent variable = coping response; dependent variable = psychological distress; covariates: age, gender, residence; Participants were considered subjects, time of assessment as factor. Parameter estimates are based on the restricted maximum likelihood (REML) method. The variance-covariance structure of the random effects was specified as unstructured (UN), the variance-covariance structure of the within-group residuals (time) was specified as first-order autoregressive (AR1)

# **Supplementary Table 7.** Pooled estimates of fixed effects in the multivariable Linear Mixed Model after multiple imputation procedure

| **Model** | **Coping response** | **Estimate** | **S.E.** | **t** | **95% CI (Estimate)** | | **p-value** |
| --- | --- | --- | --- | --- | --- | --- | --- |
|  |  |  |  |  | **LB** | **UB** |  |
| M3 Pooled | Acceptance | -2.722 | 0.608 | -4.477 | -3.914 | -1.530 | <0.0001 |
| M4 Pooled | Acceptance (t0) | -2.174 | 1.045 | -2.080 | -4.222 | -0.126 | 0.0221 |
|  | Acceptance (t1 vs. t0) | 1.413 | 1.141 | 1.238 | -0.823 | 3.649 | 0.2122 |
|  | Acceptance (t2 vs. t0) | 0.002 | 1.378 | 0.001 | -2.699 | 2.703 | 0.9994 |
| M3 Pooled | Active coping | -3.488 | 0.671 | -5.198 | -4.803 | -2.173 | <0.0001 |
| M4 Pooled | Active coping (t0) | -2.747 | 1.115 | -2.464 | -4.932 | -0.562 | 0.0122 |
|  | Active coping (t1 vs. t0) | 1.237 | 1.295 | 0.955 | -1.301 | 3.775 | 0.7021 |
|  | Active coping (t2 vs. t0) | -0.039 | 2.370 | -0.016 | -4.684 | 4.606 | 0.9870 |
| M3 Pooled | Behavioural disengagement | 5.196 | 0.764 | 6.801 | 3.699 | 6.693 | <0.0001 |
| M4 Pooled | Behavioural disengagement (t0) | 6.171 | 1.255 | 4.917 | 3.711 | 8.631 | <0.0001 |
|  | Behavioural disengagement (t1 vs. t0) | 1.978 | 1.279 | 1.547 | -0.529 | 4.485 | 0.1872 |
|  | Behavioural disengagement (t2 vs. t0) | -1.755 | 2.155 | -0.814 | -5.979 | 2.469 | 0.5823 |
| M3 Pooled | Denial | 7.354 | 0.792 | 9.285 | 5.802 | 8.906 | <0.0001 |
| M4 Pooled | Denial (t0) | 6.642 | 1.389 | 4.782 | 3.920 | 9.364 | 0.0002 |
|  | Denial (t1 vs. t0) | 1.363 | 1.894 | 0.720 | -2.349 | 5.075 | 0.4183 |
|  | Denial (t2 vs. t0) | -2.756 | 1.903 | -1.448 | -6.486 | 0.974 | 0.1084 |
| M3 Pooled | Emotional support | -2.948 | 0.609 | -4.841 | -4.142 | -1.754 | <0.0001 |
| M4 Pooled | Emotional support (t0) | -3.213 | 1.085 | -2.961 | -5.340 | -1.086 | 0.0031 |
|  | Emotional support (t1 vs. t0) | 0.596 | 1.563 | 0.381 | -2.467 | 3.659 | 0.5182 |
|  | Emotional support (t2 vs. t0) | -0.037 | 1.607 | -0.023 | -3.187 | 3.113 | 0.9891 |
| M3 Pooled | Humour | -1.201 | 0.657 | -1.828 | -2.489 | 0.087 | 0.0641 |
| M4 Pooled | Humour (t0) | -1.587 | 0.859 | -1.847 | -3.271 | 0.097 | 0.0563 |
|  | Humour (t1 vs. t0) | 1.245 | 1.283 | 0.970 | -1.270 | 3.760 | 0.5894 |
|  | Humour (t2 vs. t0) | 0.319 | 1.727 | 0.185 | -3.066 | 3.704 | 0.9082 |
| M3 Pooled | Positive reframing | -3.336 | 0.706 | -4.725 | -4.720 | -1.952 | <0.0001 |
| M4 Pooled | Positive reframing (t0) | -3.717 | 0.981 | -3.789 | -5.640 | -1.794 | 0.0002 |
|  | Positive reframing (t1 vs. t0) | 0.386 | 1.337 | 0.289 | -2.235 | 3.007 | 0.8693 |
|  | Positive reframing (t2 vs. t0) | -1.521 | 2.189 | -0.695 | -5.811 | 2.769 | 0.6381 |
| M3 Pooled | Self-blame | 8.498 | 0.626 | 13.575 | 7.271 | 9.725 | <0.0001 |
| M4 Pooled | Self-blame (t0) | 9.282 | 1.270 | 7.309 | 6.793 | 11.771 | <0.0001 |
|  | Self-blame (t1 vs. t0) | -1.445 | 1.547 | -0.934 | -4.477 | 1.587 | 0.5890 |
|  | Self-blame (t2 vs. t0) | 0.872 | 1.685 | 0.518 | -2.431 | 4.175 | 0.7050 |
| M3 Pooled | Self-distraction | 2.038 | 0.660 | 3.088 | 0.744 | 3.332 | 0.0038 |
| M4 Pooled | Self-distraction (t0) | 3.875 | 0.973 | 3.983 | 1.968 | 5.782 | 0.0031 |
|  | Self-distraction (t1 vs. t0) | -0.998 | 1.145 | -0.872 | -3.242 | 1.246 | 0.4643 |
|  | Self-distraction (t2 vs. t0) | -2.297 | 1.379 | -1.666 | -5.000 | 0.406 | 0.0614 |
| M3 Pooled | Substance use | 8.829 | 0.802 | 11.009 | 7.257 | 10.401 | <0.0001 |
| M4 Pooled | Substance use (t0) | 8.146 | 1.013 | 8.041 | 6.161 | 10.131 | <0.0001 |
|  | Substance use (t1 vs. t0) | 1.872 | 1.206 | 1.552 | -0.492 | 4.236 | 0.0742 |
|  | Substance use (t2 vs. t0) | 0.453 | 1.472 | 0.308 | -2.432 | 3.338 | 0.4011 |

Abbreviations. CI=confidence interval; S.E.=standard error; df=degree of freedom; LB=lower bound; UB=upper bound; M3= Multivariable model, including the combined effect of coping responses; M4= Multivariable model, including the interaction with time and coping responses

Linear mixed model specifications: independent variable = coping responses; dependent variable = psychological distress; covariates: age, gender, residence; Participants were considered subjects, time of assessment as factor. Parameter estimates are based on the restricted maximum likelihood (REML) method. The variance-covariance structure of the random effects was specified as unstructured (UN), the variance-covariance structure of the within-group residuals (time) was specified as first-order autoregressive (AR1)

Note. In both models the following independent variables were excluded due to non-significant results in the univariate analysis: *Informational support*, *planning*, *religion*, and *venting*

# **Supplementary Table 8.** Exposure and propensity to violence separated by men and women

| **Gender** | **Variable** | **Measurement** | **N (%)** | **Statistics**^†^ | **Effect size**^§^ | **p-value** |
| --- | --- | --- | --- | --- | --- | --- |
| Men | Exposed to increased violence since the outbreak of the COVID-19 pandemic | t_0_  t_1_  t_2_ | 3/ 169 (1.8%)  7/ 158 (4.4%)  9/ 144 (6.3%) | 0 / (0+1)  0 / (0+0)  0 / (0+0) | ~0.00  ~0.00  ~0.00 | t_0_-t_1:_ ~1.000  t_0_-t_2:_ ~1.000  t_1_-t_2:_ ~1.000 |
| Women | Exposed to increased violence since the outbreak of the COVID-19 pandemic | t_0_  t_1_  t_2_ | 16/ 429 (3.7%)  17/ 408 (4.2%)  24/ 383 (6.3%) | 0 / (0+2)  2 / (2+1)  2 / (2+1) | ~0.00  0.17  0.17 | t_0_-t_1:_ 0.500  t_0_-t_2:_ ~1.000  t_1_-t_2:_ ~1.000 |
| Men | Propensity for violence has increased since the outbreak of the COVID-19 pandemic | t_0_  t_1_  t_2_ | 17/ 169 (10.1%)  28/ 158 (17.7%)  31/ 144 (21.5%) | 9 / (9+11)  7 / (7+15)  7 / (7+13) | 0.05  0.18  0.15 | t_0_-t_1:_ 0.824  t_0_-t_2:_ 0.134  t_1_-t_2:_ 0.263 |
| Women | Propensity for violence has increased since the outbreak of the COVID-19 pandemic | t_0_  t_1_  t_2_ | 56/ 429 (13.1%)  61/ 408 (15.0%)  67/ 383 (17.5%) | χ^2^(1) = 0.09  χ^2^(1) = 3.25  χ^2^(1) = 1.69 | 0.03  0.13  0.10 | t_0_-t_1:_ 0.770  t_0_-t_2:_ 0.071  t_1_-t_2:_ 0.194 |

^†^ For the McNemar test a χ^2^ distribution was used, when the number of observations was at least *n*=35. Else the binomial distribution was used and the cell counts for the 2x2 table in the form of (b/(b + c)) were reported.
^§^ Cohen’s *g* is calculated with |(b/(b + c)) – 0.5|

# **Supplementary Table 9.** Means, and standard deviations of BSCL scales at baseline (t0) and follow-up (t1 & t2) for men and women

| **Scales** | **Measurement** | **Number of items** | **Men**  **Mean** (SD) | **Women**  **Mean** (SD) | **p_BH_-value**^†^ |
| --- | --- | --- | --- | --- | --- |
| Anger-hostility | t_0_  t_1_  t_2_ | 5 | 2.05 (2.58)  2.65 (3.12)  2.22 (2.87) | 3.07 (3.23)  2.91 (3.00)  2.57 (2.96) | 0.0009 |
|  |  |  |  |  | 0.3699 |
|  |  |  |  |  | 0.3330 |
| Anxiety | t_0_  t_1_  t_2_ | 6 | 2.42 (3.39)  2.74 (3.65)  2.53 (3.57) | 3.37 (4.14)  3.45 (4.12)  3.13 (3.87) | 0.0246 |
|  |  |  |  |  | 0.0884 |
|  |  |  |  |  | 0.1102 |
| Depression | t_0_  t_1_  t_2_ | 6 | 3.34 (4.67)  3.74 (4.75)  3.47 (4.32) | 4.05 (4.78)  4.02 (4.80)  3.71 (4.78) | 0.3039 |
|  |  |  |  |  | 0.5869 |
|  |  |  |  |  | 0.5869 |
| Paranoid ideation | t_0_  t_1_  t_2_ | 5 | 2.83 (3.39)  3.35 (3.62)  3.00 (3.32) | 3.33 (3.72)  3.39 (3.66)  3.44 (3.68) | 0.3176 |
|  |  |  |  |  | 0.9121 |
|  |  |  |  |  | 0.3176 |
| Phobic anxiety | t_0_  t_1_  t_2_ | 5 | 2.24 (3.14)  2.39 (3.31)  1.77 (2.91) | 2.63 (3.18)  2.67 (3.38)  2.09 (3.19) | 0.3800 |
|  |  |  |  |  | 0.3800 |
|  |  |  |  |  | 0.3800 |
| Psychoticism | t_0_  t_1_  t_2_ | 5 | 1.59 (2.37)  1.92 (2.87)  1.74 (2.33) | 1.96 (2.93)  1.96 (3.05)  1.90 (3.03) | 0.4431 |
|  |  |  |  |  | 0.8892 |
|  |  |  |  |  | 0.8435 |
| Somatization | t_0_  t_1_  t_2_ | 7 | 1.69 (3.21)  2.63 (3.87)  2.62 (3.75) | 3.14 (4.38)  3.35 (4.43)  3.07 (4.06) | 0.0003 |
|  |  |  |  |  | 0.1109 |
|  |  |  |  |  | 0.2452 |
| Interpersonal sensitivity | t_0_  t_1_  t_2_ | 4 | 2.11 (2.64)  2.61 (2.92)  2.21 (2.68) | 2.97 (3.19)  3.18 (3.09)  3.01 (3.19) | 0.0060 |
|  |  |  |  |  | 0.0464 |
|  |  |  |  |  | 0.0113 |
| Obsessive-compulsiveness | t_0_  t_1_  t_2_ | 6 | 3.64 (4.31)  4.66 (4.76)  4.40 (4.18) | 4.47 (4.44)  4.71 (4.41)  4.58 (4.46) | 0.1197 |
|  |  |  |  |  | 0.9084 |
|  |  |  |  |  | 0.9084 |
| Global severity | t_0_  t_1_  t_2_ | 53 | 23.82 (27.70)  28.85 (30.34)  25.88 (27.46) | 31.15 (31.11)  31.85 (31.10)  29.77 (31.06) | 0.0231 |
|  |  |  |  |  | 0.3011 |
|  |  |  |  |  | 0.2819 |

Abbreviations: SD = standard deviation, p_BH_ = Benjamini-Hochberg corrected p-value
† Linear mixed model specifications: Independent variable = Gender; dependent variable = BSCL responses; Participants were considered subjects. Parameter estimates are based on the restricted maximum likelihood (REML) method. The variance-covariance structure of the random effects was specified as unstructured (UN), the variance-covariance structure of the within-group residuals (time) was specified as first-order autoregressive (AR1).

# **Supplementary Table 10.** Means, and standard deviations of Brief COPE scales at baseline (t0) and follow-up (t1 & t2) for men and women

| **Scales** | **Measurement** | **Men**  **Mean** (SD) | **Women**  **Mean** (SD) | **p_BH_-value**^†^ |
| --- | --- | --- | --- | --- |
| Acceptance | t_0_  t_1_  t_2_ | 2.75 (0.76)  2.78 (0.80)  2.78 (0.79) | 2.69 (0.76)  2.76 (0.77)  2.77 (0.79) | 0.8689 |
|  |  |  |  | 0.8689 |
|  |  |  |  | 0.8689 |
| Active coping | t_0_  t_1_  t_2_ | 2.51 (0.74)  2.44 (0.71)  2.48 (0.68) | 2.58 (0.71)  2.58 (0.70)  2.65 (0.68) | 0.2692 |
|  |  |  |  | 0.0507 |
|  |  |  |  | 0.0306 |
| Behavioural disengagement | t_0_  t_1_  t_2_ | 1.55 (0.62)  1.63 (0.63)  1.65 (0.60) | 1.56 (0.57)  1.68 (0.62)  1.72 (0.61) | 0.7516 |
|  |  |  |  | 0.5886 |
|  |  |  |  | 0.5886 |
| Denial | t_0_  t_1_  t_2_ | 1.38 (0.57)  1.41 (0.54)  1.40 (0.53) | 1.46 (0.59)  1.46 (0.60)  1.50 (0.62) | 0.1880 |
|  |  |  |  | 0.3699 |
|  |  |  |  | 0.1880 |
| Emotional support | t_0_  t_1_  t_2_ | 2.27 (0.78)  2.29 (0.80)  2.34 (0.72) | 2.64 (0.75)  2.61 (0.77)  2.55 (0.79) | <0.0001 |
|  |  |  |  | <0.0001 |
|  |  |  |  | 0.0045 |
| Humour | t_0_  t_1_  t_2_ | 2.41 (0.80)  2.36 (0.77)  2.35 (0.83) | 2.23 (0.80)  2.14 (0.75)  2.19 (0.81) | 0.0182 |
|  |  |  |  | 0.0060 |
|  |  |  |  | 0.0540 |
| Informational support | t_0_  t_1_  t_2_ | 2.01 (0.77)  2.01 (0.81)  2.01 (0.74) | 2.25 (0.80)  2.24 (0.79)  2.25 (0.78) | 0.0021 |
|  |  |  |  | 0.0026 |
|  |  |  |  | 0.0026 |
| Planning | t_0_  t_1_  t_2_ | 2.64 (0.75)  2.63 (0.73)  2.56 (0.76) | 2.71 (0.69)  2.76 (0.67)  2.70 (0.70) | 0.2981 |
|  |  |  |  | 0.0716 |
|  |  |  |  | 0.0716 |
| Positive reframing | t_0_  t_1_  t_2_ | 2.55 (0.76)  2.64 (0.73)  2.50 (0.73) | 2.74 (0.78)  2.69 (0.74)  2.65 (0.75) | 0.0204 |
|  |  |  |  | 0.4693 |
|  |  |  |  | 0.0459 |
| Religion | t_0_  t_1_  t_2_ | 1.62 (0.81)  1.45 (0.76)  1.52 (0.76) | 1.68 (0.83)  1.67 (0.80)  1.70 (0.80) | 0.3575 |
|  |  |  |  | 0.0078 |
|  |  |  |  | 0.0398 |
| Self-blame | t_0_  t_1_  t_2_ | 1.55 (0.73)  1.61 (0.78)  1.60 (0.78) | 1.62 (0.76)  1.65 (0.73)  1.70 (0.79) | 0.4545 |
|  |  |  |  | 0.6223 |
|  |  |  |  | 0.4545 |
| Self-distraction | t_0_  t_1_  t_2_ | 2.45 (0.76)  2.48 (0.71)  2.41 (0.71) | 2.56 (0.73)  2.55 (0.70)  2.51 (0.65) | 0.1866 |
|  |  |  |  | 0.3331 |
|  |  |  |  | 0.1866 |
| Substance use | t_0_  t_1_  t_2_ | 1.38 (0.60)  1.52 (0.77)  1.46 (0.77) | 1.33 (0.66)  1.28 (0.60)  1.28 (0.60) | 0.4142 |
|  |  |  |  | 0.0003 |
|  |  |  |  | 0.0071 |
| Venting | t_0_  t_1_  t_2_ | 1.87 (0.71)  1.87 (0.71)  1.81 (0.68) | 2.12 (0.71)  2.13 (0.71)  2.08 (0.69) | 0.0001 |
|  |  |  |  | 0.0001 |
|  |  |  |  | 0.0001 |

Abbreviations: SD = standard deviation. p_BH_ = Benjamini-Hochberg corrected p-value
† Linear mixed model specifications: Independent variable = Gender; dependent variable = Brief COPE responses; Participants were considered subjects. Parameter estimates are based on the restricted maximum likelihood (REML) method. The variance-covariance structure of the random effects was specified as un-structured (UN), the variance-covariance structure of the within-group residuals (time) was specified as first-order autoregressive (AR1).

# **Supplementary Table 11.** Means, and standard deviations of BSCL scales at baseline (t0) and follow-up (t1 & t2) for Tyrol and South-Tyrol

| **Scales** | **Measurement** | **Number of items** | **Tyrol**  **Mean** (SD) | **South-Tyrol**  **Mean** (SD) | **p_BH_-value**^†^ |
| --- | --- | --- | --- | --- | --- |
| Anger-hostility | t_0_  t_1_  t_2_ | 5 | 2.69 (3.13)  2.91 (3.20)  2.54 (3.03) | 2.90 (3.05)  2.71 (2.72)  2.34 (2.78) | 0.4725 |
|  |  |  |  |  | 0.4725 |
|  |  |  |  |  | 0.4725 |
| Anxiety | t_0_  t_1_  t_2_ | 6 | 3.01 (4.11)  3.37 (4.34)  2.93 (3.90) | 3.23 (3.75)  3.04 (3.33)  3.02 (3.62) | 0.7589 |
|  |  |  |  |  | 0.7589 |
|  |  |  |  |  | 0.8024 |
| Depression | t_0_  t_1_  t_2_ | 6 | 3.72 (4.88)  4.12 (4.99)  3.78 (4.77) | 4.03 (4.57)  3.64 (4.40)  3.41 (4.45) | 0.4350 |
|  |  |  |  |  | 0.4350 |
|  |  |  |  |  | 0.4350 |
| Paranoid ideation | t_0_  t_1_  t_2_ | 5 | 3.28 (3.71)  3.62 (3.76)  3.58 (3.63) | 3.06 (3.52)  2.97 (3.39)  2.86 (3.49) | 0.4579 |
|  |  |  |  |  | 0.0588 |
|  |  |  |  |  | 0.0588 |
| Phobic anxiety | t_0_  t_1_  t_2_ | 5 | 2.55 (3.16)  2.81 (3.65)  2.14 (3.24) | 2.49 (3.19)  2.21 (2.77)  1.76 (2.87) | 0.8285 |
|  |  |  |  |  | 0.1164 |
|  |  |  |  |  | 0.2801 |
| Psychoticism | t_0_  t_1_  t_2_ | 5 | 1.92 (2.97)  2.08 (3.30)  1.91 (2.92) | 1.76 (2.50)  1.72 (2.37)  1.75 (2.75) | 0.5211 |
|  |  |  |  |  | 0.5112 |
|  |  |  |  |  | 0.5211 |
| Somatization | t_0_  t_1_  t_2_ | 7 | 2.76 (4.40)  3.40 (4.56)  3.06 (4.01) | 2.70 (3.72)  2.70 (3.76)  2.75 (3.93) | 0.8581 |
|  |  |  |  |  | 0.1842 |
|  |  |  |  |  | 0.5822 |
| Interpersonal sensitivity | t_0_  t_1_  t_2_ | 4 | 2.79 (3.15)  3.19 (3.15)  2.97 (3.14) | 2.64 (2.94)  2.71 (2.85)  2.47 2.93) | 0.5757 |
|  |  |  |  |  | 0.1122 |
|  |  |  |  |  | 0.1122 |
| Obsessive-compulsiveness | t_0_  t_1_  t_2_ | 6 | 4.29 (4.42)  5.04 (4.85)  4.77 (4.47) | 4.16 (4.42)  4.11 (3.79)  4.10 (4.20) | 0.7178 |
|  |  |  |  |  | 0.0519 |
|  |  |  |  |  | 0.1346 |
| Global severity | t_0_  t_1_  t_2_ | 53 | 29.18 (31.62)  32.91 (33.12)  29.97 (30.94) | 28.93 (28.50)  27.72 (26.34)  26.46 (28.62) | 0.9225 |
|  |  |  |  |  | 0.1620 |
|  |  |  |  |  | 0.3006 |

Abbreviations: SD = standard deviation, p_BH_ = Benjamini-Hochberg corrected p-value
† Linear mixed model specifications: Independent variable = Residence; dependent variable = BSCL responses; Participants were considered subjects. Parameter estimates are based on the restricted maximum likelihood (REML) method. The variance-covariance structure of the random effects was specified as unstructured (UN), the variance-covariance structure of the within-group residuals (time) was specified as first-order autoregressive (AR1).

# **Supplementary Table 12.** Means, and standard deviations of Brief COPE scales at baseline (t0) and follow-up (t1 & t2) for Tyrol and South-Tyrol

| **Scales** | **Measurement** | **Tyrol**  **Mean** (SD) | **South-Tyrol**  **Mean** (SD) | **p_BH_-value**^†^ |
| --- | --- | --- | --- | --- |
| Acceptance | t_0_  t_1_  t_2_ | 2.72 (0.78)  2.79 (0.77)  2.82 (0.77) | 2.69 (0.73)  2.73 (0.79)  2.68 (0.81) | 0.5709 |
|  |  |  |  | 0.5609 |
|  |  |  |  | 0.1518 |
| Active coping | t_0_  t_1_  t_2_ | 2.54 (0.72)  2.51 (0.71)  2.59 (0.69) | 2.60 (0.71)  2.59 (0.71)  2.61 (0.67) | 0.4631 |
|  |  |  |  | 0.4631 |
|  |  |  |  | 0.8342 |
| Behavioural disengagement | t_0_  t_1_  t_2_ | 1.54 (0.59)  1.64 (0.63)  1.65 (0.60) | 1.58 (0.58)  1.69 (0.60)  1.77 (0.62) | 0.4058 |
|  |  |  |  | 0.4058 |
|  |  |  |  | 0.0930 |
| Denial | t_0_  t_1_  t_2_ | 1.40 (0.55)  1.46 (0.59)  1.49 (0.59) | 1.49 (0.63)  1.43 (0.59)  1.44 (0.60) | 0.1683 |
|  |  |  |  | 0.6531 |
|  |  |  |  | 0.5309 |
| Emotional support | t_0_  t_1_  t_2_ | 2.57 (0.81)  2.62 (0.81)  2.57 (0.77) | 2.49 (0.73)  2.36 (0.72)  2.36 (0.76) | 0.1965 |
|  |  |  |  | 0.0003 |
|  |  |  |  | 0.0048 |
| Humour | t_0_  t_1_  t_2_ | 2.34 (0.79)  2.27 (0.76)  2.31 (0.84) | 2.20 (0.81)  2.08 (0.76)  2.11 (0.76) | 0.0323 |
|  |  |  |  | 0.0090 |
|  |  |  |  | 0.0090 |
| Informational support | t_0_  t_1_  t_2_ | 2.21 (0.83)  2.20 (0.83)  2.23 (0.79) | 2.14 (0.76)  2.12 (0.75)  2.11 (0.73) | 0.3143 |
|  |  |  |  | 0.3143 |
|  |  |  |  | 0.2778 |
| Planning | t_0_  t_1_  t_2_ | 2.70 (0.70)  2.74 (0.69)  2.68 (0.71) | 2.69 (0.73)  2.71 (0.69)  2.62 (0.73) | 0.8420 |
|  |  |  |  | 0.8420 |
|  |  |  |  | 0.8420 |
| Positive reframing | t_0_  t_1_  t_2_ | 2.64 (0.79)  2.65 (0.75)  2.61 (0.75) | 2.75 (0.76)  2.72 (0.71)  2.62 (0.74) | 0.3126 |
|  |  |  |  | 0.4250 |
|  |  |  |  | 0.9121 |
| Religion | t_0_  t_1_  t_2_ | 1.58 (0.79)  1.55 (0.74)  1.60 (0.76) | 1.78 (0.86)  1.72 (0.87)  1.74 (0.84) | 0.0105 |
|  |  |  |  | 0.0222 |
|  |  |  |  | 0.0424 |
| Self-blame | t_0_  t_1_  t_2_ | 1.58 (0.74)  1.63 (0.73)  1.64 (0.78) | 1.63 (0.77)  1.65 (0.76)  1.73 (0.81) | 0.6212 |
|  |  |  |  | 0.6997 |
|  |  |  |  | 0.5754 |
| Self-distraction | t_0_  t_1_  t_2_ | 2.52 (0.77)  2.56 (0.69)  2.52 (0.66) | 2.54 (0.68)  2.47 (0.71)  2.41 (0.68) | 0.8056 |
|  |  |  |  | 0.2171 |
|  |  |  |  | 0.2171 |
| Substance use | t_0_  t_1_  t_2_ | 1.39 (0.68)  1.41 (0.72)  1.38 (0.70) | 1.28 (0.57)  1.23 (0.53)  1.25 (0.56) | 0.0375 |
|  |  |  |  | 0.0036 |
|  |  |  |  | 0.0375 |
| Venting | t_0_  t_1_  t_2_ | 2.05 (0.75)  2.04 (0.71)  2.00 (0.73) | 2.06 (0.68)  2.09 (0.73)  2.02 (0.65) | 0.8716 |
|  |  |  |  | 0.8716 |
|  |  |  |  | 0.8716 |

Abbreviations: SD = standard deviation. p_BH_ = Benjamini-Hochberg corrected p-value
† Linear mixed model specifications: Independent variable = Residence; dependent variable = Brief COPE responses; Participants were considered subjects. Parameter estimates are based on the restricted maximum likelihood (REML) method. The variance-covariance structure of the random effects was specified as un-structured (UN), the variance-covariance structure of the within-group residuals (time) was specified as first-order autoregressive (AR1).

**References**

Horton. N. J. & Lipsitz. S. R. (2001). Multiple Imputation in Practice. *The American Statistician*. *55*(3). 244–254. https://doi.org/10.1198/000313001317098266

Little. R. J. A. (1988). A Test of Missing Completely at Random for Multivariate Data with Missing Values. *Journal of the American Statistical Association*. *83*(404). 1198–1202. https://doi.org/10.1080/01621459.1988.10478722

Rubin. D. B. (1987). *Multiple Imputation for Nonresponse in Surveys*. Wiley. https://doi.org/10.1002/9780470316696

Van Buuren. S. (2007). Multiple imputation of discrete and continuous data by fully conditional specification. *Statistical Methods in Medical Research*. *16*(3). 219–242. https://doi.org/10.1177/0962280206074463

Van Buuren. S. (2018). *Flexible Imputation of Missing Data. Second Edition* (second edition). Boca Raton. Florida: Chapman and Hall/CRC. https://doi.org/10.1201/9780429492259

Von Hippel. P. T. (2009). How to Impute Interactions. Squares. and other Transformed Variables. *Sociological Methodology*. *39*(1). 265–291. https://doi.org/10.1111/j.1467-9531.2009.01215.x
